# Rasta resin–triphenylphosphine oxides and their use as recyclable heterogeneous reagent precursors in halogenation reactions

**DOI:** 10.3762/bjoc.10.143

**Published:** 2014-06-20

**Authors:** Xuanshu Xia, Patrick H Toy

**Affiliations:** 1 Department of Chemistry, The University of Hong Kong, Pokfulam Road, Hong Kong, People’s Republic of China

**Keywords:** Appel reaction, halogenation, organophosphorus, polymer-supported reagent, rasta resin, triphenylphosphine oxide

## Abstract

Heterogeneous polymer-supported triphenylphosphine oxides based on the rasta resin architecture have been synthesized, and applied as reagent precursors in a wide range of halogenation reactions. The rasta resin–triphenylphosphine oxides were reacted with either oxalyl chloride or oxalyl bromide to form the corresponding halophosphonium salts, and these in turn were reacted with alcohols, aldehydes, aziridines and epoxides to form halogenated products in high yields after simple purification. The polymer-supported triphenylphosphine oxides formed as a byproduct during these reactions could be recovered and reused numerous times with no appreciable decrease in reactivity.

## Introduction

**Scheme 1:**
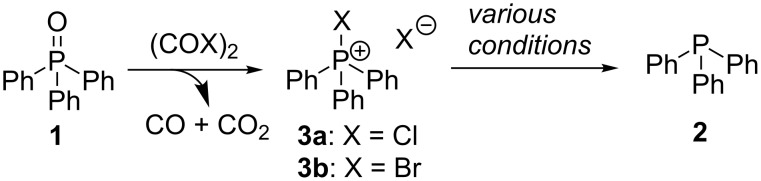
The Masaki–Fukui reaction and halophosphonium salt reduction.

One of the major drawbacks of the Wittig [1] and Mitsunobu [2–3] reactions is that they result in the formation of a stoichiometric quantity of triphenylphosphine oxide (**1**) as a byproduct. From an atom economy perspective this is less than ideal, and from an environmental point of view it would be good if **1** could be simply reduced to triphenylphosphine (**2**) for reuse [4]. In this regard Tanaka and co-workers have studied the possibility of applying the reaction first reported by Masaki and Fukui [5] in which **1** can be treated with oxalyl chloride (or bromide) to form halophosphonium salt **3a** (or **3b**), which in turn can be reduced to **2** under more mild reaction conditions than can **1** (Scheme 1) [6–7].

**Scheme 2:**
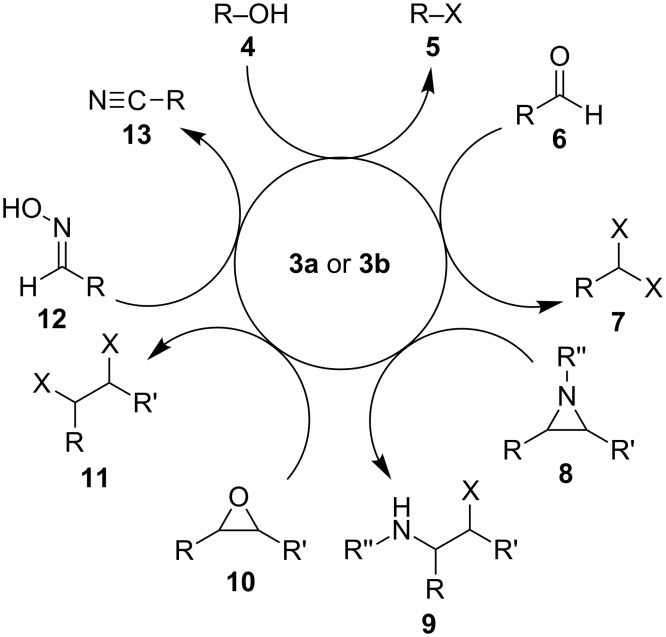
Representative reactions involving halophosphonium salts **3a**,**b**.

In addition to being relatively easily reduced, halophosphonium salts **3a**,**b** are also useful reagents in a wide range of reactions, such as those illustrated in Scheme 2: (1) the conversion of alcohols **4** to alkyl halides **5** (the Appel reaction), (2) the conversion of aldehydes **6** to 1,1-dihaloalkanes **7**, (3) halogenation of aziridines **8** to form 2-haloamines **9**, (4) halogenation of epoxides **10** to form 1,2-dihaloalkanes **11**, (5) and the dehydration of oximes **12** to form nitriles **13**.

**Scheme 3:**
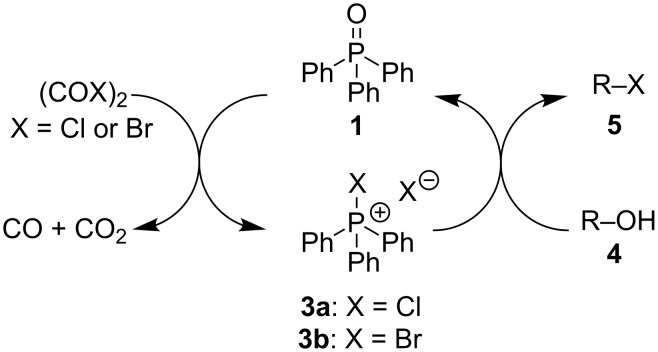
Catalytic Appel reactions reported by Denton and co-workers.

Capitalizing on the fact that **1** is formed as a byproduct from **3a**,**b** in each of these reactions, Denton and co-workers have recently combined the Masaki–Fukui reaction with many of the functional group transformation outlined in Scheme 2, in one-pot processes in which the role of **1** is referred to as that of a catalyst [8–12]. For example, catalytic Appel reactions were achieved by slowly adding separate solutions of oxalyl chloride and alcohols **4** to a solution of **1** (Scheme 3) [8–9]. In these reactions, the simultaneous slow addition of oxalyl chloride and alcohol substrate **4** to a sub-stoichiometric quantity of **3a** was necessary in order to minimize formation of undesired ester side-products formed by the reaction of **4** with the acid chloride. Furthermore, chromatographic purification of the alkyl halide product **5** was required. Thus, while the procedures reported by Denton et al. might be conceptually interesting, they may not be particularly convenient to perform, especially on larger scales than what was originally reported.

**Figure 1:**
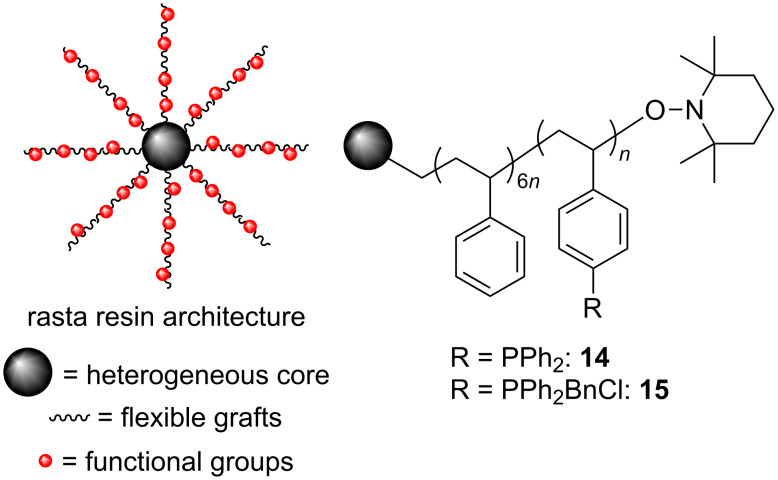
Rasta resins **14** and **15**.

We have had a long-term interest in the use of organic polymers as supports for reagents and catalysts [13], and have reported the use of various polymer-supported phosphines as reagents, organocatalysts, and ligands in order to simplify product isolation [14–18]. Most recently we have studied the use of the rasta resin polystyrene architecture [19–26] as a platform for reagents and catalysts [27–33], and have used easily synthesized rasta resin–Ph_3_P (**14**) in various Wittig reactions that required only filtration and solvent removal for product purification (Figure 1) [27–29]. Additionally, **14** was converted into phosphonium salt **15**, which proved to be an efficient and highly recyclable catalyst for aldehyde and ketone cyanosilylation reactions from which the products could also be obtained pure after only filtration and solvent removal [30]. It should be noted that the grafts of the rasta resins reported are random co-polymers, and the structures drawn for them are not mean to indicate that they are block co-polymers. The format for their presentation is used merely to indicate their monomer incorporation ratios.

Thus, considering our prior success in using **14** and **15**, we wanted to oxidize **14** to **16**, and in turn use this as a heterogeneous precursor to reagents **17a**,**b** for use in the halogenation reactions described in Scheme 2. We anticipated that using a full equivalent **17a**,**b** generated in situ would eliminate the need for slow addition of the oxalyl halide to form the halophosphonium salt, and thus allow for the reactions to be performed more conveniently than in the catalytic procedures of Denton and co-workers. Furthermore, since **16** would be the byproduct of the reactions, it could be recovered by filtration at the end of the reactions and reused directly. Herein we report the realization of this strategy and describe simple procedures for alcohol, aziridine, aldehyde and epoxide halogenation reactions from which the desired products are easily isolated and the phosphine oxide byproduct is readily recycled.

## Results and Discussion

**Scheme 4:**
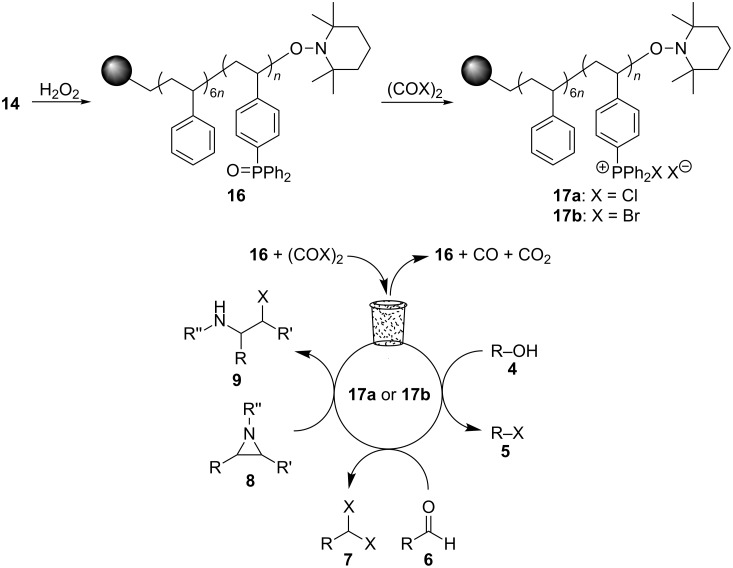
Synthesis and applications of rasta resins **16** and **17a**,**b**.

Rasta resin **16** was prepared by oxidation of **14**, which was prepared as previously reported [28], using H_2_O_2_ (Scheme 4). The loading level of **16** was determined by elemental analysis to be 0.97 mmol/g, and gel-phase ^31^P NMR spectroscopic analysis of **16** showed only a single peak at 29.4 ppm, indicating that the phosphine groups were completely oxidized.

### Appel reactions using **16**

With **16** in hand, we initially used it to perform Appel reactions by first converting it into either **17a** or **17b** in situ (Scheme 4). To do this, **16** was suspended/swollen in dichloromethane, and then the appropriate oxalyl halide was added. Once gas evolution ceased, alcohol **4** was added, and the reaction mixture was heated to reflux. Progress of the reactions was monitored by TLC analysis, and they were all finished in 4–7 hours. Upon completion, the reaction mixtures were cooled to room temperature and then filtered. Finally, the filtrates were concentrated at reduced pressure to afford the desired products that were essentially pure based on ^1^H and ^13^C NMR spectroscopic analyses. Chromatographic purification of the resultant alkyl halide products was not required. As can be seen in Table 1, both primary (entries 1–8) and secondary (entries 9 and 10) benzylic alcohols with various substituents all afforded excellent yield of the corresponding chloride and bromide using this procedure. Similar high yields were also obtained from reactions using primary (Table 1, entries 11–14) and secondary aliphatic alcohols (Table 1, entries 15 and 16). A reaction performed on a scale ten times larger afforded excellent yield as well (Table 1, entry 17). The recovered polymer was washed sequentially with water, MeOH, THF, diethyl ether and hexane. After drying, ^31^P NMR spectroscopy confirmed its identity as **16**.

**Table 1:** Appel reactions using **16**.^a^

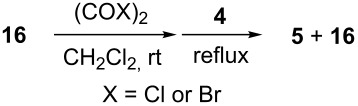					

Entry	Starting material		Product		Yield (%)

1	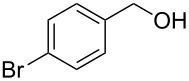	**4A**	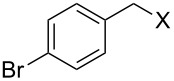	**5Aa**, X = Cl	98
2				**5Ab**, X = Br	92
3	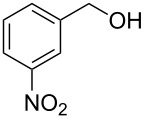	**4B**	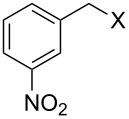	**5Ba**, X = Cl	93
4				**5Bb**, X = Br	91
5	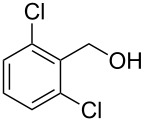	**4C**	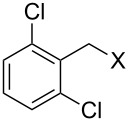	**5Ca**, X = Cl	93
6				**5Cb**, X = Br	90
7	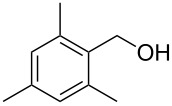	**4D**	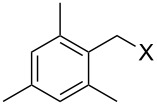	**5Da**, X = Cl	98
8				**5Db**, X = Br	93
9	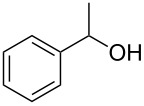	**4E**	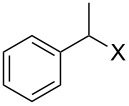	**5Ea**, X = Cl	85
10				**5Eb**, X = Br	89
11	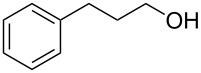	**4F**	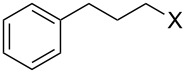	**5Fa**, X = Cl	95
12				**5Fb**, X = Br	90
13	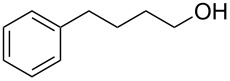	**4G**	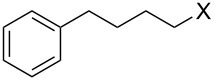	**5Ga**, X = Cl	98
14				**5Gb**, X = Br	92
15	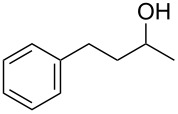	**4H**	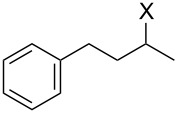	**5Ha**, X = Cl	95
16				**5Hb**, X = Br	91
17^b^				**5Ha**, X = Cl	92

^a^Reaction conditions: 0.6 mmol **16**, 0.6 mmol oxalyl halide, 0.5 mmol **4** in 5 mL CH_2_Cl_2_, reflux. ^b^Reaction conditions: 6 mmol **16**, 6 mmol oxalyl halide, 5 mmol **4** in 50 mL CH_2_Cl_2_, reflux.

### Aldehyde halogenation reactions using **16**

With the success of the Appel reactions, we further examined the utility of **16** by studying its use in aldehyde halogenation reactions. As before, **16** was converted into **17a** or **17b** in situ, and aldehyde **6** was added upon cessation of gas evolution. After 72 hours at reflux, the reactions were stopped, filtered and the products were purified by column chromatography (Table 2). Unlike the Appel reactions discussed above, some of these reactions did not proceed to completion, even when the reaction time was lengthened. Generally, it was observed that the bromination reactions afforded higher product yields than did the corresponding chlorination reactions (Table 2, entries 1–8), except when electron-rich aldehyde starting materials were used (Table 2, entries 9–12). These results are generally similar to what was previously reported by Denton and co-workers when similar substrates were used [12].

**Table 2:** Halogenation reactions of aldehydes using **16**.^a^

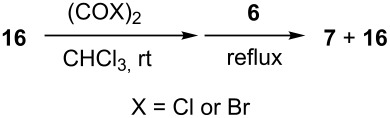					

Entry	Starting material		Product		Yield (%)

1	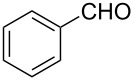	**6A**	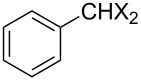	**7Aa**, X = Cl	54^b^
2				**7Ab**, X = Br	75^b^
3	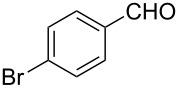	**6B**	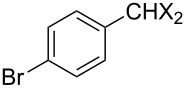	**7Ba**, X = Cl	65^b^
4				**7Bb**, X = Br	83^b^
5	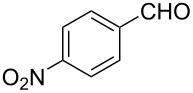	**6C**	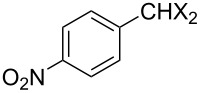	**7Ca**, X = Cl	64^b^
6				**7Cb**, X = Br	89^b^
7	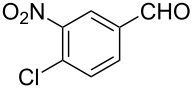	**6D**	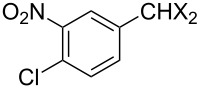	**7Da**, X = Cl	61^b^
8				**7Db**, X = Br	93^b^
9	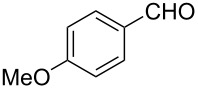	**6E**	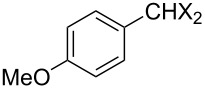	**7Ea**, X = Cl	94^c^
10				**7Eb**, X = Br	20^d^
11	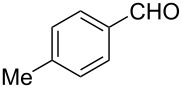	**6F**	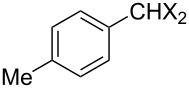	**7Fa**, X = Cl	85^b^
12				**7Fb**. X = Br	77^b^

^a^Reaction conditions: 0.3 mmol **16**, 0.3 mmol oxalyl halide, 0.1 mmol **6** in 3 mL CHCl_3_, reflux. ^b^Isolated yield after flash silica gel column chromatography. ^c^Isolated yield after filtration and concentration under reduced pressure. ^d^Determined by ^1^H NMR spectroscopy.

### Aziridine halogenation reactions using **16**

We next examined the use of **16** as a precursor to **17a** and **17b** in aziridine halogenation reactions [34]. Using reaction conditions similar to those used for the Appel and aldehyde halogenation reactions, a variety of *N*-tosyl aziridines **8** were successfully converted into the corresponding chloro- or bromotosylamides **9** in excellent yields (Table 3). The trans configurations of the **9Ba** and **9Bb** were confirmed by X-ray diffraction analysis of the isolated products, and as was true for the Appel reactions described above, all the products were obtained in high purity simply by filtration to remove the polymer, and concentration of the filtrate. Our procedure was also successfully performed on a gram-scale (Table 3, entry 5), and substrates **8** possessing a single aryl substituent were halogenated as the less hindered position (Table 3, entries 10–13).

**Table 3:** Halogenation reactions of aziridines using **16**.^a^

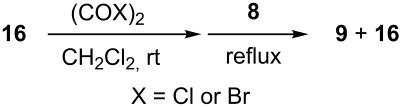					

Entry	Starting material		Product		Yield (%)

1	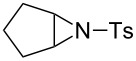	**8A**	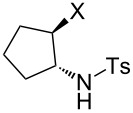	**9Aa**, X = Cl	95
2				**9Ab**, X = Br	93
3	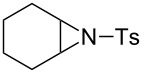	**8B**	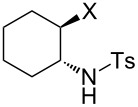	**9Ba**, X = Cl	89
4				**9Bb**, X = Br	92
5^b^				**9Ba**, X = Cl	95
6	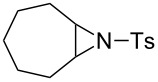	**8C**	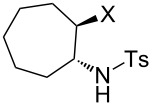	**9Ca**, X = Cl	96
7				**9Cb**, X = Br	98
8	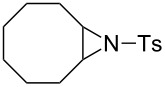	**8D**	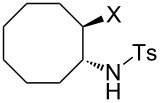	**9Da**, X = Cl	93
9				**9Db**, X = Br	92
10	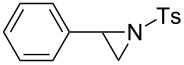	**8E**	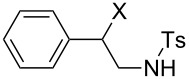	**9Ea**, X = Cl	91
11				**9Eb**, X = Br	93
12	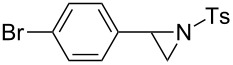	**8F**	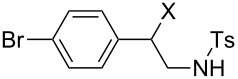	**9Fa**, X = Cl	93
13				**9Fb**, X = Br	95

^a^Reaction conditions: 0.6 mmol **16**, 0.6 mmol oxalyl halide, 0.5 mmol **8** in 5 mL CH_2_Cl_2_, reflux. ^b^Reaction conditions: 6 mmol **16**, 6 mmol oxalyl halide, 5 mmol **8** in 50 mL CH_2_Cl_2_, reflux.

### Recovery and reuse of **16**

After demonstrating that **16** could effectively serve as a precursor to **17a,b**, and realizing that it was returned as the byproduct of the above reactions, we next examined its recyclability in the chlorination of alcohol **4H** and aziridine **8B**. The results of these studies are shown in Table 4 and Table 5, respectively. In these experiments, the polymer recovered by filtration of the reaction mixture was washed and dried, and then used directly for the next reaction cycle. Excellent yields were successfully obtained for 8 runs with both **4H** and **8B**. Gel-phase ^31^P NMR analysis of **16** recovered at the end of these experiments indicated no change in its oxidation state.

**Table 4:** Recycling of **16** in the chlorination of **4H**.^a^

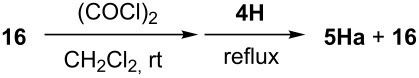								

Run	1	2	3	4	5	6	7	8

Yield of **5Ha** (%)	94	95	93	98	94	95	95	93
Recovery of **16** (%)	99	99	99	99	98	95	93	91

^a^Reaction conditions: 0.6 mmol **16**, 0.6 mmol oxalyl chloride, 0.5 mmol **4H** in 5 mL CH_2_Cl_2_, reflux.

**Table 5:** Recycling of **16** in the chlorination of **8B**.^a^

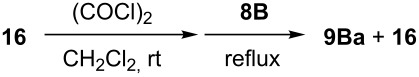								

Run	1	2	3	4	5	6	7	8

Yield of **9Ba** (%)	93	92	95	93	87	91	91	90
Recovery of **16** (%)	96	97	95	95	96	95	90	92

^a^Reaction conditions: 0.6 mmol **16**, 0.6 mmol oxalyl chloride, 0.5 mmol **8B** in 5 mL CH_2_Cl_2_, reflux.

### Epoxide halogenation reactions

**Scheme 5:**
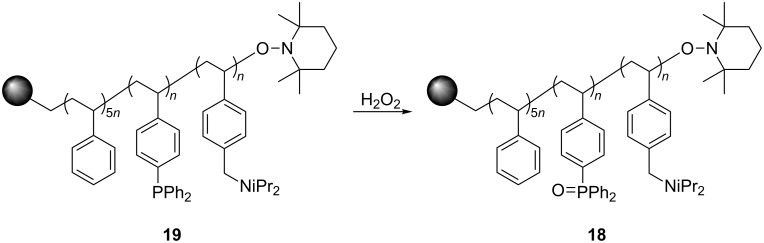
Synthesis of bifuncitonal rasta resin **18**.

With the versatility and excellent reactivity of **16** established, we were encouraged to examine our method in the epoxide halogenation reactions shown in Scheme 2. Since these reactions require the use of a base, we designed a bifunctional rasta resin, RR-NBniPr_2_-PPh_3_=O **18** (Scheme 5), which bears both triphenylphosphine oxide and tertiary amine moieties, in order to increase the efficiency and appeal of our method. We have extensive experience in preparing functionalized resins with two different catalytic groups [35–38], and prepared **18** by oxidation of **19**, which we previously used as a bifunctional reagent in one-pot Wittig reactions [29]. Gel-phase ^31^P NMR spectroscopic analysis of **18** indicated that oxidation of the phosphine groups was complete, and elemental analysis was used to determine the loading level of phosphine oxide and amine groups to be 1.07 mmol/g and 1.06 mmol/g, respectively. It should be noted that a test reaction between *N*,*N*-diisopropylbenzylamine and H_2_O_2_ under similar reaction conditions does not result in amine oxidation, and this seems to indicate that only the phosphine groups of **19** are oxidized during its conversion to **18**.

Having successfully synthesized polymer **18**, we examined its reactivity in epoxide halogenation reactions (Table 6). As before, **18** was suspended/swollen in solvent prior to addition of the oxalyl halide. Once gas evolution ceased, epoxide **10** was added, and the reaction mixture was heated to reflux. When TLC analysis indicated that the reactions were complete, 3–4 hours, the reaction mixtures were filtered and the filtrates were concentrated to afford products that were essentially pure according to both ^1^H and^13^C NMR analysis. Reactions with epoxides bearing phenyl (Table 6, entries 1 and 2), benzyl (Table 6, entries 3 and 4), and alkyl substutients (Table 6, entries 5–8) all proceeded to completion, and afforded the corresponding 1,2-dihalides in excellent yields.

**Table 6:** Halogenation reactions of epoxides using **18**.^a^

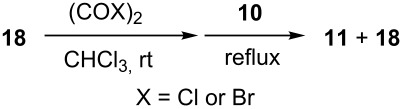					

Entry	Substrate		Product		Yield (%)

1	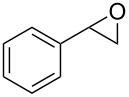	**10A**	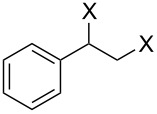	**11Aa**, X = Cl	95
2				**11Ab**, X = Br	93
3	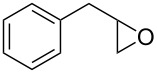	**10B**	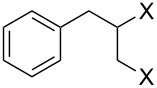	**11Ba**, X = Cl	89
4				**11Bb**, X = Br	92
5	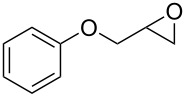	**10C**	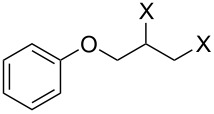	**11Ca**, X = Cl	95
6				**11Cb**, X = Br	96
7	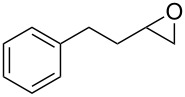	**10D**	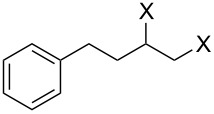	**11Da**, X = Cl	98
8				**11Db**, X = Br	92

^a^Reaction conditions: 1.2 mmol **18**, 1.1 mmol oxalyl halide, 0.5 mmol **10** in 5 mL CHCl_3_, reflux.

We also examined the recyclability of **18** in the halogenation of epoxide **10C** (Table 7). As was the case for **16**, bifunctional polymer **18** could be repeatedly recovered and reused without an observable decrease in its reactivity. However, whereas **16** could be reused directly after recover, reusing **18** required washing it with an aqueous solution of Na_2_CO_3_ after each reaction.

**Table 7:** Recycling of **18** in the chlorination of **10c**.^a^

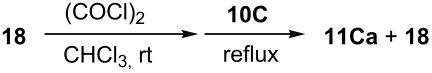							

Run	1	2	3	4	5	6	7

Yield of **11Ca** (%)	94	91	93	94	93	92	90
Recovery of **18** (%)	98	98	97	98	95	93	90

^a^Reaction conditions: 1.2 mmol **18**, 1.1 mmol oxalyl chloride, 0.5 mmol **10C** in 5 mL CHCl_3_, reflux.

## Conclusion

In summary, we have designed and synthesized recyclable heterogeneous rasta resin-supported triphenylphosphine oxide **16**, and have applied it as a phosphonium halide salt precursor in a wide range of halogenation reactions from which it is readily recovered and reused. The reusability of this polymer was demonstrated by the fact that all of the reactions reported herein involving **16** were performed by repeatedly reusing a single batch of it. We also prepared bifunctional rasta resin **18** which bears amine groups in addition to the phosphine oxide moieties for use in epoxide halogenation reactions. The products of all of these reactions, except for the aldehyde halogenation reactions, can be easily separated from the phosphine oxide functionalized polymer simply by filtration, and isolated directly in high purity. Thus, the use of polymers **16** or **18** as reagent precursors represents a convenient alternative to the “phosphine oxide-catalyzed” methods of Denton and co-workers, which generally require slow addition of the oxalyl halide and chromatographic purification of the product. In order to assess the overall utility of **16** and **18**, we are currently examining their applications as organocatalysts [39–43], and will report results of these studies in due course.

## Experimental

**General procedure for Appel reactions using 16:** Polymer **16** (0.6 g, 0.6 mmol) was suspended in dichloromethane (5 mL), and the oxalyl halide (0.6 mmol) was added. Upon cessation of gas evolution, alcohol **4** (0.5 mmol) was added, and the reaction mixture magnetically stirred and heated to reflux. After the reaction was completed as monitored by TLC, the mixture was cooled to room temperature and filtered. The solid on funnel was washed with dichloromethane (10 mL × 3). The solvent of filtrate was removed under reduced pressure to afford the desired product **5** in an essentially pure state based on ^1^H and ^13^C NMR spectroscopic analyses.

**General procedure for aldehyde halogenation reactions using 16:** Polymer **16** (0.3 g, 0.3 mmol) was suspended in chloroform (3 mL), and the oxalyl halide was added (0.3 mmol). Upon cessation of gas evolution, aldehyde **6** (0.1 mmol) was added, and the reaction mixture was magnetically stirred and heated to reflux. After 72 hours, the reaction was cooled to room temperature and filtered. The solid on funnel was washed with dichloromethane (5 mL × 3). The solvent of filtrate was removed under reduced pressure. The resulting crude product **7** was purified by flash silica gel column chromatography using ethyl acetate and hexane as eluent mixture.

**General procedure for aziridine halogenation reactions of using 16:** Polymer **16** (0.6 g, 0.6 mmol) was suspended in dichloromethane (5 mL), and the oxalyl halide was added (0.6 mmol). Upon cessation of gas evolution, aziridine **8** (0.5 mmol) was added, and the reaction mixture was magnetically stirred and heated to reflux. After the reaction was completed as monitored by TLC, the mixture was cooled to room temperature and filtered. The solid on funnel was washed with dichloromethane (10 mL × 3). The solvent of filtrate was removed under reduced pressure to afford the desired product **9** in an essentially pure state based on ^1^H and ^13^C NMR spectroscopic analyses.

**General procedure for epoxide halogenation reactions using 18:** Polymer **18** (1.3 g, 1.2 mmol) was suspended in chloroform (10 mL) and the oxalyl halide was added (1.1 mmol). Upon cessation of gas evolution, epoxide **10** (0.5 mmol) was added, and the reaction was magnetically stirred and heated to reflux. After the reaction was completed as monitored by TLC, the mixture was cooled to room temperature and filtered. The solid on funnel was washed with dichloromethane (10 mL × 3). The solvent of filtrate was removed under reduced pressure to afford the desired product **11** in an essentially pure state based on ^1^H and ^13^C NMR spectroscopic analyses.

**General procedure for recovery and reuse of 16 and 18:** After being separated from the reaction mixture by filtration, the polymer, **16** or **18**, was rinsed sequentially using deionized water (30 mL), dichloromethane (50 mL), MeOH (50 mL), THF (50 mL), diethyl ether (50 mL), hexane (50 mL). It was then dried under vacuum at 60 °C prior to use in the next reaction cycle. Furthermore, **18** was initially washed with a saturated aqueous solution of Na_2_CO_3_ in order to ensure that it was deprotonated and ready for use in the next reaction cycle.

### Note Added in Proof

After the initial submission of our manuscript we became aware of a recent report by Denton and co-workers regarding similar work using polystyrene-supported halophosphonium salts in Appel and dehydration reactions [44]. This work utilized a commercially available polymer-supported phosphine oxide based on the Merrifield resin architecture, and it is noteworthy that the reactions reported in this manuscript required a 6-fold excess of the halophosphonium salt compared to the substrate. Use of our rasta resins **16** and **18** required only a 20 mol % excess.

## Supporting Information

File 1 Additional experimental details and characterization data of synthesized compounds.
