# Supplementary material for: Rasta resin–triphenylphosphine oxides and their use as recyclable heterogeneous reagent precursors in halogenation reactions
Source: Beilstein J Org Chem. 2014 Jun 20;10:1397–405. doi: 10.3762/bjoc.10.143 (PMC4077539; doi:10.3762/bjoc.10.143)
Supplement: File 1 — Additional experimental details and characterization data of synthesized compounds. [file Beilstein_J_Org_Chem-10-1397-s001.pdf]

Supporting Information

for

Rasta resin–triphenylphosphine oxides and their use as recyclable heterogeneous reagent precursors in halogenation reactions

Xuanshu Xia and Patrick H. Toy*

Address: Department of Chemistry, The University of Hong Kong, Pokfulam Road, Hong Kong, People's Republic of China

Email: Patrick H. Toy* - phtoy@hku.hk

* Corresponding author

Additional experimental details and characterization data of synthesized compounds

Contents	Page
General experimental details	S2
Synthesis of RR-PPh_3=O (**16**)	S2
Synthesis of RR-NBniPr_2-PPh_3=O (**18**)	S3
General procedure for Appel reactions using **16** and product characterization data	S3
General procedure for aldehyde halogenation reactions using **16** and product characterization data	S8
General procedure for aziridine halogenation reactions of using **16** and product characterization data	S12
General procedure for epoxide halogenation reactions using **18** and product characterization data	S17
References	S21
^{31}P, ^{1}H and ^{13}C NMR Spectra	S22
X-ray structure details	S72

General experimental details

All reagents were obtained from the Acros, Aldrich, and Alfa Aesar companies, and were used as received. All reactions were carried out in dry glassware under a N_2 atmosphere, and were monitored by TLC analysis using GF_{254} silica gel coated plates. ^1H and ^{13}C NMR spectra were recorded in $CDCl_3$ on a Bruker DRX-300 or DRX-400 spectrometer operating at 300/400 MHz for ^1H and 75/100 MHz for ^{13}C analysis. Gel-phase ^{31}P NMR analysis was performed on a Brucker DRX-400 spectrometer operating at 162 MHz. Chemical shift data is expressed in ppm with reference to TMS. Elemental analysis was performed at the Shanghai Institute of Organic Chemistry. EIMS data was recorded on a Finnigan MAT 96 mass spectrometer. X-ray diffraction data was collected on a Bruker Smart Apex II CCD.

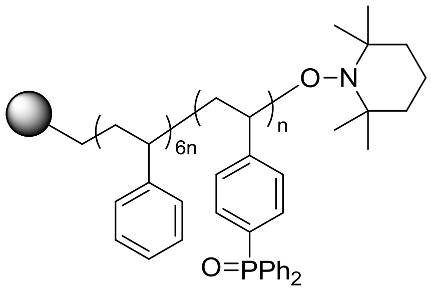

16

Synthesis of 16

Rasta resin-PPh_3 **14** [1] (25.0 g, 24.3 mmol) was added to dichloromethane (50 mL) and 50% hydrogen peroxide (50 mL). The reaction mixture was magnetically stirred at room temperature for 1 h. The resin was then filtered, washed sequentially with CH_2Cl_2 (3 × 100 mL), methanol (3 × 100 mL), THF (3 × 100 mL), diethyl ether (3 × 100 mL) and hexane (3 × 100 mL), and then dried under vacuum at 60 ºC for 16 h to afford **16** as white free-flowing beads (100% yield). Elemental analysis was used to determine the phosphine content (3.0%), and thus a loading level of 0.97 mmol PPh_3/g. A peak at δ 29.4 was observed by gel-phase ^{31}P NMR analysis.

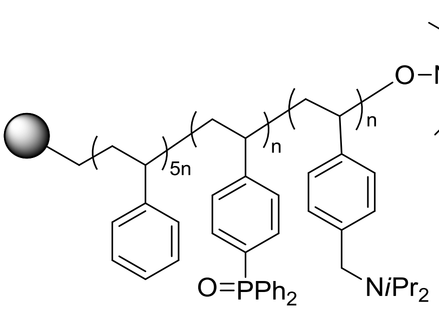

18

Synthesis of 18

Rasta resin-NBniPr$_2$-PPh$_3$ **19** [2] (20.0 g, 18.8 mmol) was added to dichloromethane (50 mL) and 50% hydrogen peroxide (50 mL). The reaction mixture was magnetically stirred at room temperature for 1 h. The resin was then filtered, washed sequentially with CH_2Cl_2 (3 × 100 mL), methanol (3 × 100 mL), THF (3 × 100 mL), diethyl ether (3 × 100 mL) and hexane (3 × 100 mL) and then dried under vacuum at 60 ºC for 16 h to afford **18** as white free-flowing beads (100% yield). Elemental analysis was used to determine the phosphine content (3.3%), and nitrogen content (1.5%,), and thus loading levels of 1.07 mmol PPh$_3$/g, 1.06 mmol NiPr$_2$/g. A peak at δ 29.8 was observed by gel-phase ^{31}P NMR analysis.

General procedure for Appel reactions using 16

To **16** (0.6 g, 0.6 mmol) was added dichloromethane (5 mL) in a round-bottom flask. After 10 min, oxalyl chloride or oxalyl bromide was added (0.6 mmol). The reaction mixture was magnetically stirred at room temperature. Upon cessation of gas evolution, **4** was added (0.5 mmol), and the reaction mixture was heated to reflux. After the reaction was complete according to TLC analysis, the mixture was cooled to room temperature and filtered. The solid on the funnel was washed with dichloromethane (3 × 10 mL), and the filtrate was concentrated under reduced pressure to afford the desired product **5** in an essentially pure state based on ^{1}H and ^{13}C NMR spectroscopic analyses.

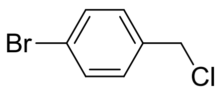

5Aa

1-Bromo-4-(chloromethyl)benzene (5Aa, Table 1, entry 1) [3]**.** Isolated yield: 98%. ^{1}H-NMR (400 MHz, $CDCl_3$) δ 4.52 (s, 2H), 7.25 (d, 2H, J = 8.4 Hz), 7.48 (d, 2H, J = 8.4 Hz); ^{13}C-NMR (100 MHz, $CDCl_3$) δ 45.5, 122.6, 130.4, 132.0, 136.6; MS for C_7H_6BrCl: calcd 204.0, found 204.0.

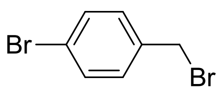

5Ab

1-Bromo-4-(bromomethyl)benzene (5Ab, Table 1, entry 2) [4]**.** Isolated yield: 92%. ^{1}H-NMR (300 MHz, $CDCl_3$) δ 4.44 (s, 2H), 7.27 (d, 2H, J = 8.4 Hz), 7.47 (d, 2H, J = 8.4 Hz); ^{13}C-NMR (100 MHz, $CDCl_3$) δ 32.5, 122.6, 130.8, 132.1, 136.9; MS for $C_7H_6Br_2$: calcd 247.9, found 247.9.

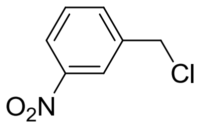

5Ba

1-(Chloromethyl)-3-nitrobenzene (**5Ba, Table 1, entry 3**) [3]**.** Isolated yield: 93%. ^{1}H-NMR (300 MHz, $CDCl_3$) δ 4.67 (s, 2H), 7,56 (t, 1H, J = 7.8 Hz), 7.74 (d, 1H, J = 7.6 Hz) 8.20 (d, 1H, J = 8.2 Hz) 8.28 (s, 1H); ^{13}C-NMR (100 MHz, $CDCl_3$) δ 44.7, 123.5, 123.6, 130.0, 134.6, 148.5; MS for $C_7H_6ClNO_2$: calcd 171.0, found 171.0.

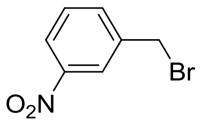

5Bb

1-(Bromomethyl)-3-nitrobenzene (**5Bb, Table 1, entry 4**) [4]. Isolated yield: 91%. ^{1}H-NMR (400 MHz, $CDCl_3$) δ 4.55 (s, 2H), 7.55 (t, 1H, J = 7.9 Hz), 7.74 (d, 1H, J = 7.7 Hz), 8.16 (d, 1H, J = 8.2 Hz), 8.26 (s, 1H); ^{13}C-NMR

(100 MHz, $CDCl_3$) δ 31.4, 123.4, 124.1, 130.1, 135.2, 139.9, 148.5; MS for $C_7H_6BrNO_2$: calcd 216.0, found 215.0.

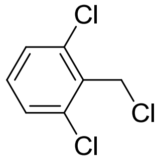

5Ca

1,3-Dichloro-2-(chloromethyl)benzenea (5Ca, Table 1, entry 5) [5]. Isolated yield: 93%. ^{1}H-NMR (400 MHz, $CDCl_3$) δ 4.88 (s, 2H), 7.19-7.35 (m, 3H); ^{13}C-NMR (100 MHz, $CDCl_3$) δ 40.9, 128.7, 130.4, 133.6, 136.3; MS for $C_7H_5Cl_3$: calcd 193.9, found 193.9.

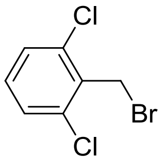

5Cb

2-(Bromomethyl)-1,3-dichlorobenzene (5Cb, Table 1, entry 6) [5]. Isolated yield: 90%. ^{1}H-NMR (400 MHz, $CDCl_3$) δ 4.76 (s, 2H), 7.17-7.34 (m, 3H); ^{13}C-NMR (100 MHz, $CDCl_3$) δ 27.6, 128.7, 130.2, 133.8, 136.1; MS for $C_7H_5BrCl_2$: calcd 237.9, found 237.9.

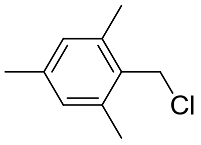

5Da

2-(Chloromethyl)-1,3,5-trimethylbenzene (5Da, Table 1, entry 7) [5]. Isolated yield: 98%. ^{1}H-NMR (300 MHz, $CDCl_3$) δ 2.26 (s, 3H), 2.39 (s, 6H), 4.65 (s, 2H), 6.87 (s, 2H); ^{13}C-NMR (100 MHz, $CDCl_3$) δ 19.3, 21.2, 41.3, 129.4, 131.2, 137.6, 138.6; MS for $C_{10}H_{13}Cl$: calcd 168.1, found 168.1.

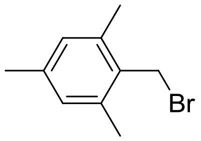

5Db

2-(Bromomethyl)-1,3,5-trimethylbenzene (5Db, Table 1, entry 8) [6]**.** Isolated yield: 93%. ^{1}H-NMR (300 MHz, $CDCl_3$) δ 2.26 (s, 3H), 2.38 (s, 6H), 4.56 (s, 2H), 6.85 (s, 2H); ^{13}C-NMR (100 MHz, $CDCl_3$) δ 19.3, 21.2, 29.9, 129.4, 131.2, 137.6, 138.7; MS for $C_{10}H_{13}Br$: calcd 212.0, found 212.0.

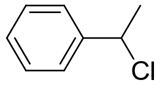

5Ea

(1-Chloroethyl)benzene (5Ea, Table 1, entry 9) [7]**.** Isolated yield: 85%. ^{1}H-NMR (400 MHz, $CDCl_3$) δ 1.86 (d, 3H, *J* = 6.8 Hz), 5.10 (q, 1H, *J* = 6.8 Hz), 7.30-7.43 (m, 5H); ^{13}C-NMR (100 MHz, $CDCl_3$) δ 26.6, 58.9, 126.6, 128.4, 128.7, 142.9; MS for C_8H_9 (C_8H_9Cl): calcd 105.1, found 105.1.

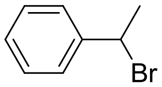

5Eb

(1-Bromoethyl)benzene (5Eb, Table 1, entry 10) [5]**.** Isolated yield: 89%. ^{1}H-NMR (400 MHz, $CDCl_3$) δ 2.04 (d, 3H, *J* = 6.9 Hz), 5.21 (q, 1H, *J* = 6.9 Hz), 7.24-7.44 (m, 5H); ^{13}C-NMR (100 MHz, $CDCl_3$) δ 27.0, 49.8, 127.0, 128.5, 128.8, 143.4; MS for C_8H_9 (C_8H_9Br): calcd 105.1, found 105.1.

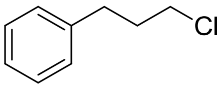

5Fa

(3-Chloropropyl)benzene (5Fa, Table 1, entry 11) [5]**.** Isolated yield: 95%. ^{1}H-NMR (400 MHz, $CDCl_3$) δ 2.05-2.12 (m, 2H), 2.78 (t, 2H, *J* = 7.2 Hz), 3.53 (t, 2H, *J* = 6.3 Hz), 7.19-7.31 (m, 5H); ^{13}C-NMR (100 MHz,

CDCl$_3$) δ 32.9, 34.1, 44.4, 126.3, 128.6, 128.7, 140.8; MS for $C_9H_{11}Cl$: calcd 154.1, found 154.1.

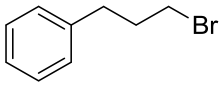

5Fb

(3-Bromopropyl)benzene (5Fb, Table 1, entry 12) [8]. Isolated yield: 90%. ^{1}H-NMR (300 MHz, CDCl$_3$) δ 2.12-2.21 (m, 2H), 2.78 (t, 2H, J = 7.2 Hz), 3.39 (t, 2H, J = 6.6 Hz), 7.18-7.32 (m, 5H); ^{13}C-NMR (100 MHz, CDCl$_3$) δ 33.3, 34.1, 34.3, 126.3, 128.6, 128.7, 140.7; MS for $C_9H_{11}Br$: calcd 198.0, found 198.0.

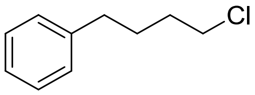

5Ga

(4-Chlorobutyl)benzene (5Ga, Table 1, entry 13) [9]**.** Isolated yield: 98%. ^{1}H-NMR (400 MHz, CDCl$_3$) δ 1.78-1.80 (m, 4H), 2.64 (t, 2H, J = 7.0 Hz), 3.54 (M, 2H), 7.17-7.20 (m, 3H), 7.26-7.30 (m, 2H); ^{13}C-NMR (100 MHz, CDCl$_3$) δ 28.8, 32.3, 35.3, 45.1, 126.1, 126.2, 128.6, 142.1; MS for $C_{10}H_{13}Cl$: calcd 168.1, found 168.1.

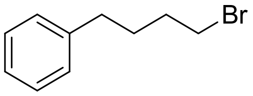

5Gb

(4-Bromobutyl)benzene (5Gb, Table 1, entry 14) [10]**.** Isolated yield: 92%. ^{1}H-NMR (400 MHz, CDCl$_3$) δ 1.75-1.81 (m, 2H), 1.86-1.91 (m, 2H), 2.64 (t, 2H, J = 7.5 Hz), 3.42 (t, 2H, J = 6.7 Hz), 7.17-7.30 (m, 5H); ^{13}C-NMR (100 MHz, CDCl$_3$) δ 30.0, 32.4, 33.8, 35.1, 126.1, 128.5, 128.5, 141.9; MS for $C_{10}H_{13}Br$: calcd 212.0, found 212.0.

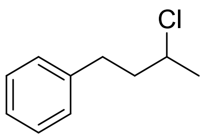

5Ha

(3-Chlorobutyl)benzene (5Ha, Table 1, entry 15 and 17, Table 4) [11]**.** Isolated yields: 95% for entry 15 and 92% for entry 17. ^{1}H-NMR (300 MHz, $CDCl_3$) δ 1.53 (d, 3H, *J* = 6.5 Hz), 1.98-2.05 (m, 2H), 2.72-2.86 (m, 2H), 3.96-4.03 (m, 1H), 7.20-7.32 (m, 5H); ^{13}C-NMR (100 MHz, $CDCl_3$) δ 25.6, 33.1, 42.1, 58.1, 126.3, 128.6, 128.7, 141.3; MS for $C_{10}H_{13}Cl$: calcd 168.1, found 168.1.

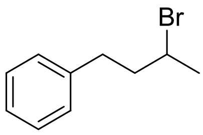

5Hb

(3-Bromobutyl)benzene (5Hb, Table 1, entry 16) [12]**.** Isolated yield: 91%. ^{1}H-NMR (400 MHz, $CDCl_3$) δ 1.73 (d, 3H, *J* = 6.6 Hz), 2.03-2.15 (m, 2H), 2.74-2.86 (m, 2H), 4.06-4.09 (m, 1H), 7.18-7.31 (m, 5H); ^{13}C-NMR (100 MHz, $CDCl_3$) δ 26.7, 34.1, 42.8, 51.1, 126.4, 128.6, 128.7, 141.1; MS for $C_{10}H_{13}Br$: calcd 212.0, found 212.0.

General procedure for aldehyde halogenation reactions using 16

To **16** (0.3 g, 0.3 mmol) was added chloroform (3 mL) in a round-bottom flask. After 10 min, oxalyl chloride or oxalyl bromide was added (0.3 mmol). The reaction mixture was magnetically stirred at room temperature. Upon cessation of gas evolution, **6** was added (0.1 mmol) and the reaction mixture was heated to reflux. After 72 h, the reaction mixture was cooled to room temperature and then filtered. The solid on funnel was washed with dichloromethane (3 × 5 mL). The filtrate was concentrated under reduced pressure to afford the crude product. The crude product was purified by flash silica gel column chromatography using 5% ethyl acetate in hexanes as the eluent.

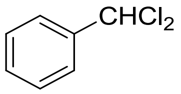

7Aa

(Dichloromethyl)benzene (7Aa, Table 2, entry 1) [13]. Isolated yield: 54%. ^{1}H-NMR (400 MHz, $CDCl_3$) δ 6.73 (s, 1H), 7.39-7.42 (m, 3H), 7.58-7.60 (m, 2H); ^{13}C-NMR (100 MHz, $CDCl_3$) δ 71.9, 126.2, 128.9, 130.0, 140.5; MS for $C_7H_6Cl^+$ ($C_7H_6Cl_2$): calc 125.0, found 125.0.

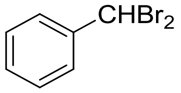

7Ab

(Dibromomethyl)benzene (7Ab, Table 2, entry 2) [14]. Isolated yield: 75%. ^{1}H-NMR (400 MHz, $CDCl_3$) δ 6.66 (s, 1H), 7.32-7.39 (m, 3H), 7.57 (d, 2H, *J* = 7.3 Hz); ^{13}C-NMR (100 MHz, $CDCl_3$) δ 41.2, 126.6, 128.7, 130.0, 142.0; MS for $C_7H_6Br^+$ ($C_7H_6Br_2$): calc 170.0, found 170.0.

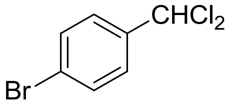

7Ba

1-Bromo-4-(dichloromethyl)benzene (7Ba, Table 2, entry 3) [13]. Isolated yield: 65%. ^{1}H-NMR (400 MHz, $CDCl_3$) δ 6.66 (s, 1H), 7.44 (d, 2H, *J* = 8.5 Hz), 7.54 (d, 2H, *J* = 8.5 Hz); ^{13}C-NMR (100 MHz, $CDCl_3$) δ 71.0, 124.2, 127.9, 132.1, 139.5; MS for $C_7H_5BrCl_2$: calc 237.9, found 237.9.

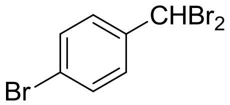

7Bb

1-Bromo-4-(dibromomethyl)benzene (7Bb, Table 2, entry 4) [15]. Isolated yield: 83%. ^{1}H-NMR (400 MHz, $CDCl_3$) δ 6.59 (s, 1H), 7.45 (d, 2H, *J* = 8.4 Hz), 7.51 (d, 1H, *J* = 8.4 Hz); ^{13}C-NMR (100 MHz, $CDCl_3$) δ 39.7,

124.0, 128.3, 132.0, 141.1; MS for $C_7H_4Br_2$ ($C_7H_5Br_3$): calc 247.9, found 247.1.

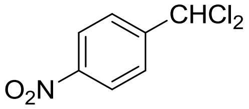

7Ca

1-(Dichloromethyl)-4-nitrobenzene (7Ca, Table 2, entry 5) [13]. Isolated yield: 64%. ^{1}H-NMR (400 MHz, $CDCl_3$) δ 6.77 (s, 1H), 7.76 (d, 2H, *J* = 8.7 Hz), 8.26 (d, 2H, *J* = 8.7 Hz). ^{13}C-NMR (100 MHz, $CDCl_3$) δ 69.9, 124.2, 127.5, 146.3, 148.6; MS for $C_7H_5ClNO_2^+$ ($C_7H_5Cl_2NO_2$): calc 170.0, found 170.0.

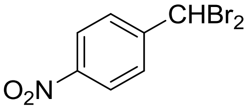

7Cb

1-(Dibromomethyl)-4-nitrobenzene (7Cb, Table 2, entry 6). Isolated yield: 89%. ^{1}H-NMR (400 MHz, $CDCl_3$) δ 6.67 (s, 1H), 7.75 (d, 2H, *J* = 8.7 Hz), 8.25 (d, 2H, *J* = 8.7 Hz); ^{13}C-NMR (100 MHz, $CDCl_3$) δ 38.3, 124.2, 127.9, 148.0; HRMS for $C_7H_4BrNO_2^+$ ($C_7H_5Br_2NO_2$): calc 213.9504, found 214.0910.

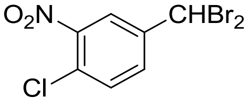

7Da

1-Chloro-4-(dibromomethyl)-2-nitrobenzene (7Da, Table 2, entry 7). Isolated yield: 61%. ^{1}H-NMR (300 MHz, $CDCl_3$) δ 6.72 (s, 1H), 7.63 (d, 1H, *J* = 8.4 Hz), 7.76 (dd, 1H, J_1 = 8.4 Hz, J_2 = 2.3 Hz), 8.1 (d, 1H, *J* = 2.3 Hz); ^{13}C-NMR (100 MHz, $CDCl_3$) δ 69.1, 123.6, 128.7, 130.8, 132.6, 138.2, 140.3; MS for $C_7H_4Cl_2NO_2^+$ ($C_7H_4Cl_3NO_2$): calc 203.9, found 203.9.

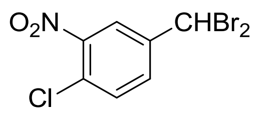

7Db

1-Chloro-4-(dibromomethyl)-2-nitrobenzene (7Db, Table 2, entry 8) [14]. Isolated yield: 93%. ^{1}H-NMR (400 MHz, $CDCl_3$) δ 6.63 (s, 1H), 7.59 (d, 1H, *J* = 8.4 Hz), 7.76 (d, 1H, *J* = 8.4 Hz), 8.09 (s, 1H); ^{13}C-NMR (100 MHz, $CDCl_3$) δ 36.9, 123.9, 128.4, 131.3, 132.6, 141.9, 147.6; MS for $C_7H_4BrClNO_2^+$ ($C_7H_4Br_2ClNO_2$): calc 247.9, found 248.1.

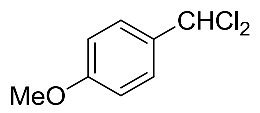

7Ea

1-(Dichloromethyl)-4-methoxybenzene (7Ea, Table 2, entry 9) [13]. Isolated yield: 94%. ^{1}H-NMR (400 MHz, $CDCl_3$) δ 3.83 (s, 3H), 6.71 (s, 1H), 6.92 (d, 2H, *J* = 8.7 Hz), 7.51 (d, 2H, *J* = 8.7Hz); ^{13}C-NMR (100 MHz, $CDCl_3$) δ 55.5, 71.9, 114.1, 127.7, 132.8, 160.7; MS for CH_8ClO^+ ($C_8H_8Cl_2O$): calc 155.0, found 155.0.

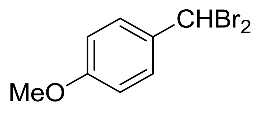

7Eb

1-(Dibromomethyl)-4-methoxybenzene (7Eb, Table 2, entry 10). NMR yield: 5%. ^{1}H-NMR (400 MHz, $CDCl_3$) δ 3.76 (s, 3H), 6.63 (s, 1H), 6.81 (d, 2H, *J* = 8.5 Hz), 7.46 (d, 2H, *J* = 8.5 Hz); ^{13}C-NMR (100 MHz, $CDCl_3$) δ 40.9, 55.4, 113.7, 121.2, 127.9, 160.4; MS for $C_8H_8BrO^+$ ($C_8H_8Br_2O$): calc 199.0, found 199.0

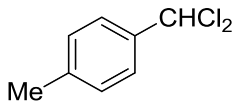

7Fa

1-(Dichloromethyl)-4-methylbenzene (7Fa, Table 2, entry 11) [13]. Isolated yield: 85%. ^{1}H-NMR (400 MHz, $CDCl_3$) δ 2.37 (s, 3H), 6.68 (s, 1H), 7.20 (d, 2H, *J* = 8.0 Hz), 7.45 (d, 2H, *J* = 8.0 Hz); ^{13}C-NMR (100 MHz, $CDCl_3$) δ 21.4, 72.0, 126.1, 129.5, 137.7, 140.2; MS for $C_8H_8Cl^+$ ($C_8H_8Cl_2$): calc 139.0, found 139.0.

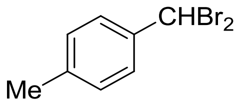

7Fb

1-(Dibromomethyl)-4-methylbenzene (7Fb, Table 2, entry 12). Isolated yield: 77%. ^{1}H-NMR (400 MHz, $CDCl_3$) δ 2.36 (s, 3H), 6.63 (s, 1H), 7.16 (d, 2H, *J* = 8.0 Hz), 7.45 (d, 2H, *J* = 8.0 Hz); ^{13}C-NMR (100 MHz, $CDCl_3$) δ 21.4, 41.3, 126.6, 129.4, 139.4, 140.2; MS for $C_8H_8Br^+$ ($C_8H_8Br_2$): calc 183.0, found 182.9.

General procedure for aziridine halogenation reactions using 16

To **16** (0.6 g, 0.6 mmol) was added dichloromethane (5 mL) in round-bottom flask. After 10 min, oxalyl chloride or oxalyl bromide was added (0.6 mmol). The reaction mixture was magnetically stirred at room temperature. Upon cessation of gas evolution, **8** was added (0.5 mmol), and the reaction mixture was heated to reflux. After the reaction was complete according to TLC analysis, the mixture was cooled to room temperature and filtered. The solid on funnel was washed with dichloromethane (3 × 10 mL), and the filtrate was concentrated to afford the desired product **9** in an essentially pure state based on ^{1}H and ^{13}C NMR spectroscopic analyses.

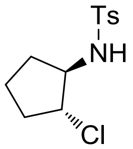

9Aa

***N*-(2-Chlorocyclopentyl)-4-methylbenzenesulfonamide (9Aa, Table 3, entry 1)** [16]**.** Isolated yield: 95%. ^{1}H-NMR (400 MHz, $CDCl_3$) δ1.41-1.43 (m, 1H), 1.72-1.85 (m, 3H), 2.11-2.18 (m, 2H), 2.44 (s, 3H), 3.57-3.59 (m, 1H), 4.06-4.10 (m, 1H), 5.28 (d, 1H, *J* = 5.9 Hz), 7.33 (d, 2H, *J* = 8.0 Hz), 7.79 (d, 2H, *J* = 8.0 Hz); ^{13}C-NMR (100 MHz, $CDCl_3$) δ 20.9, 21.8, 30.7, 33.6, 62.8, 63.8, 127.4, 130.0, 137.1, 144.0; MS for $C_{12}H_{16}ClNO_2S$: calcd 273.1, found 273.1.

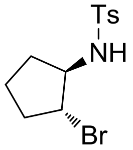

9Ab

***N*-(2-Bromocyclopentyl)-4-methylbenzenesulfonamide (9Ab, Table 3, entry 2)** [17]**.** Isolated yield: 93%. ^{1}H-NMR (400 MHz, $CDCl_3$) δ 1.26-1.42 (m, 1H), 1.63-1.82 (m, 2H), 1.93-1.96 (m, 1H), 2.16-2.28 (m, 2H), 2.45 (s, 3H), 3.65-3.80 (m, 1H), 4.10-4.11 (m, 1H), 5.25 (d, 1H, *J* = 4.2 Hz), 7.34 (d, 2H, *J* = 7.4 Hz), 7.80 (d, 2H, *J* = 7.4 Hz) ; ^{13}C-NMR (100 MHz, $CDCl_3$) δ 21.5, 21.7, 30.8, 34.2, 54.4, 63.1, 127.4, 130.0, 136.9, 143.9; MS for $C_{12}H_{16}BrNO_2S$: calcd 317.0, found 317.0.

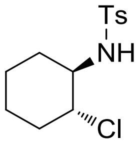

9Ba

***N*-(2-Chlorocyclohexyl)-4-methylbenzenesulfonamide (9Ba, Table 3, entry 3 and 5, Table 5)** [18]**.** Isolated yields: 89% for entry 3, and 95% for entry 5. ^{1}H-NMR (400 MHz, $CDCl_3$) δ 1.23-1.32 (m, 4H), 1.58-1.71 (m, 2H), 2.16-2.24 (m, 2H), 2.43 (s, 3H), 3.08-3.10 (m, 1H), 3.71 (dt, 1H, J_1 = 9.6 Hz, J_2 = 4.1 Hz), 4.98-5.10 (m, 1H), 7.31 (d, 2H, *J* = 8.2 Hz), 7.78 (d, 2H, *J* = 8.2 Hz); ^{13}C-NMR (100 MHz, $CDCl_3$) δ 21.4, 23.2, 24.0, 32.1, 34.7, 58.4, 61.9,

127.1, 129.5, 137.5, 143.2; MS for $C_{13}H_{16}ClNO_2S$: calcd 287.1, found 287.1.

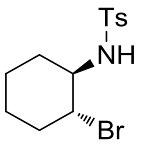

9Bb

***N*-(2-Bromocyclohexyl)-4-methylbenzenesulfonamide (9Bb, Table 3, entry 4)** [17]**.** Isolated yield: 92%. ^{1}H-NMR (300 MHz, CDCl$_3$) δ 1.25-1.44 (m, 3H), 1.65-1.82 (m, 3H), 2.27-2.32 (m, 2H), 2.43 (s, 3H), 3.14-3.17 (m, 1H), 3.84 (dt, 1H, J_1 = 9.6 Hz, J_2 = 5.7 Hz), 4.78-4.90 (m, 1H), 7.31 (d, 2H, J = 8.0 Hz), 7.78 (d, 2H, J = 8.3 Hz); ^{13}C-NMR (100 MHz, CDCl$_3$) δ 21.7, 23.6, 25.5, 33.0, 35.9, 55.2, 58.8, 127.5, 129.7, 137.3, 143.6; MS for $C_{13}H_{18}BrNO_2S$: calcd 331.0, found 331.0.

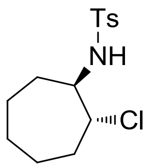

9Ca

***N*-(2-Chlorocycloheptyl)-4-methylbenzenesulfonamide (9Ca, Table 3, entry 6).** Isolated yield: 96%. ^{1}H-NMR (400 MHz, CDCl$_3$) δ 1.45-1.60 (m, 7H), 2.00-2.05 (m, 3H), 2.43 (s, 3H), 3.36-3.38 (m, 1H), 3.92-3.94 (m, 1H), 5.03-5.08 (m, 1H), 7.31 (d, 2H, J = 8.1 Hz), 7.78 (d, 1H, J = 8.1 Hz); ^{13}C-NMR (100 MHz, CDCl$_3$) δ 21.8, 22.8, 22.9, 27.4, 31.0, 34.2, 61.9, 65.7, 127.6, 129.8, 137.1, 143.8; HRMS for $C_{14}H_{20}ClNO_2S$: calcd 301.0903, found 301.0899.

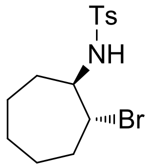

9Cb

***N*-(2-Bromocycloheptyl)-4-methylbenzenesulfonamide (9Cb, Table 3, entry 7).** Isolated yield: 98%. ^{1}H-NMR (400 MHz, $CDCl_3$) δ 1.48-1.61 (m, 7H), 2.03-2.15 (m, 3H), 2.43 (s, 3H), 3.48-3.50 (m, 1H), 4.04-4.09 (m, 1H), 4.92-4.99 (m, 1H), 7.31 (d, 2H, *J* = 8.0 Hz), 7.78 (d, 2H, *J* = 8.2 Hz); ^{13}C-NMR (100 MHz, $CDCl_3$) δ 21.8, 22.8, 24.0, 27.5, 31.3, 34.9, 58.8, 62.2, 127.6, 129.9, 137.0, 143.8; HRMS for $C_{14}H_{20}BrNO_2S$: calcd 345.0398, found 345.0393.

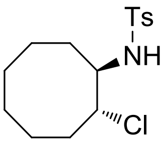

9Da

***N*-(2-Chlorocyclooctyl)-4-methylbenzenesulfonamide (9Da, Table 3, entry 8).** Isolated yield: 93%. ^{1}H-NMR (400 MHz, $CDCl_3$) δ 1.26-1.35 (m, 2H), 1.47-1.49 (m, 2H), 1.63-1.67 (m, 2H), 1.70-1.77 (m, 5H), 1.94-2.14 (m, 3H), 2.43 (s, 3H), 3.37-3.39 (m, 1H), 3.93-3.96 (m, 1H), 4.90-4.97 (m, 1H), 7.31 (d, 2H, *J* = 8.1 Hz), 7.77 (d, 2H, *J* = 8.1 Hz); ^{13}C-NMR (100 MHz, $CDCl_3$) δ 21.7, 24.0, 25.5, 25.7, 29.8, 31.3, 32.0, 60.7, 65.8, 127.6, 129.7, 136.7, 143.6; HRMS for $C_{15}H_{22}ClNO_2S$: calcd 315.1060, found 315.1053.

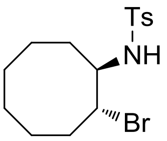

9Db

***N*-(2-Bromocyclooctyl)-4-methylbenzenesulfonamide (9Db, Table 3, entry 9)** [19]**.** Isolated yield: 92%. ^{1}H-NMR (400 MHz, $CDCl_3$) δ 1.26-1.33 (m, 2H), 1.48 (s, 2H), 1.67-1.74 (m, 5H), 1.99-2.11 (m, 2H), 2.11-2.43 (m,

1H), 2.48 (s, 3H), 3.50 (s, 1H), 4.05-4.08 (m, 1H), 4.95-5.03 (m, 1H), 7.31 (d, 2H, *J* = 7.8 Hz), 7.78 (d, 2H, *J* = 7.8 Hz); ^{13}C-NMR (100 MHz, $CDCl_3$) δ 21.7, 25.0, 25.4, 25.5, 25.8, 31.8, 32.2, 59.7, 61.0, 127.7, 129.6, 136.5, 143.6; MS for $C_{15}H_{22}BrNO_2S$: calcd 360.0, found 360.0.

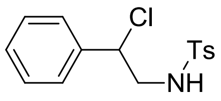

9Ea

***N*-(2-Chloro-2-phenylethyl)-4-methylbenzenesulfonamide (9Ea, Table 3, entry 10**) [20]. Isolated yield: 91%. ^{1}H-NMR (400 MHz, $CDCl_3$) δ 2.44 (s, 3H), 3.39-3.52 (m, 2H), 4.76 (t, 1H, *J* = 6.0 Hz), 4.87 (dd, 1H, J_1 = 8.2Hz, J_2 = 5.9Hz), 7.27-7.36 (m, 7H), 7.73 (d, 2H, *J* = 8.3Hz); ^{13}C-NMR (100 MHz, $CDCl_3$) δ 21.6, 50.4, 61.7, 127.1, 127.3, 129.0, 129.1, 130.0, 137.0, 137.9, 143.9; MS for $C_{15}H1_6ClNO_2S$: calcd 309.1, found 309.1.

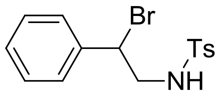

9Eb

***N*-(2-Bromo-2-phenylethyl)-4-methylbenzenesulfonamide (9Eb, Table 3, entry 11)** [21]. Isolated yield: 93%. ^{1}H-NMR (400 MHz, $CDCl_3$) δ 2.45 (s, 3H), 3.54-3.60 (m, 2H), 4.83 (t, 1H, *J* = 5.9 Hz), 4.90 (t, 1H, *J* = 7.0 Hz), 7.27-7.33 (m, 7H), 7.73 (d, 2H, *J* = 8.1 Hz); ^{13}C-NMR (100 MHz, $CDCl_3$) δ 21.7, 50.2, 52.7, 127.2, 127.7, 129.2, 129.3, 130.0, 137.1, 138.3, 144.0; Ms for $C_{15}H_{16}BrNO_2S$: calcd 353.0, found 353.0.

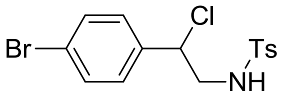

9Fa

***N*-(2-(4-Bromophenyl)-2-chloroethyl)-4-methylbenzenesulfonamide (9Fa, Table 3, entry 12)** [22]. Isolated yield: 93%. ^{1}H-NMR (400 MHz, $CDCl_3$) δ 2.44 (s, 3H), 3.39-3.44 (m, 2H), 4.85 (t, 1H, *J* = 7.7 Hz), 4.92 (t, 1H, *J* =

6.5 Hz), 7.16 (d, 2H, J = 8.3 Hz), 7.31 (d, 2H, J = 8.3 Hz), 7.46 (d, 2H, J = 8.3 Hz), 7.70 (d, 2H, J = 8.3 Hz); ^{13}C-NMR (100 MHz, $CDCl_3$) δ 21.7, 50.4, 61.0, 123.3, 127.1, 129.0, 130.0, 132.2, 136.9, 137.0, 144.1; MS for $C_{15}H_{15}BrClNO_2S$: calcd 387.0, found 387.0.

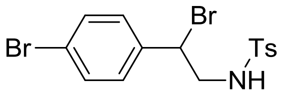

9Fb

***N*-(2-Bromo-2-(4-bromophenyl)ethyl)-4-methylbenzenesulfonamide (9Fb**, **Table 3**, **entry 13**). Isolated yield: 95%. ^{1}H-NMR (400 MHz, $CDCl_3$) δ 2.45 (s, 3H), 4.88 (t, 2H, J = 7.3 Hz), 7.16 (d, 2H, J = 8.3 Hz), 7.32 (d, 2H, J = 8.3 Hz), 7.44 (d, 2H, J = 8.3 Hz), 7.70 (d, 2H, J = 8.3 Hz); ^{13}C-NMR (100 MHz, $CDCl_3$) δ 21.7, 50.1, 51.6, 123.3, 127.1, 129.4, 130.0, 132.3, 137.0, 137.4, 144.1; HRMS for $C_{15}H_{15}Br_2NO_2S$: calcd 430.9190, found 430.9189.

General procedure for epoxide halogenation reactions using 18

To **18** (1.3 g, 1.2 mmol) was added chloroform (10 mL) in a round-bottom flask. After 10 min, oxalyl chloride or oxalyl bromide was added (1.1 mmol). The reaction mixture was magnetically stirred at room temperature. Upon cessation of gas evolution, **10** was added (0.5 mmol) and the reaction mixture was heated to reflux. After the reaction was completed as monitored by TLC, the mixture was cooled to room temperature and filtered. The solid on funnel was washed with dichloromethane (3 × 10 mL). The solvent of filtrate was removed to afford the desired product **11** in an essentially pure state based on ^{1}H and ^{13}C NMR spectroscopic analyses.

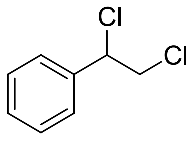

11Aa

(1,2-Dichloroethyl)benzene (11Aa, Table 6, entry 1) [23]**.** Isolated yield: 95%. ^{1}H-NMR (400 MHz, $CDCl_3$) δ 3.90-4.02 (m, 2H), 5.01 (t, 1H, *J* = 7.3 Hz), 7.35-7.42 (m, 5H); ^{13}C-NMR (100 MHz, $CDCl_3$) δ 48.5, 61.9, 127.5, 129.0, 129.3, 138.1; MS for $C_8H_8Cl_2$: calcd 174.0, found 174.0.

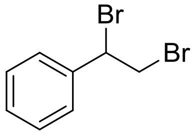

11Ab

(1,2-Dibromoethyl)benzene (11Ab, Table 6, entry 2) [24]**.** Isolated yield: 93%. ^{1}H-NMR (400 MHz, $CDCl_3$) δ 3.40-4.1 (m, 2H), 5.11-5.15 (dd, 1H, J_1 = 10.6 Hz, J_2 = 5.5 Hz), 7.24-7.32 (m, 5H); ^{13}C-NMR (100 MHz, $CDCl_3$) δ 35.1, 51.0, 127.7, 128.9, 129.3, 138.7; MS for $C_8H_8Br_2$: calcd 261.9, found 261.9.

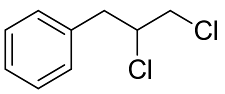

11Ba

(2,3-Dichloropropyl)benzene (11Ba, Table 6, entry 3) [23]**.** Isolated yield: 89%. ^{1}H-NMR (400 MHz, $CDCl_3$) δ 3.12 (dd, 1H, J_1 = 14.2 Hz, J_2 = 7.3 Hz), 3.37 (dd, 1H, J_1 = 14.2 Hz, J_2 = 5.7 Hz), 3.69-3.81 (m, 2H), 4.29-4.34 (m, 1H), 7.30-7.42 (m, 5H); ^{13}C-NMR (100 MHz, $CDCl_3$) δ 41.1, 47.6, 61.1, 127.3, 128.7, 129.7, 136.4; MS for $C_9H_{10}Cl_2$: calcd 188.0, found 188.0.

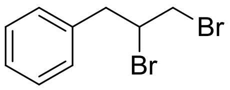

11Bb

(2,3-Dibromopropyl)benzene (11Bb, Table 6, entry 4) [25]**.** Isolated yield: 92%. ^{1}H-NMR (400 MHz, $CDCl_3$) δ 3.12 (dd, 1H, J_1 = 14.5 Hz, J_2 = 7.8 Hz), 3.38 (dd, 1H, J_1 = 14.5 Hz, J_2 = 4.8 Hz), 3.61 (dd, 1H, J_1 = 10.4 Hz, J_2 = 9.0 Hz), 3.81 (dd, 1H, J_1 = 10.5 Hz, J_2 = 4.2 Hz), 4.32-4.35 (m, 1H), 7.22-7.35 (m, 5H); ^{13}C-NMR (100 MHz, $CDCl_3$) δ 36.2, 42.1, 52.5, 127.3, 128.6, 129.6, 136.9; MS for $C_9H_{10}Br_2$: calcd 275.9, found 275.9.

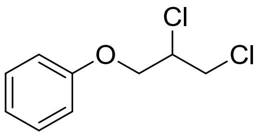

11Ca

(2,3-Dichloropropoxy)benzene (11Ca, Table 6, entry 5) [26]**.** Isolated yield: 95%. ^{1}H-NMR (400 MHz, $CDCl_3$) δ 3.88-3.99 (m, 2H), 4.27 (d, 2H, J = 6.0 Hz), 4.34-4.39 (m, 1H), 6.93 (d, 2H, J = 7.9 Hz), 6.94-7.01 (m, 1H), 7.28-7.32 (m, 2H); ^{13}C-NMR (100 MHz, $CDCl_3$) δ 45.2, 57.5, 68.3, 114.9, 121.8, 129.8, 158.1; MS for $C_9H_{10}Cl_2O$: calcd 204.0, found 204.0.

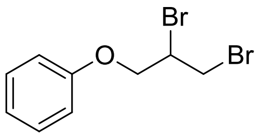

11Cb

(2,3-Dibromopropoxy)benzene (11Cb, Table 6, entry 6) [27]**.** Isolated yield: 96%. ^{1}H-NMR (400 MHz, $CDCl_3$) δ 3.88-3.97 (m, 2H), 4.35-4.40 (m, 2H), 4.42-4.46 (m, 1H), 6.95 (d, 2H, J = 8.2 Hz), 7.02 (t, 1H, J = 7.4 Hz), 7.29-7.34 (m, 2H); ^{13}C-NMR (100 MHz, $CDCl_3$) δ 32.9, 47.9, 69.1, 115.0, 121.8, 129.7, 158.1; MS for $C_9H_{10}Br_2O$: calcd 291.9, found 291.9.

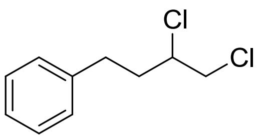

11Da

(3,4-Dichlorobutyl)benzene (11Da, Table 6, entry 7). Isolated yield: 98%. ^{1}H-NMR (400 MHz, $CDCl_3$) δ 2.01-2.05 (m, 1H), 2.28-2.33 (m, 1H), 2.74-2.80 (m, 1H), 2.88-2.92 (m, 1H), 3.66 (dd, 1H, J_1 = 11.3 Hz, J_2 = 7.4 Hz), 3.77 (dd, 1H, J_1 = 11.3 Hz, J_2 = 5.1 Hz), 3.98-4.00 (m, 1H), 7.20-7.22 (m, 3H), 7.28-7.32 (m, 2H); ^{13}C-NMR (100 MHz, $CDCl_3$) δ 32.1, 36.8, 48.4, 60.3, 126.4, 128.6, 128.7, 140.5; HRMS for $C_{10}H_{12}Cl_2$: calcd 202.0316, found .202.0321.

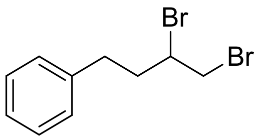

11Db

(3,4-Dibromobutyl)benzene (11Db, Table 6, entry 8). Isolated yield: 92%. ^{1}H-NMR (400 MHz, $CDCl_3$) δ 2.08-2.11 (m, 1H), 2.46-2.50 (m, 1H), 2.74-2.79 (m, 1H), 2.91-2.94 (m, 1H), 3.65 (t, 1H, J = 10.2 Hz), 3.87 (dd, 1H, J_1 = 10.3 Hz， J_2 = 4.4 Hz), 4.09-4.13 (m, 1H), 7.20-7.24 (m, 3H), 7.30-7.33 (m, 2H); ^{13}C-NMR (100 MHz, $CDCl_3$) δ 33.1, 36.4, 37.8, 52.2, 126.4, 128.7, 128.7, 140.4; HRMS for $C_{10}H_{12}Br_2$: calcd 289.9306, found 289.9300.

References

1. Leung P. S.-W.;Teng, Y.; Toy, P. H. *Synlett* **2010**, 1997-2001.

2. Teng Y.; Toy, P. H. *Synlett* **2011**, 551-554.

3. Klimesova, V.; Koci, J.; Waisser, K.; Kaustova, J. *Eur. J. Med. Chem.* **2009**, *45*, 2286-2293.

4. Mirjafari, A.; Mohammadpoor-Baltork, I.; Moghadam, M.; Tangestaninejad, S.; Mirkhani, V.; Khosropour, A. R. *Tetrahedron Lett.* **2010**, *51*, 3274-3276.

5. AIST: Integrated Spectral Database System of Organic Compounds. (Data were obtained from the National Institute of Advanced Industrial Science and Technology (Japan))

6. Tsoi, Y. T.; Zhou, Z.; Yu, W. Y. *Org. Lett.* **2011**, *13*, 5370-5373.

7. Parham, W. E.; Bradsher, C. K.; Reames, D. *J. Org. Chem.* **1981**, *46*, 4806-4808.

8. van Kalkeren, H. A.; Leenders, S. H. M.; Hommersom, C. R. A.; Rutjes, F. P. J. T.; van Delft, F. L. *Chem. Eur. J.* **2011**, *17*, 11290-11295.

9. Yang C. T.; Zhang, Z. Q.; Liu, Y. C.; Liu, L. *Angew. Chem. Int. Ed.* **2011**, *50*, 3904-3907.

10. Shahid, I.; Patel, C. H.; Dhanani, S.; Owen, C. P.; Ahmed, S. *J. Steroid Biochem. Mol. Biol.* **2008**, *110*, 18-29.

11. Gaspar, B.; Carreira, E. *Angew. Chem. Int. Ed.* **2008**, *47*, 5758-5760.

12. Someya, H.; Yorimitsu, H.; Oshima, K. *Tetrahedron* **2010**, *66*, 5993-5999.

13. An, J.; Tang, X.; Moore, J.; Lewis, W.; Denton, R. *Tetrahedron* **2013**, *69*, 8769-8776.

14. Hoffmann, R. W.; Bovicelli, P. *Synthesis* **1990**, 657-659.

15. Pingali, S. R. K.; Upadhyay, S. K.; Jursic, B. S. *Green Chem.* **2011**, *13*, 928-933.

16. Ghorai, M.; Kumar, A.; Tiwari, D. *J. Org. Chem.* **2009**, *75*, 137-151.

17. Wang, Z.; Zhang, Y.; Fu, H.; Jiang, Y.; Zhao, Y. *Synlett* **2008**, 2667-2670.

18. Minakata, S.; Yoneda, Y.; Oderaotoshi, Y.; Komatsu, M. *Org. Lett.* **2006**, *8*, 967-969.

19. Wei, J. F.; Chen, Z. G.; Wei, L.; Zhang, L.H.; Wang, M. Z.; Shi, X. Y.; Li, R. T. *Org. Lett.* **2009**, *11*, 4616-4219.

20. Minakata, S.; Yoneda, Y.; Oderaotoshi, Y.; Komatsu, M. *Org. Lett.* **2006**, *8*, 967-969.

21. Das, B.; Krishnaiah, M.; Venkateswarlu, K. *Tetrahedron Lett.* **2006**, *47*, 4457-4460.

22. Ghorai, M. K.; Kumar, A.; Tiwari, D. P. *J. Org. Chem.* **2010**, *75*, 137-151.

23. Denton, R. M.; Tang, X. P.; Przeslak, A. *Org. Lett.* **2010**, *12*, 4678-4681.

24. Zhang, J. L.; Wang, J.; Qiu, Z. B.; Wang, Y. *Tetrahedron* ***2011***, *67*, 6859-6867.

25. Kikushima, K.; Moriuchi, T.; Hirao, T. *Tetrahedron* **2010**, *66*, 6906-6911.

26. Ho, M. L.; Flynn, A. B.; Ogilvie, W. W. *J. Org. Chem.* **2007**, *72*, 977-983.

27. Braddock, D. C.; Cansell, G.; Hermitage, S. A. *Synlett* **2004**, 461-464.

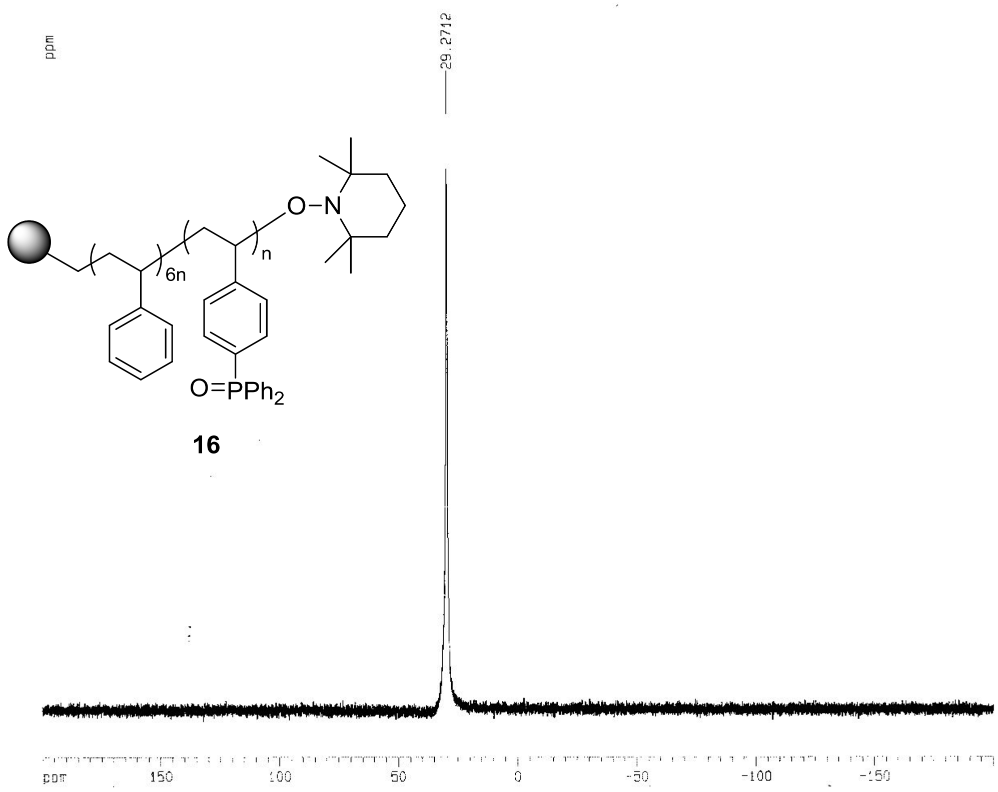
29.2712
O=PPh2
16
ppm 150 100 50 0 -50 -100 -150

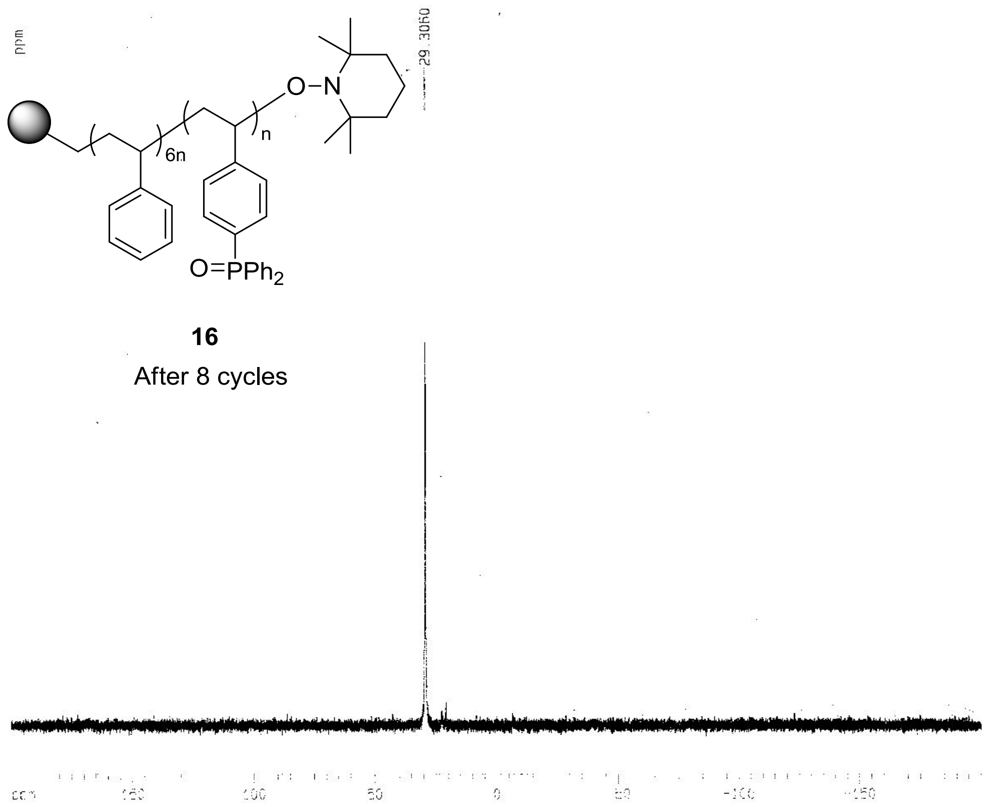
29.3060
O=PPh2
16
After 8 cycles

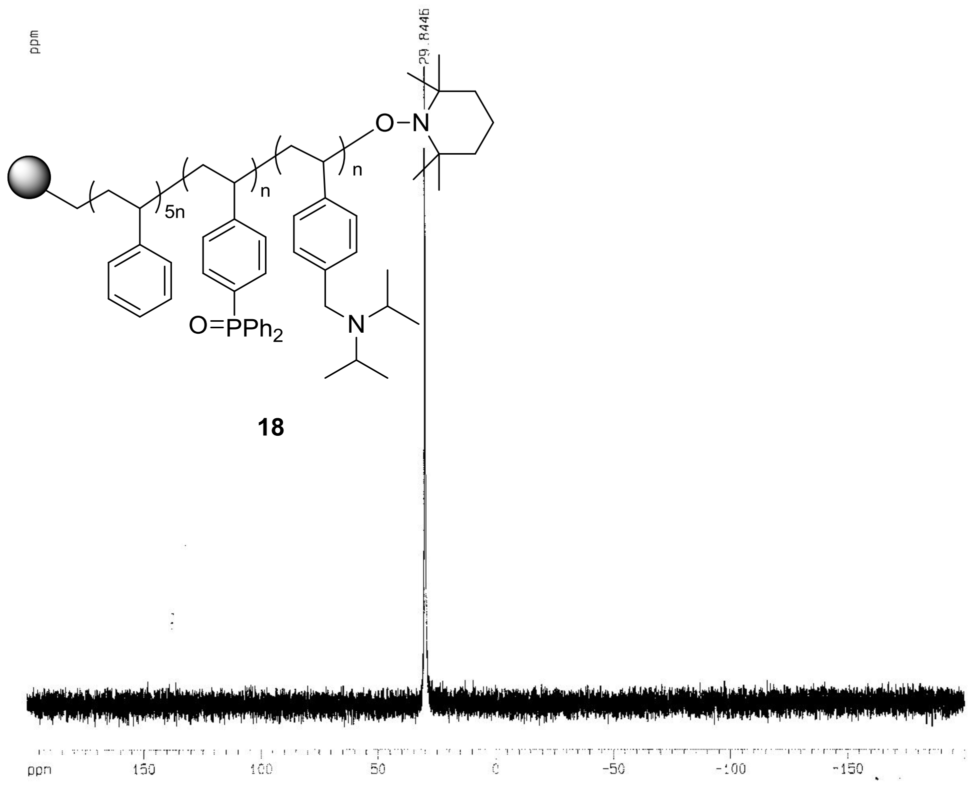

ppm
29.8446
O–N
5n
n
n
O=PPh2
N
18
ppm
150
100
50
0
-50
-100
-150

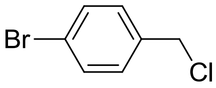

5Aa

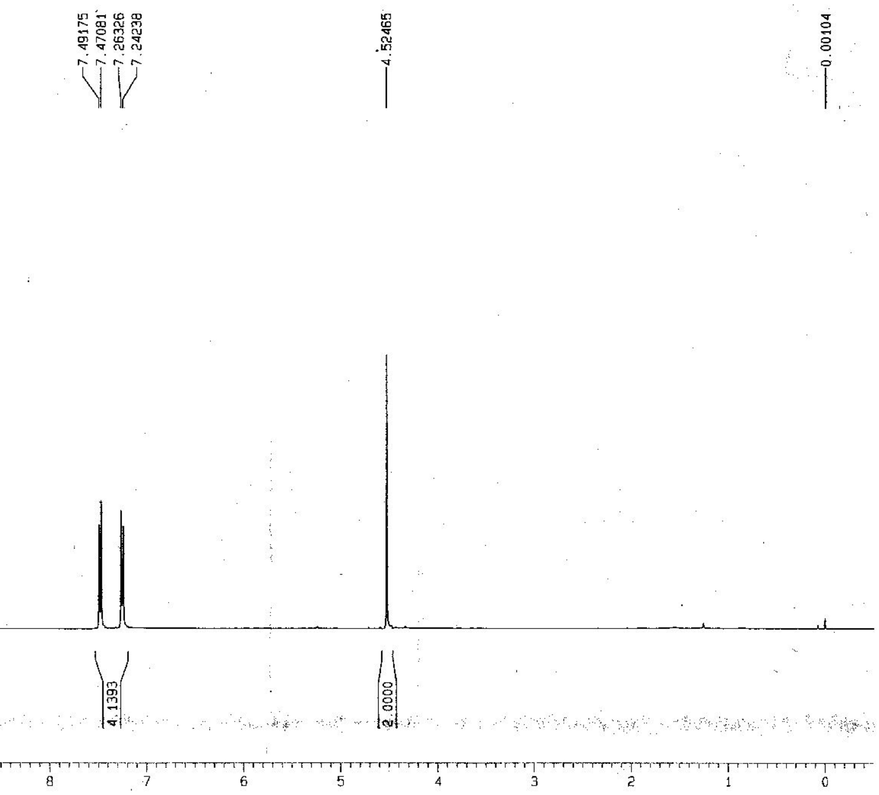
ppm
7.49175
7.47081
7.26326
7.24238
4.52465
0.00104
Integral
4.1393
2.0000
ppm 10 9 8 7 6 5 4 3 2 1 0

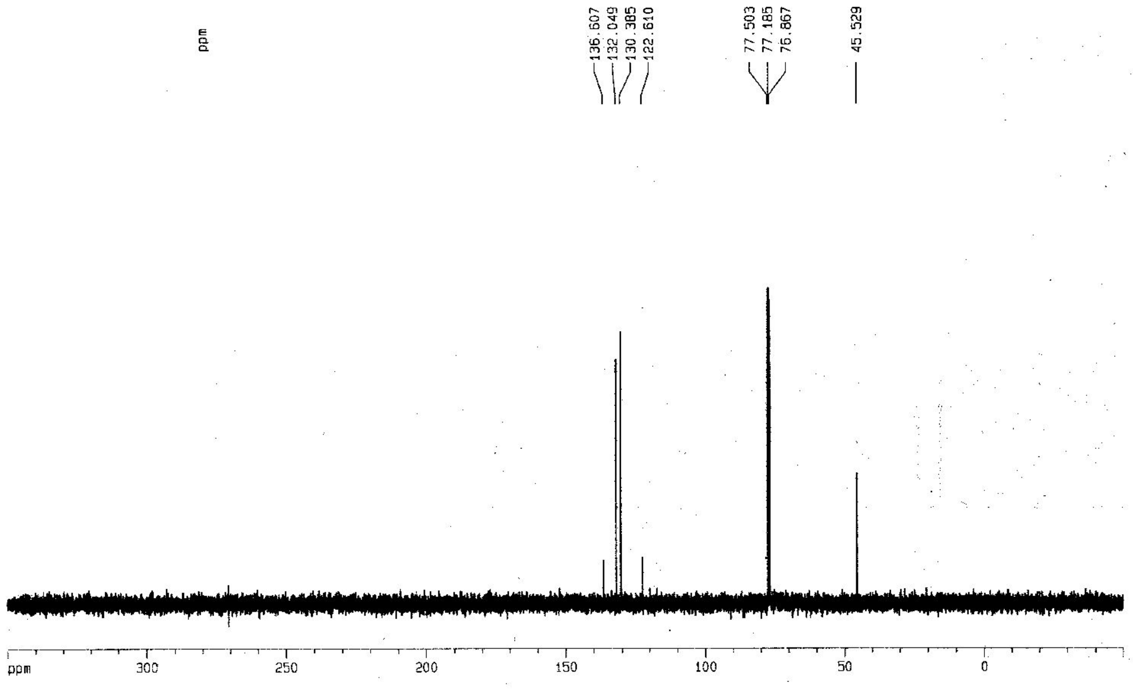
ppm
136.607
132.049
130.385
122.610
77.503
77.185
76.867
45.529
ppm 300 250 200 150 100 50 0

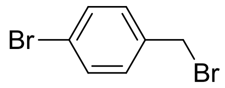

5Ab

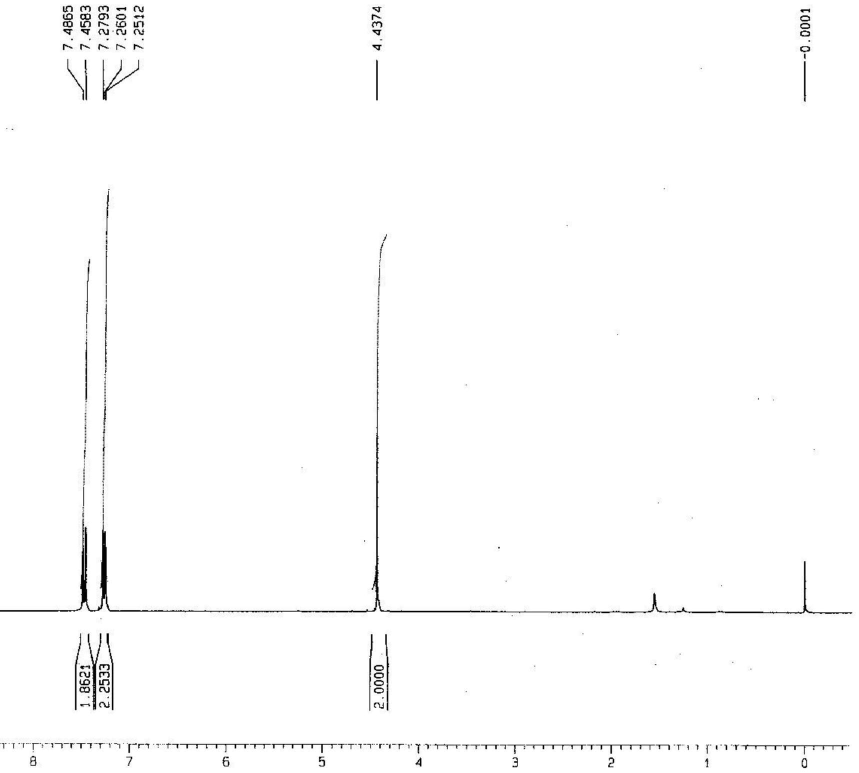

ppm
7.4865
7.4583
7.2793
7.2601
7.2512
4.4374
-0.0001
Integral
1.8621
2.2533
2.0000
ppm
10
9
8
7
6
5
4
3
2
1
0

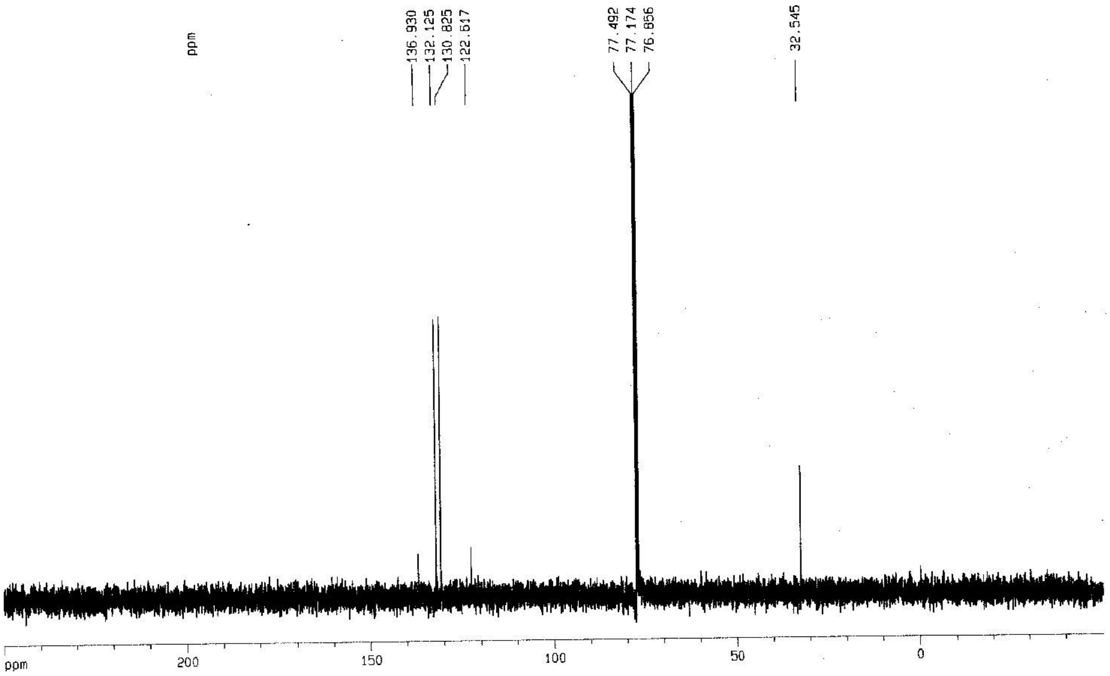

ppm
136.930
132.125
130.825
122.617
77.492
77.174
76.856
32.545
ppm
200
150
100
50
0

5Ba

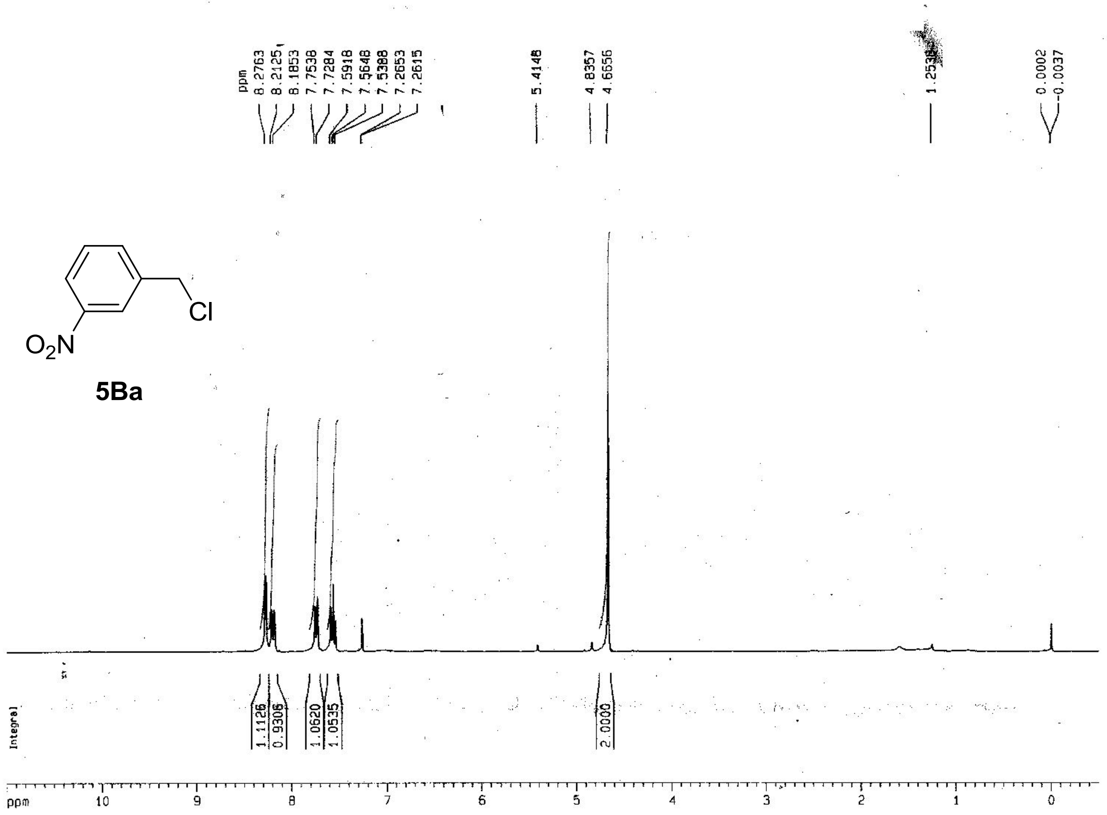
Cl
O_2N
ppm
8.2763
8.2125
8.1853
7.7538
7.7284
7.5918
7.5648
7.5388
7.2653
7.2615
5.4148
4.8357
4.6656
1.253[illegible]
0.0002
-0.0037
Integral
1.1126
0.9306
1.0620
1.0535
2.0000
ppm 10 9 8 7 6 5 4 3 2 1 0

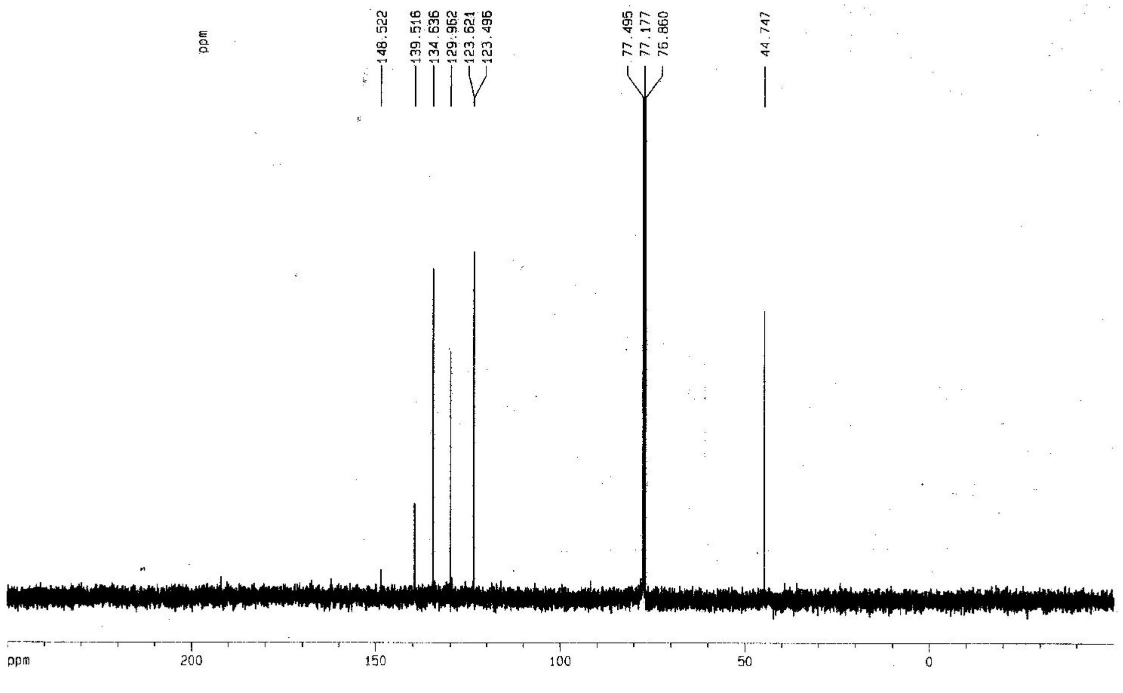
ppm
148.522
139.516
134.636
129.962
123.621
123.496
77.495
77.177
76.860
44.747
ppm 200 150 100 50 0

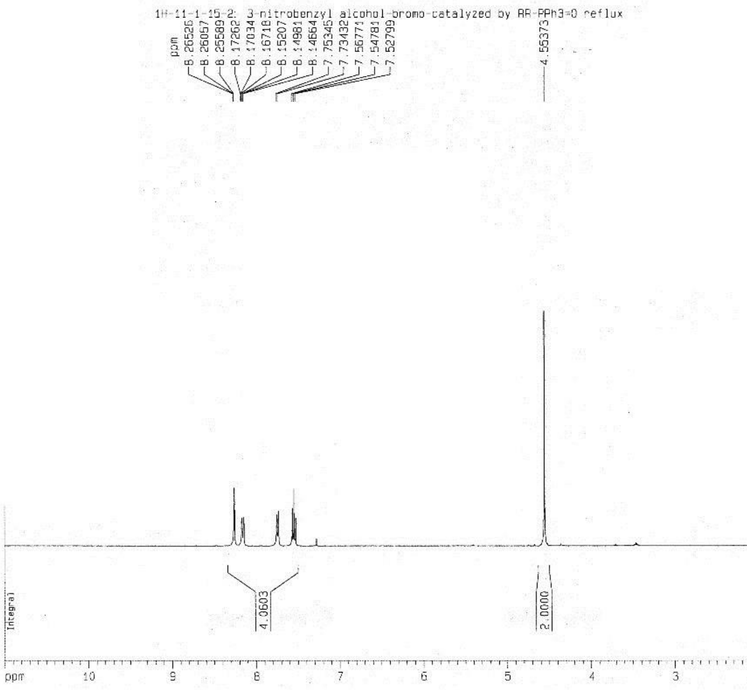
1H-11-1-15-2? 3-nitrobenzyl alcohol-bromo-catalyzed by RR-PPh3=O reflux
ppm
8.26526
8.26057
8.25589
8.17262
8.17034
8.16718
8.15207
8.14981
8.14664
7.75345
7.73432
7.56771
7.54781
7.52799
4.55373
Integral
4.0603
2.0000
ppm 10 9 8 7 6 5 4 3 2 1 0

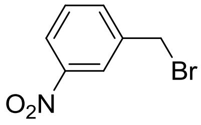
Br
O2N

5Bb

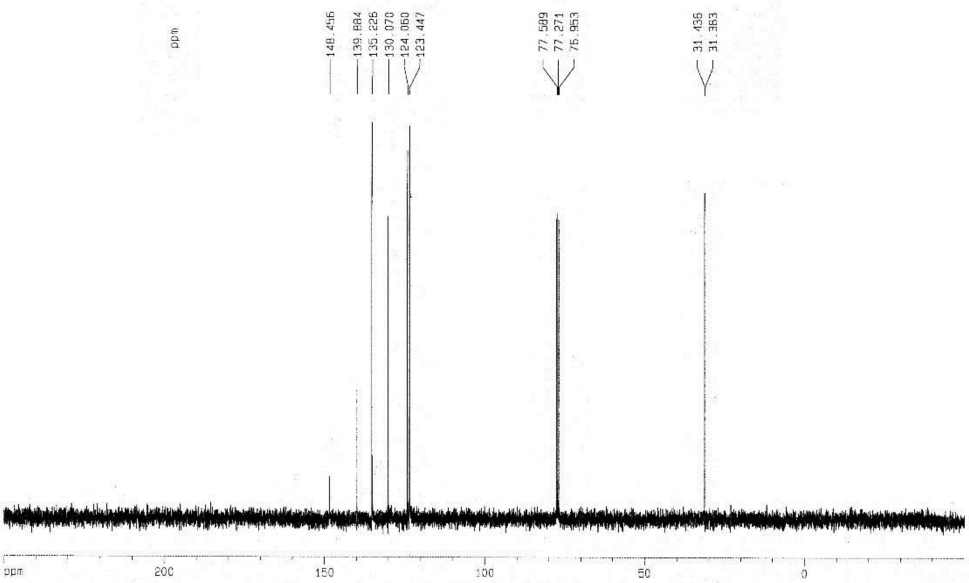
ppm
148.456
139.884
135.226
130.070
124.060
123.447
77.589
77.271
76.953
31.436
31.363
ppm 200 150 100 50 0

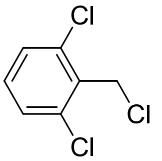
Cl
Cl
Cl

5Ca

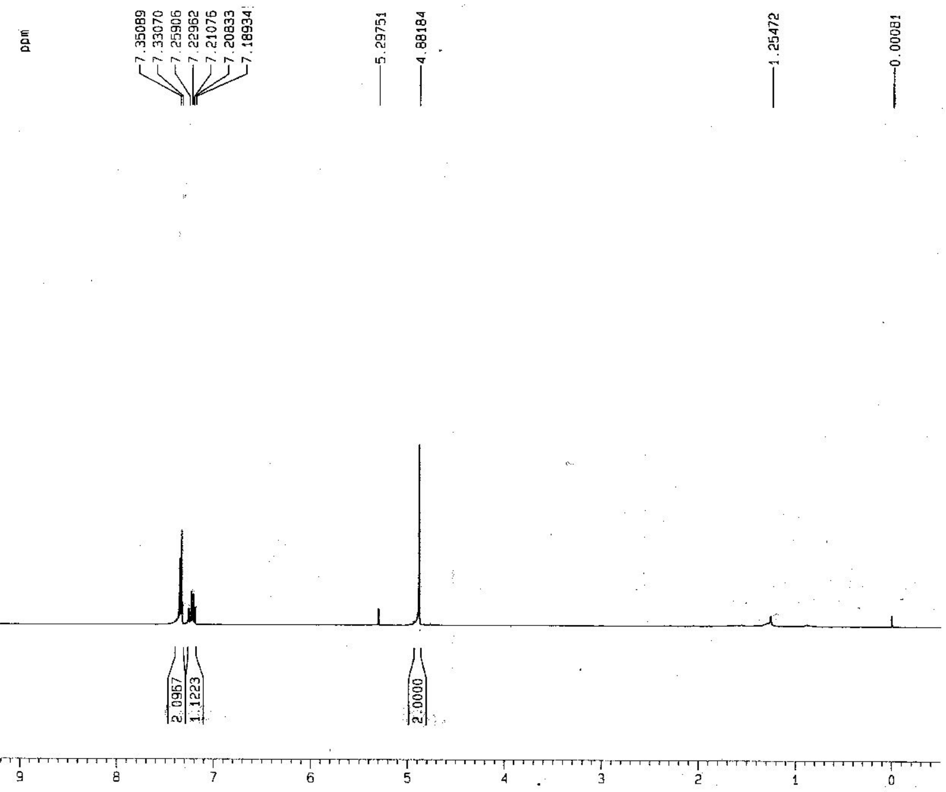
ppm
7.35089
7.33070
7.25906
7.22962
7.21076
7.20833
7.18934
5.29751
4.88184
1.25472
0.00081
Integral
2.0967
1.1223
2.0000
ppm
10
9
8
7
6
5
4
3
2
1
0

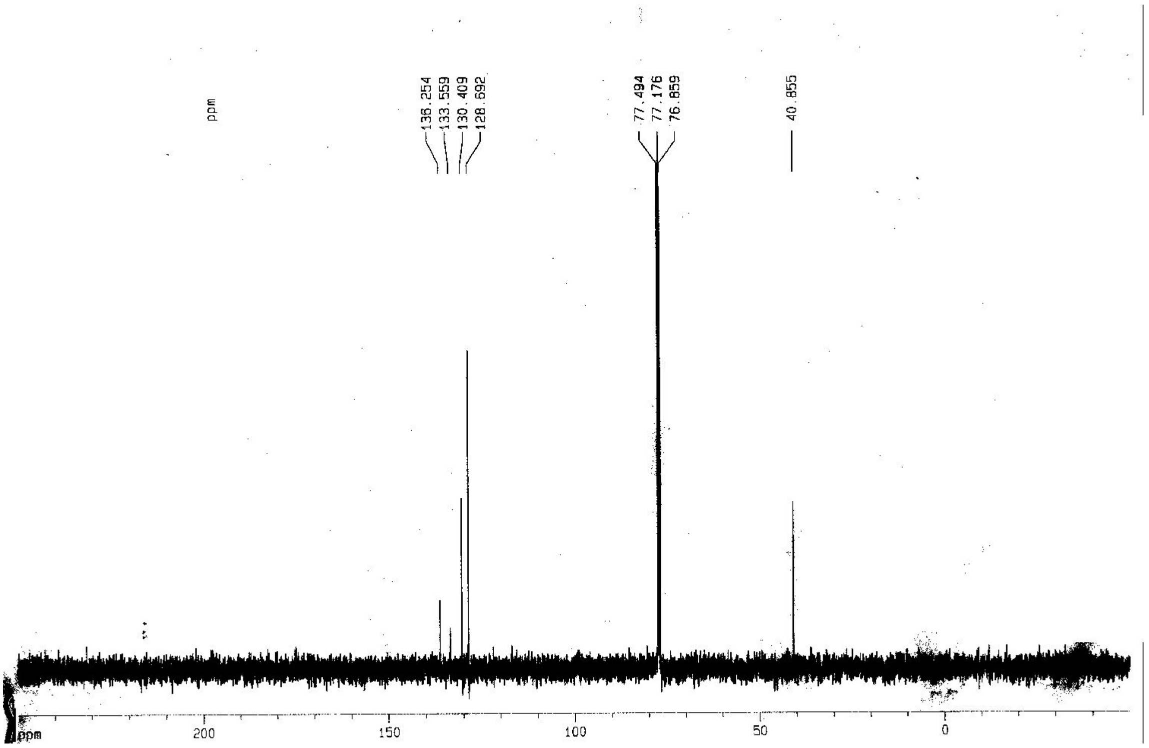
ppm
136.254
133.559
130.409
128.692
77.494
77.176
76.859
40.855
ppm
200
150
100
50
0

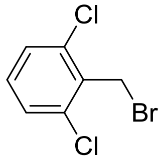
Cl
Br
Cl

5Cb

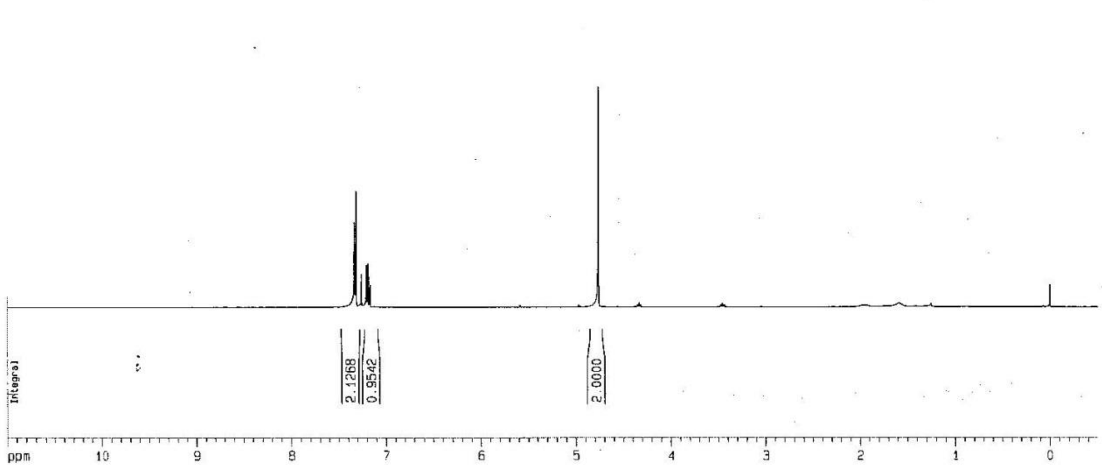
ppm
7.3392
7.3188
7.2599
7.2080
7.1891
7.1865
7.1677
4.7544
0.0001
Integral
2.1268
0.9542
2.0000
ppm 10 9 8 7 6 5 4 3 2 1 0

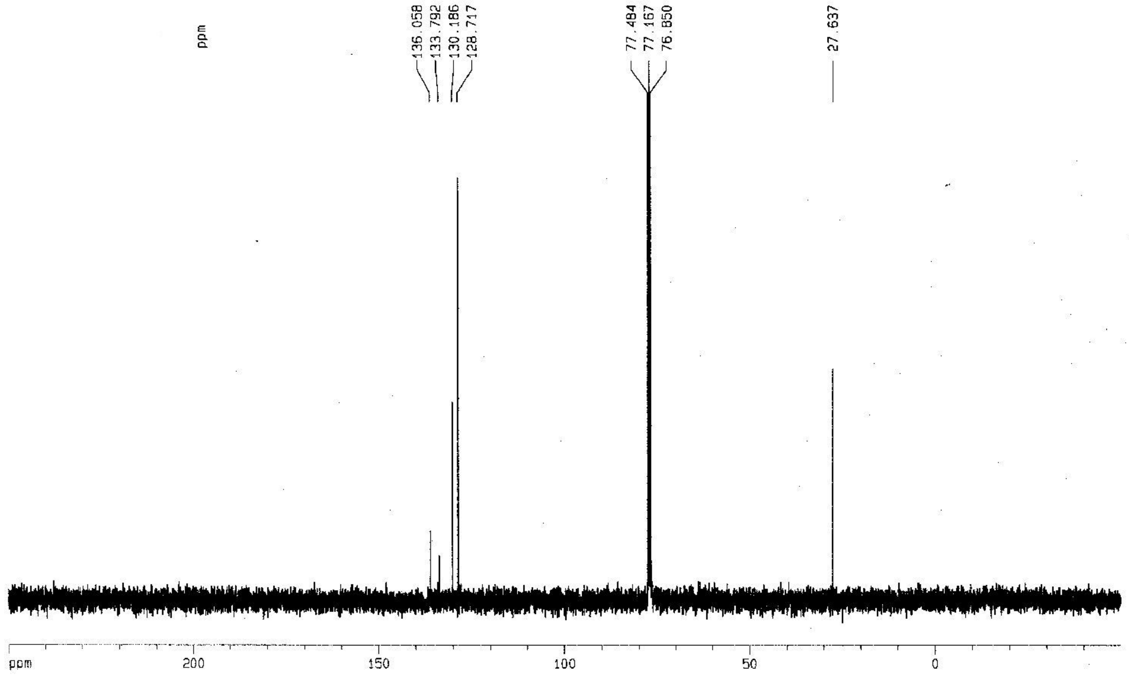
ppm
136.058
133.792
130.186
128.717
77.484
77.167
76.850
27.637
ppm 200 150 100 50 0

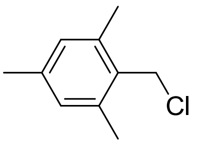
Cl

5Da

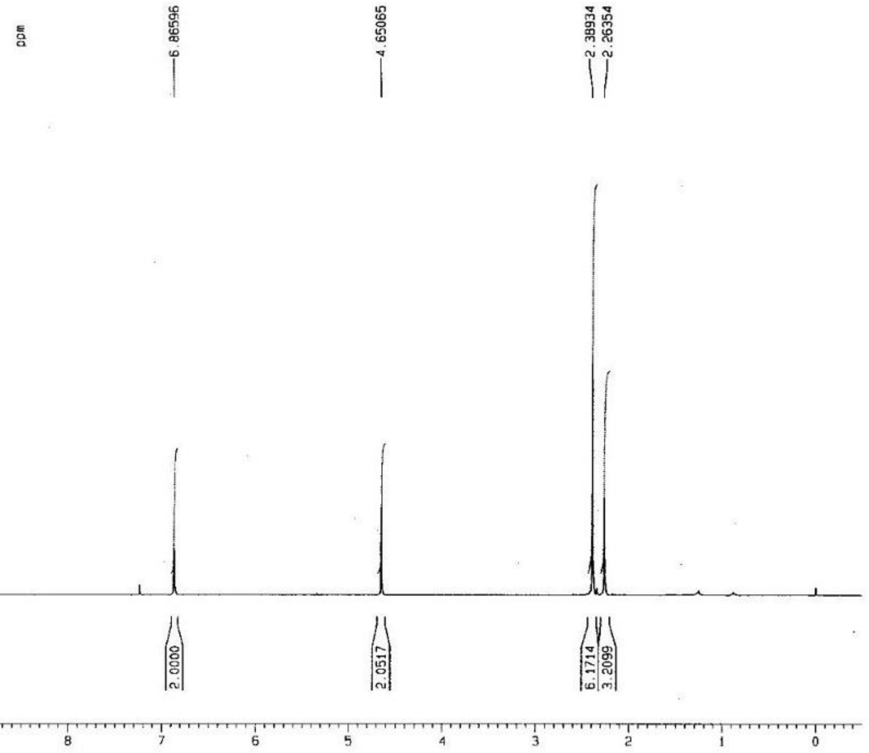
ppm
6.86596
4.65065
2.38934
2.26354
Integral
2.0000
2.0517
6.1714
3.2099
ppm 10 9 8 7 6 5 4 3 2 1 0

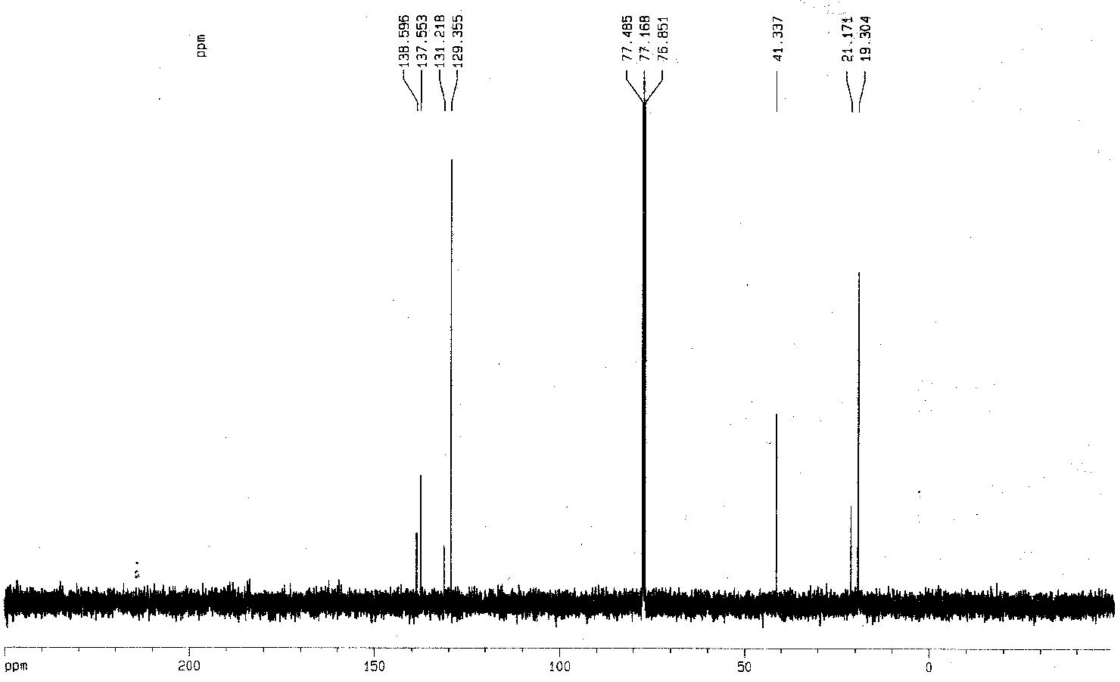
ppm
138.596
137.553
131.218
129.355
77.485
77.168
76.851
41.337
21.171
19.304
ppm 200 150 100 50 0

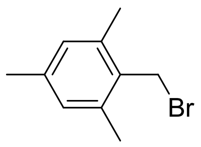
Br

5Db

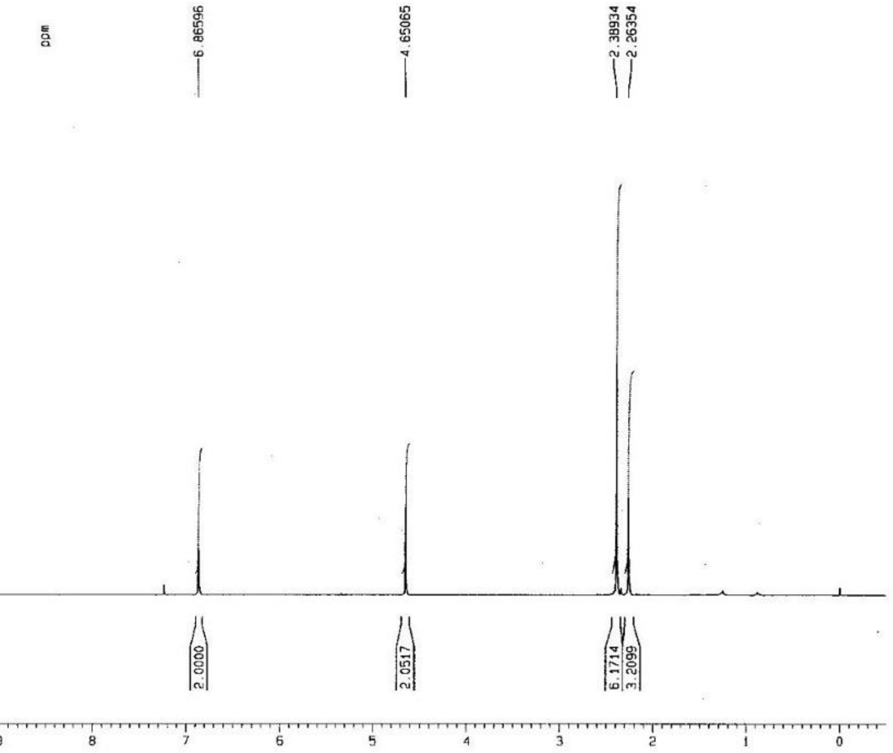
ppm
6.86596
4.65065
2.38934
2.26354
Integral
2.0000
2.0517
6.1714
3.2099
ppm 10 9 8 7 6 5 4 3 2 1 0

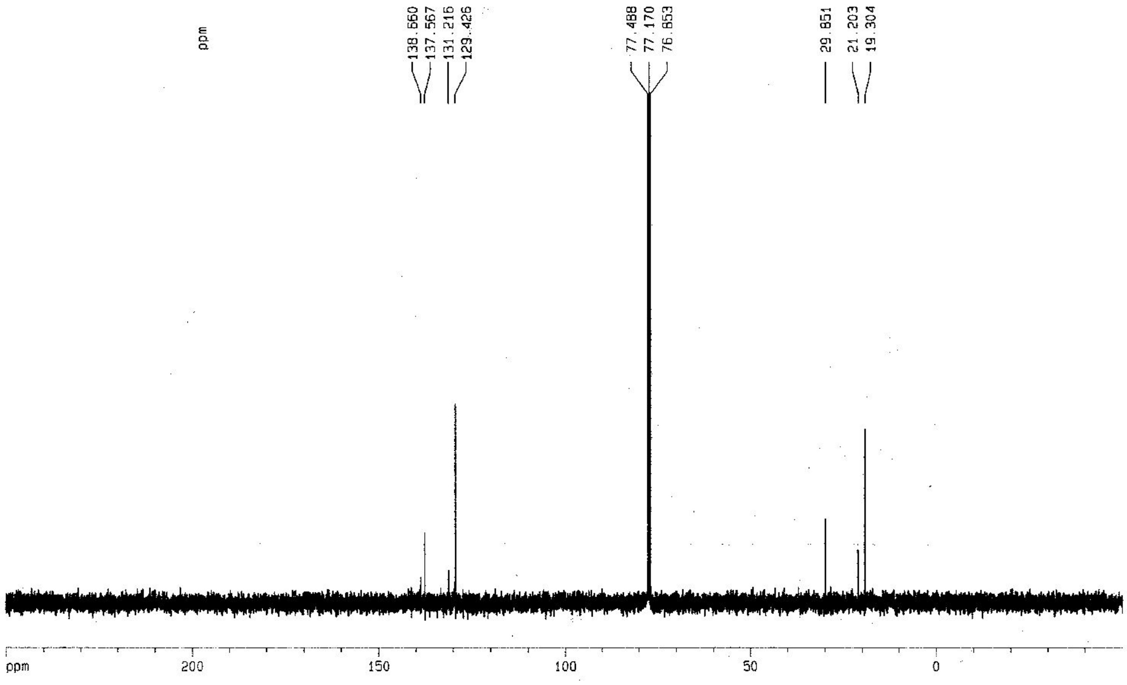
ppm
138.560
137.567
131.216
129.426
77.488
77.170
76.853
29.851
21.203
19.304
ppm 200 150 100 50 0

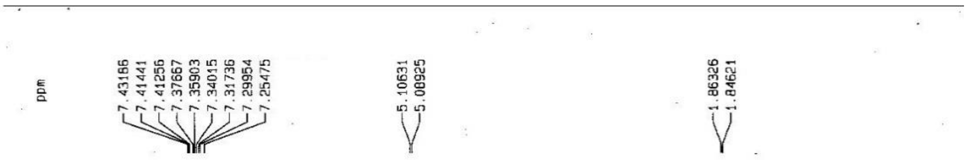

ppm
7.43166
7.41441
7.41256
7.37667
7.35903
7.34015
7.31736
7.29954
7.25475
5.10631
5.08925
1.86326
1.84621

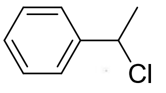

Cl

5Ea

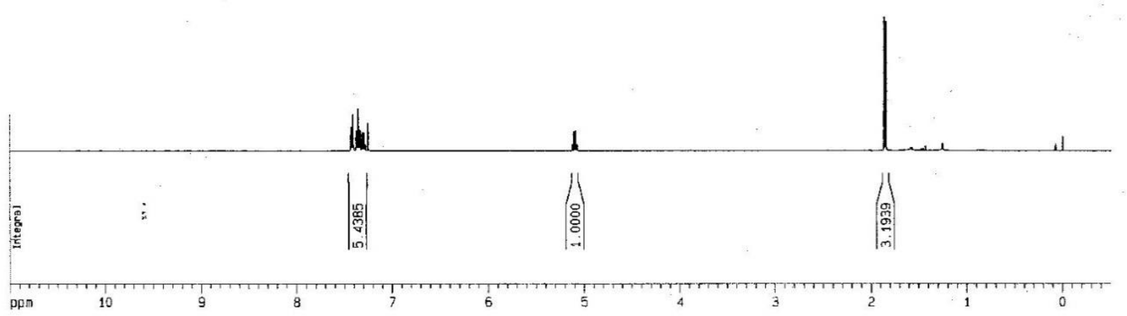

Integral
5.4385
1.0000
3.1939
ppm
10
9
8
7
6
5
4
3
2
1
0

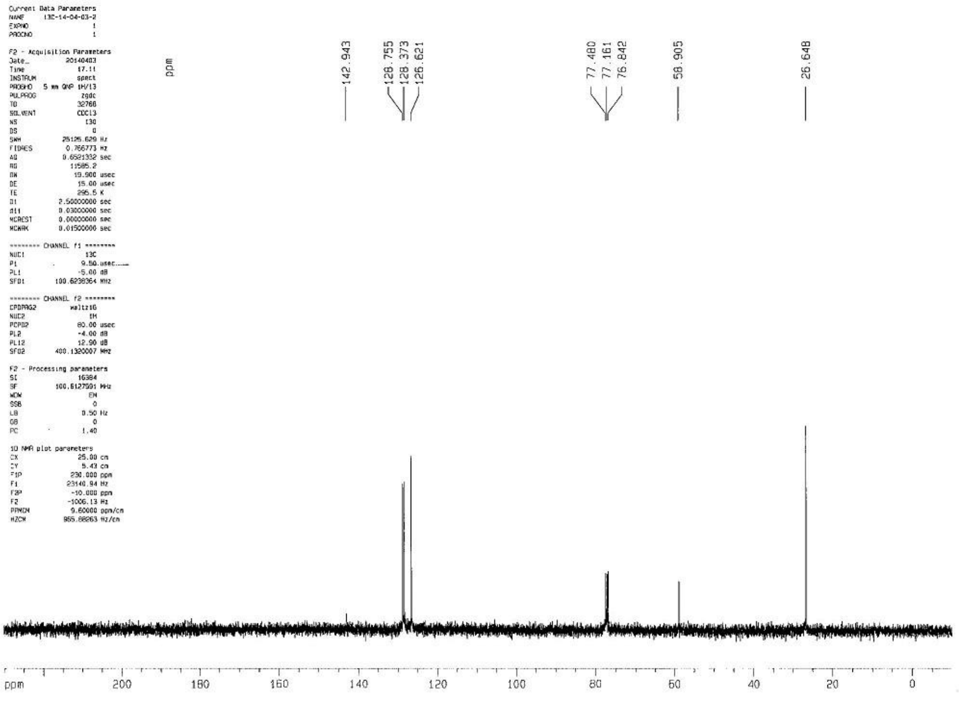

ppm
142.943
128.755
128.373
126.621
77.480
77.161
76.842
58.905
26.648
ppm
200
180
160
140
120
100
80
60
40
20
0

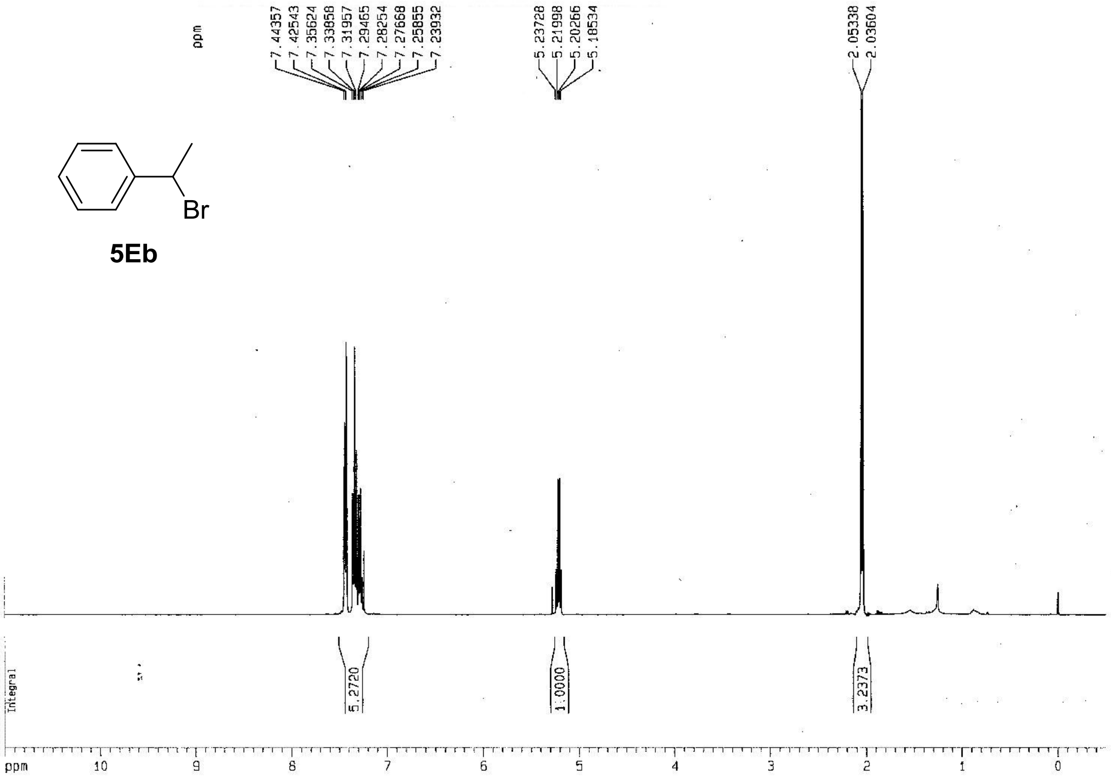
Br
ppm
7.44357
7.42543
7.35624
7.33858
7.31957
7.29465
7.28254
7.27668
7.25855
7.23932
5.23728
5.21998
5.20266
5.18534
2.05338
2.03604
Integral
5.2720
1.0000
3.2373
ppm 10 9 8 7 6 5 4 3 2 1 0

5Eb

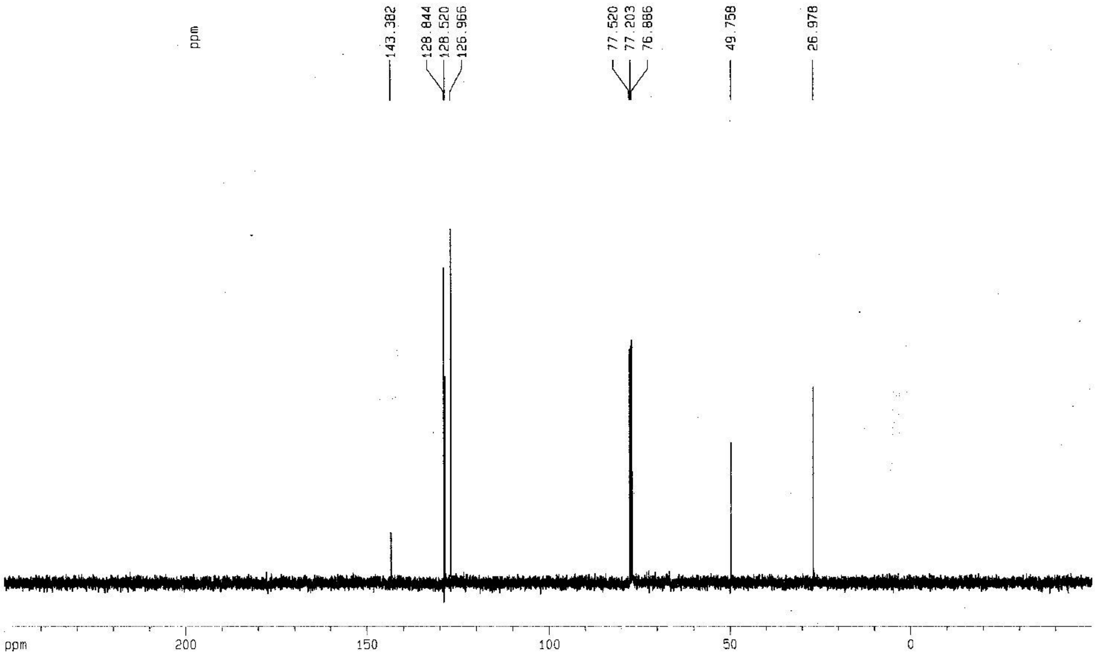
ppm
143.382
128.844
128.520
126.966
77.520
77.203
76.886
49.758
26.978
ppm 200 150 100 50 0

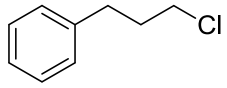
Cl

5Fa

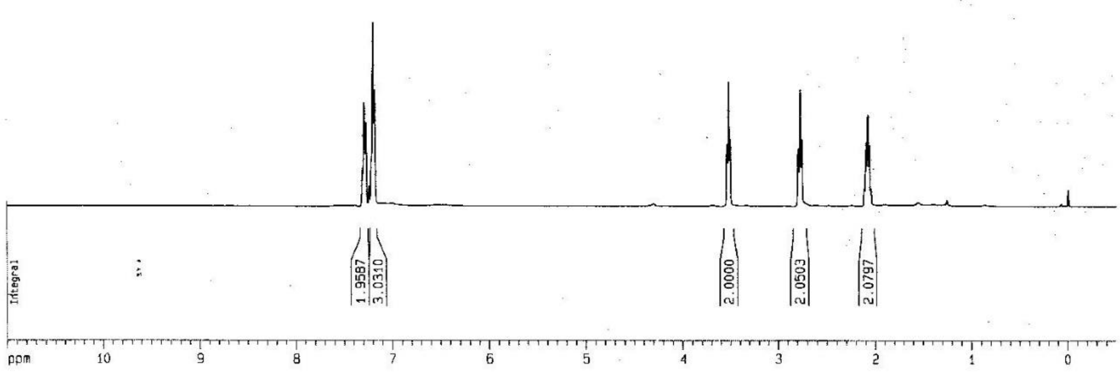
ppm
7.3126
7.2947
7.2769
7.2438
7.2047
7.1872
3.5410
3.5252
3.5098
2.7963
2.7780
2.7599
2.1178
2.1011
2.0842
2.0672
2.0512
0.0001
Integral
1.9587
3.0310
2.0000
2.0503
2.0797
ppm 10 9 8 7 6 5 4 3 2 1 0

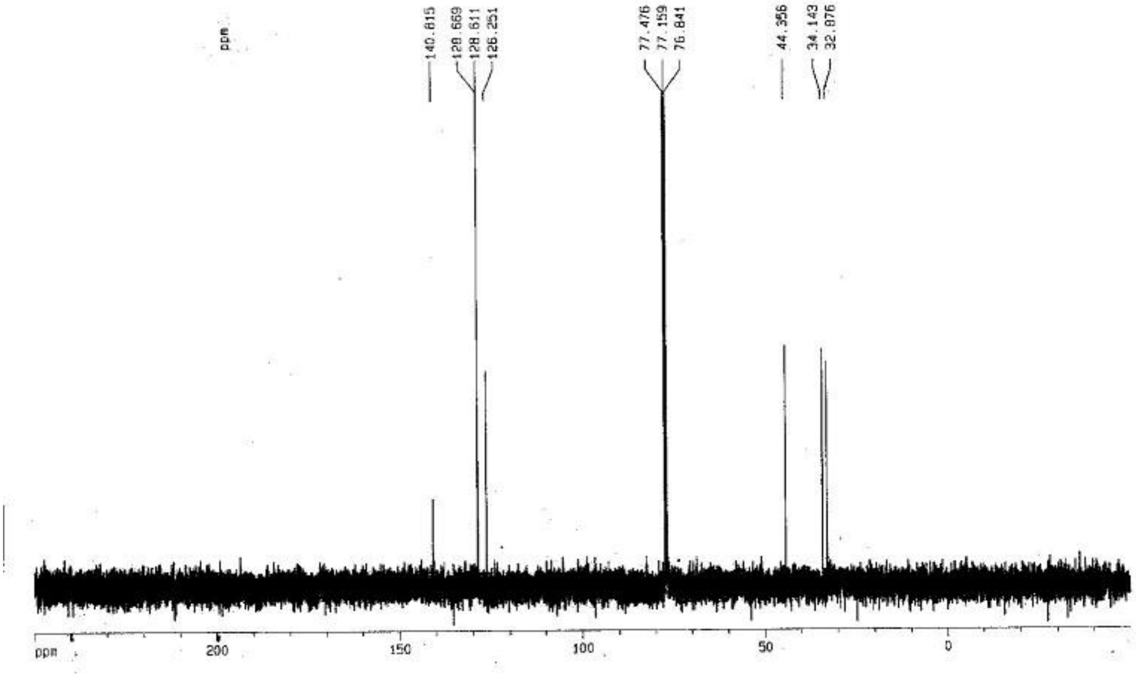
ppm
140.815
128.669
128.611
126.251
77.476
77.159
76.841
44.356
34.143
32.876
ppm 200 150 100 50 0

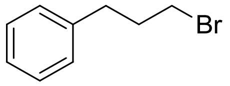

5Fb

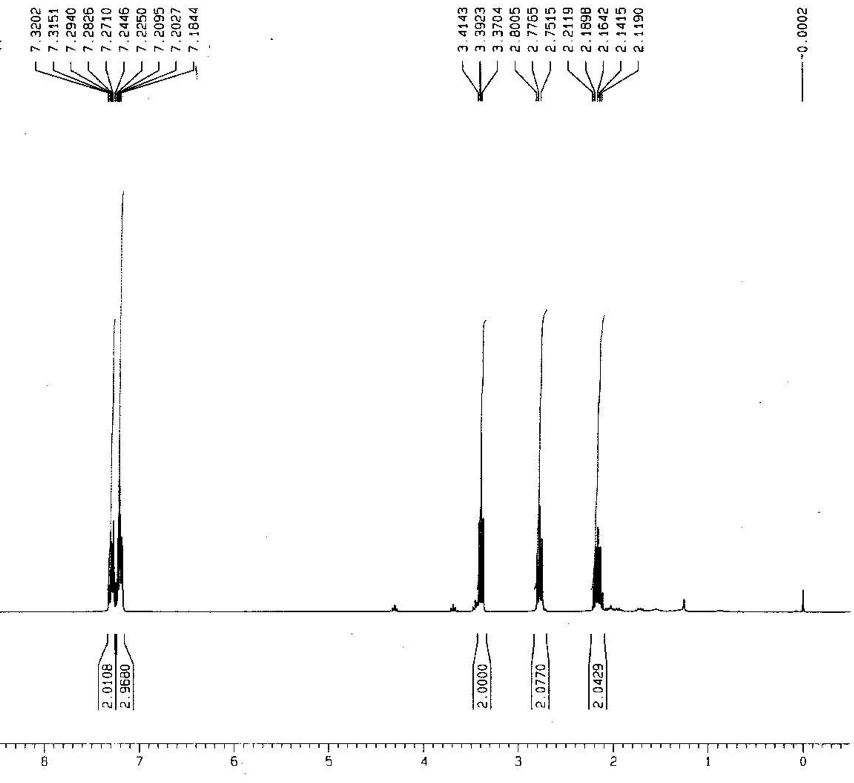
ppm
7.3202
7.3151
7.2940
7.2826
7.2710
7.2446
7.2250
7.2095
7.2027
7.1844
3.4143
3.3923
3.3704
2.8005
2.7765
2.7515
2.2119
2.1898
2.1642
2.1415
2.1190
0.0002
Integral
2.0108
2.9680
2.0000
2.0770
2.0429
ppm 10 9 8 7 6 5 4 3 2 1 0

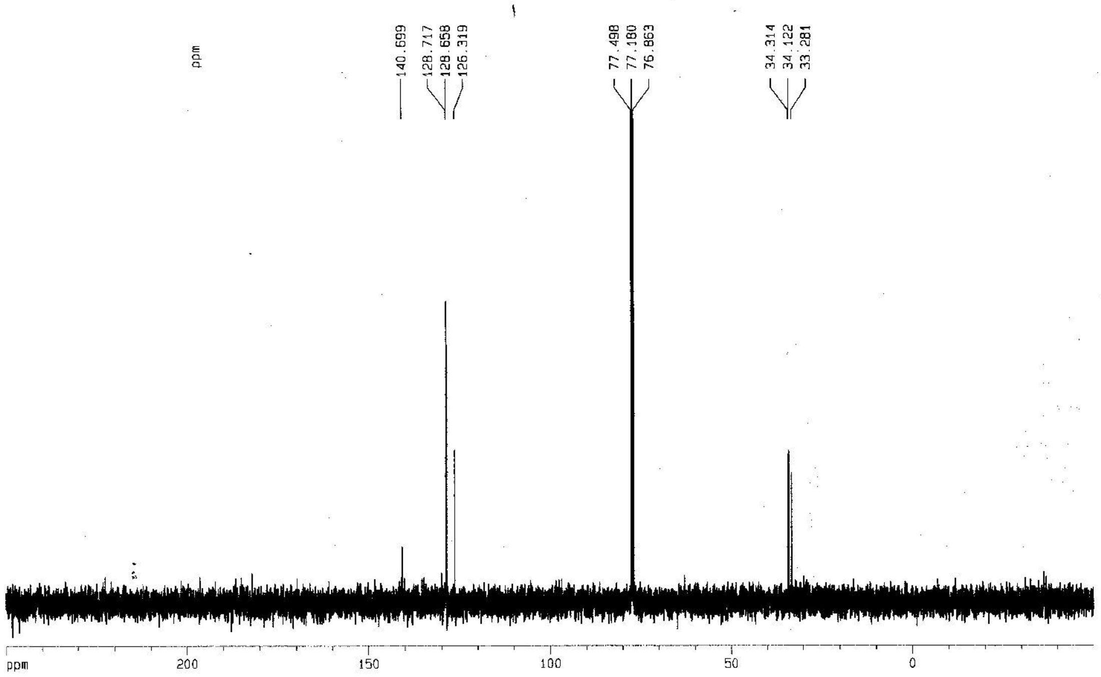
ppm
140.699
128.717
128.658
126.319
77.498
77.180
76.863
34.314
34.122
33.281
ppm 200 150 100 50 0

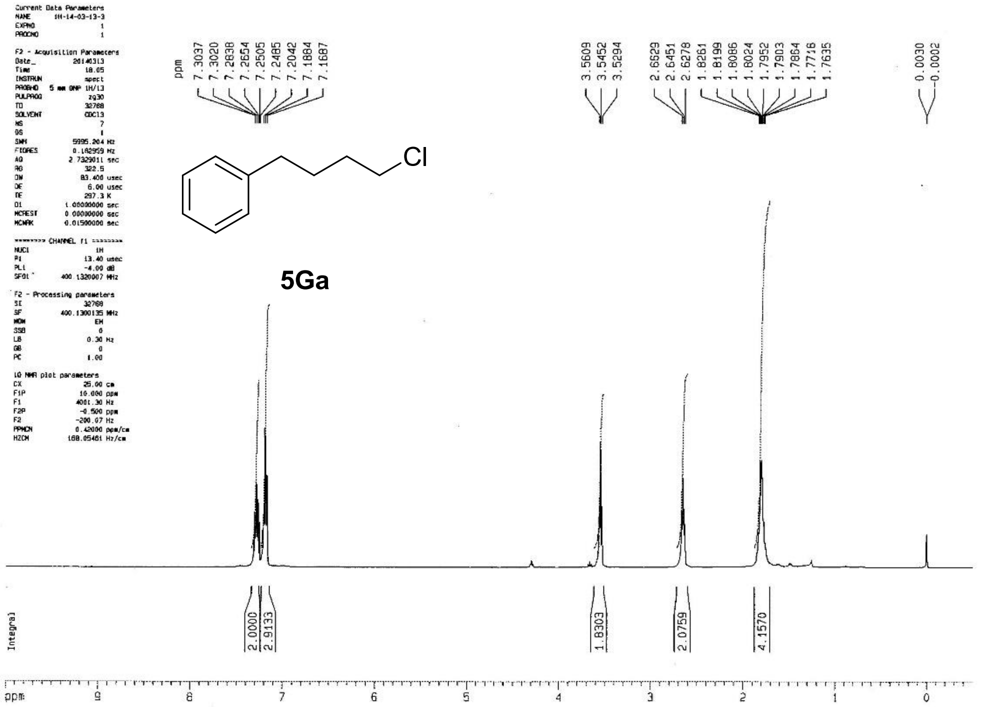
5Ga
Cl
7.3037
7.3020
7.2838
7.2654
7.2505
7.2485
7.2042
7.1884
7.1687
3.5609
3.5452
3.5294
2.6629
2.6451
2.6278
1.8261
1.8199
1.8086
1.8024
1.7952
1.7903
1.7864
1.7716
1.7635
0.0030
-0.0002
2.0000
2.9133
1.8303
2.0759
4.1570
Integral
ppm

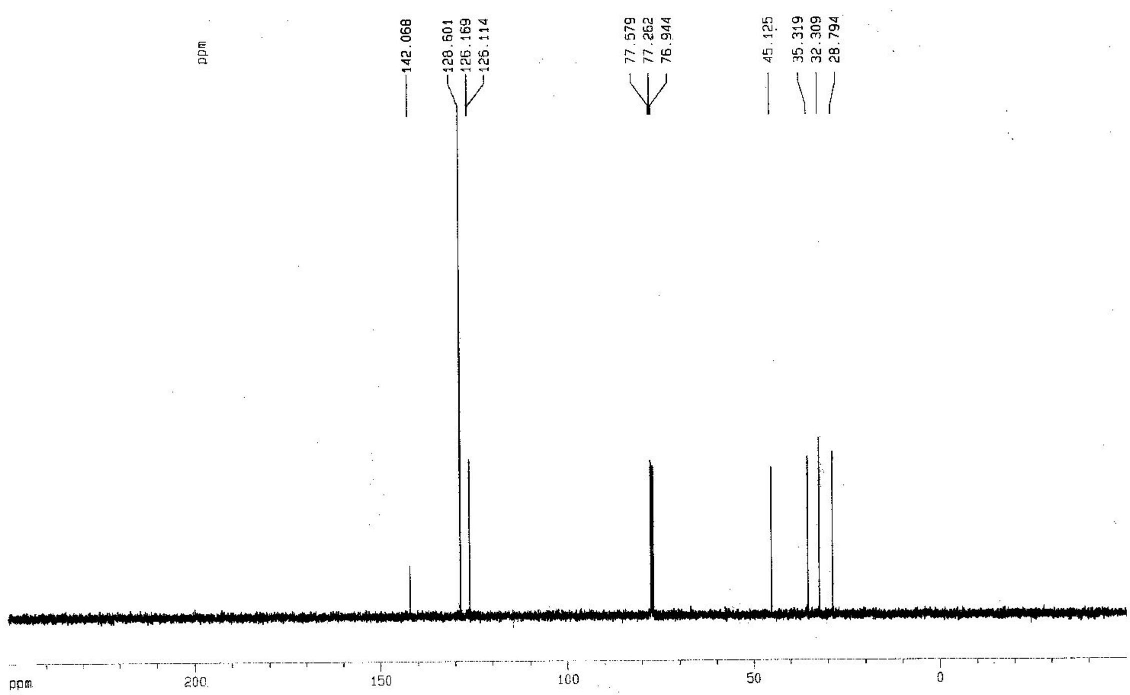
ppm
142.068
128.601
126.169
126.114
77.579
77.262
76.944
45.125
35.319
32.309
28.794
200
150
100
50
0

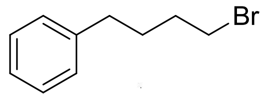
Br

5Gb

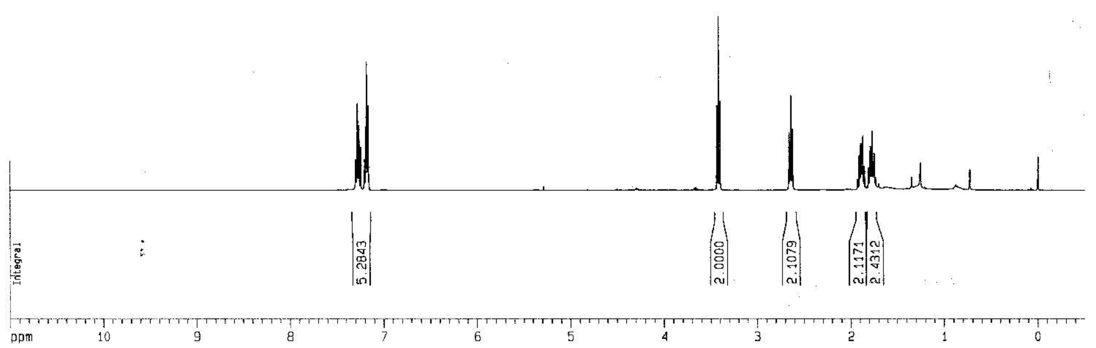
ppm
7.30312
7.29925
7.28375
7.27023
7.26632
7.24832
7.20679
7.20401
7.18803
7.18436
7.16732
3.43276
3.41626
3.39941
2.66113
2.64212
2.62345
1.91206
1.90991
1.89528
1.89154
1.88612
1.87840
1.87358
1.85754
1.81045
1.79911
1.79230
1.79071
1.78129
1.77353
1.75266
1.25406
0.73264
0.00001
Integral
5.2843
2.0000
2.1079
2.1171
2.4312
ppm 10 9 8 7 6 5 4 3 2 1 0

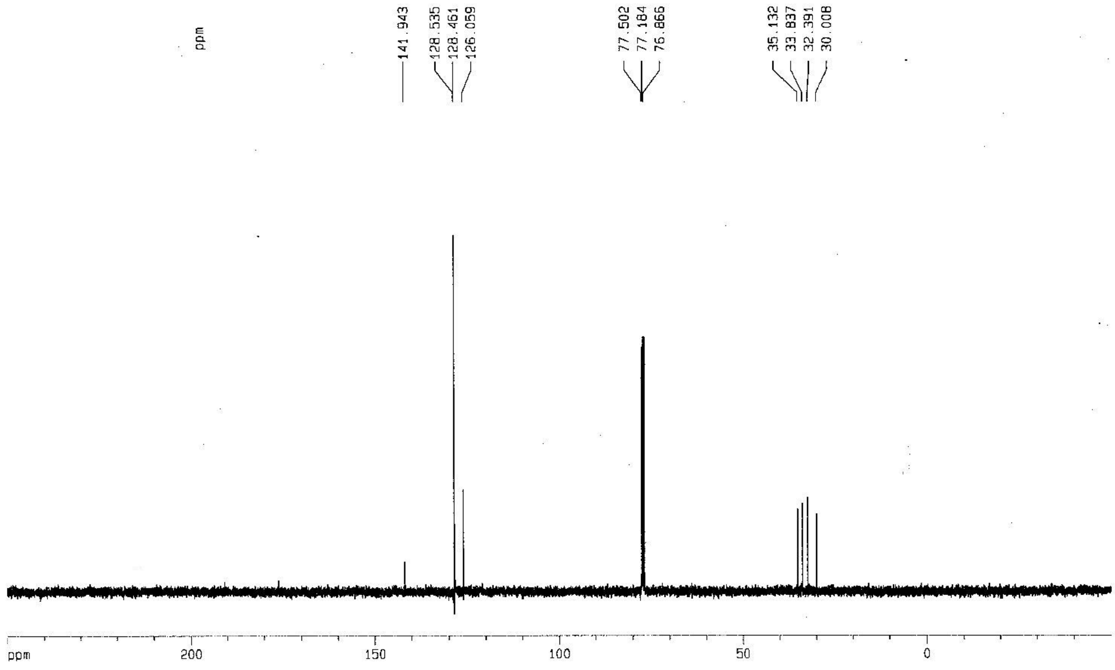
ppm
141.943
128.535
128.461
126.059
77.502
77.184
76.866
35.132
33.837
32.391
30.008
ppm 200 150 100 50 0

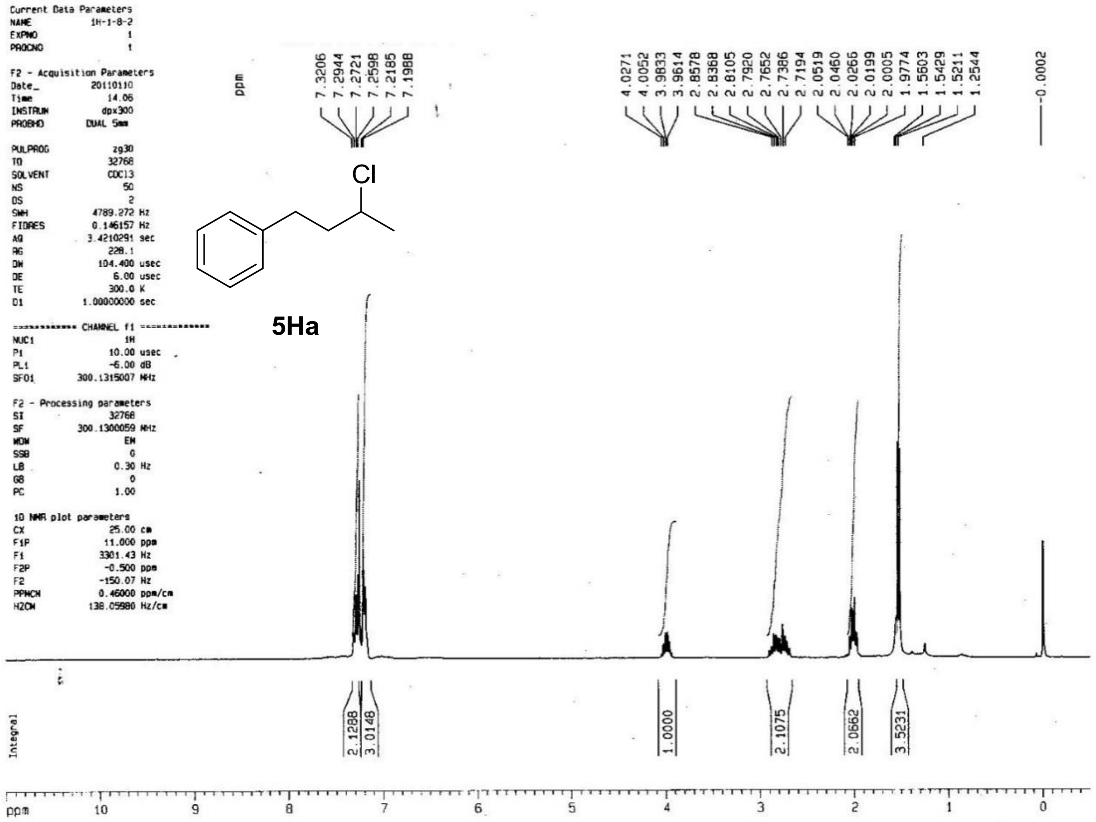
Current Data Parameters
NAME 1H-I-8-2
EXPNO 1
PROCNO 1
F2 - Acquisition Parameters
Date_ 20110110
Time 14.06
INSTRUM dpx300
PROBHD DUAL 5mm
PULPROG zg30
TD 32768
SOLVENT CDCl3
NS 50
DS 2
SWH 4789.272 Hz
FIDRES 0.146157 Hz
AQ 3.4210291 sec
RG 228.1
DW 104.400 usec
DE 6.00 usec
TE 300.0 K
D1 1.00000000 sec
CHANNEL f1
NUC1 1H
P1 10.00 usec
PL1 -6.00 dB
SFO1 300.1315007 MHz
F2 - Processing parameters
SI 32768
SF 300.1300059 MHz
WDW EM
SSB 0
LB 0.30 Hz
GB 0
PC 1.00
1D NMR plot parameters
CX 25.00 cm
F1P 11.000 ppm
F1 3301.43 Hz
F2P -0.500 ppm
F2 -150.07 Hz
PPMCM 0.46000 ppm/cm
HZCM 138.05980 Hz/cm
ppm
7.3206
7.2944
7.2721
7.2598
7.2185
7.1988
4.0271
4.0052
3.9833
3.9614
2.8578
2.8368
2.8105
2.7920
2.7652
2.7386
2.7194
2.0519
2.0460
2.0266
2.0199
2.0005
1.9774
1.5603
1.5429
1.5211
1.2544
-0.0002
Integral
2.1288
3.0148
1.0000
2.1075
2.0662
3.5231
ppm 10 9 8 7 6 5 4 3 2 1 0

5Ha

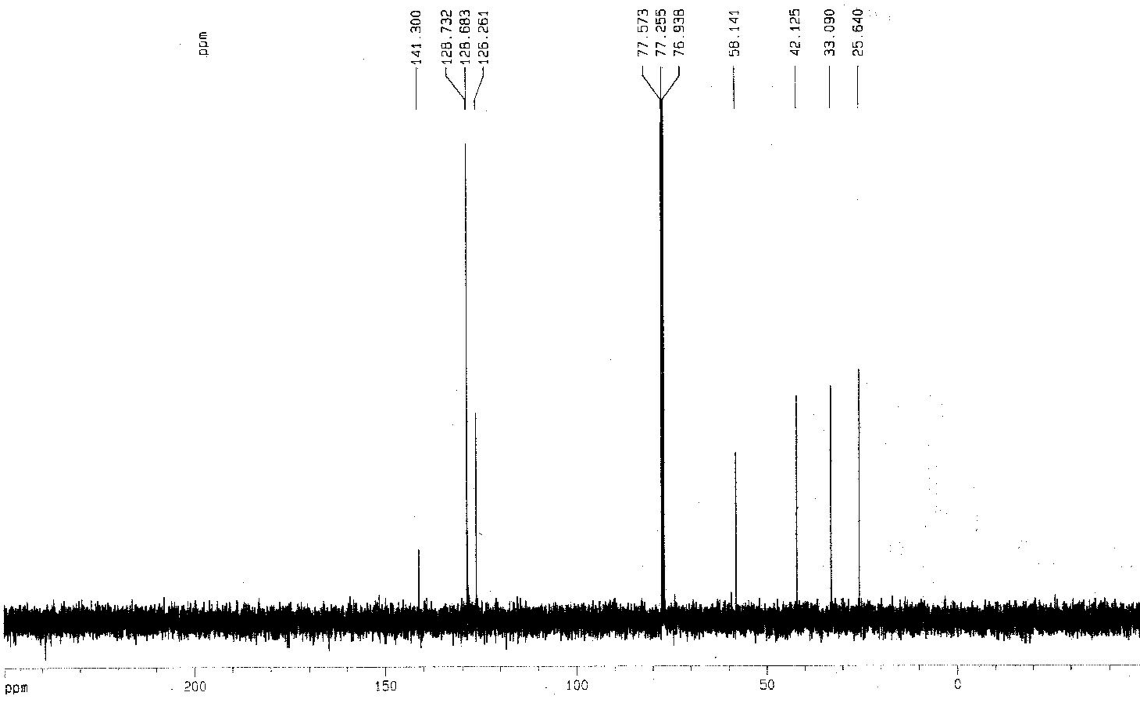
ppm
141.300
128.732
128.683
126.261
77.573
77.255
76.938
58.141
42.125
33.090
25.640
ppm 200 150 100 50 0

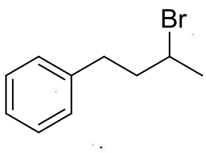

5Hb

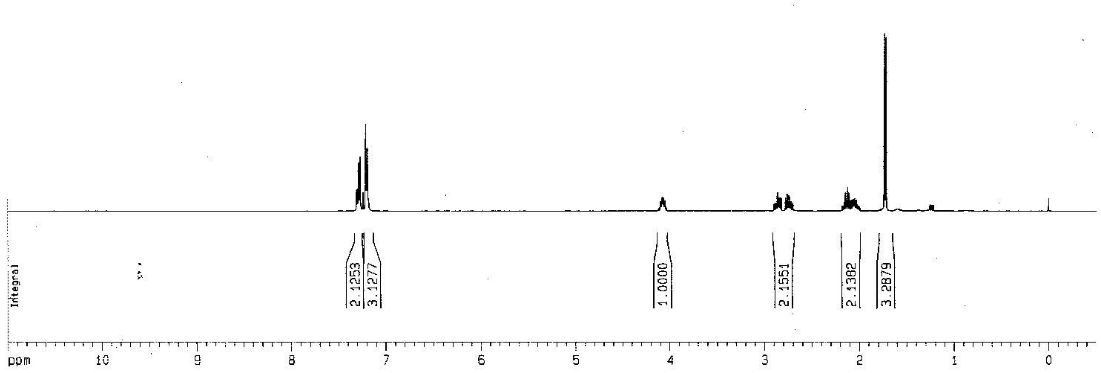
ppm
7.3083
7.2891
7.2876
7.2794
7.2713
7.2427
7.2142
7.1978
7.1838
7.1804
4.0878
4.0823
4.0768
4.0712
4.0657
4.0603
2.8621
2.8493
2.8403
2.8270
2.7787
2.7604
2.7571
2.7387
2.1458
2.1323
2.1234
2.1100
2.0820
2.0709
2.0641
2.0596
2.0529
2.0485
2.0305
1.7344
1.7178
1.2538
1.2358
-0.0002
Integral
2.1253
3.1277
1.0000
2.1551
2.1382
3.2879
ppm 10 9 8 7 6 5 4 3 2 1 0

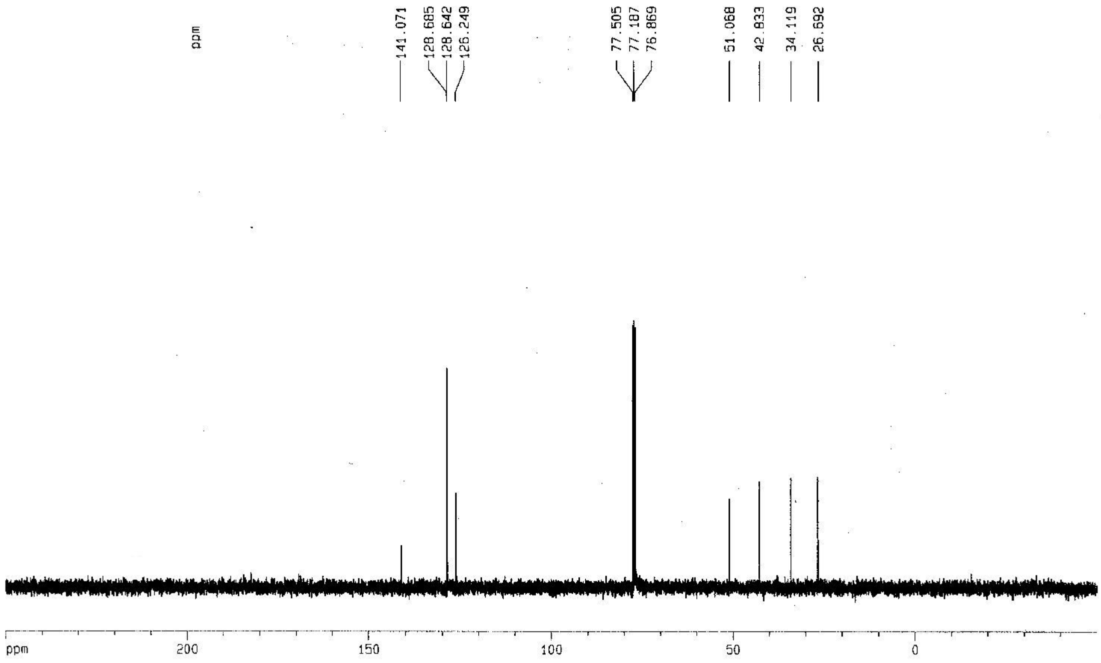
ppm
141.071
128.685
128.642
126.249
77.505
77.187
76.869
51.068
42.833
34.119
26.692
ppm 200 150 100 50 0

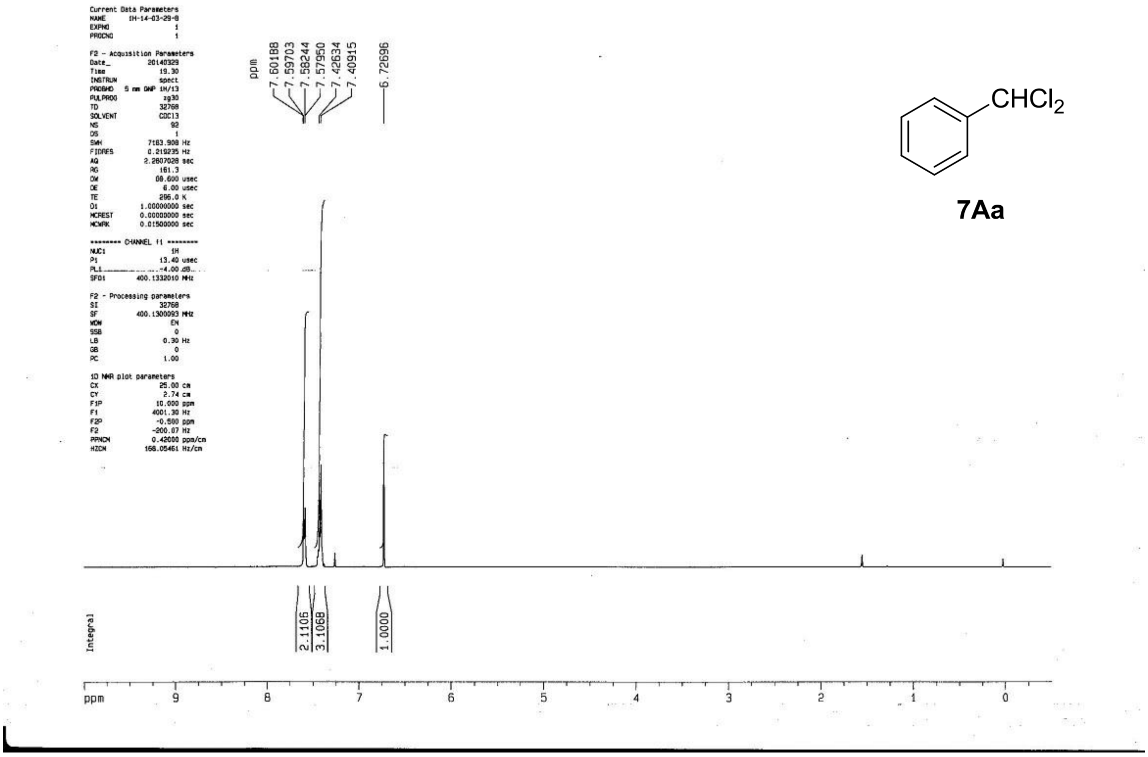
CHCl2
7Aa
ppm
7.60188
7.59703
7.58244
7.57950
7.42634
7.40915
6.72696
Integral
2.1106
3.1068
1.0000
ppm
9
8
7
6
5
4
3
2
1
0

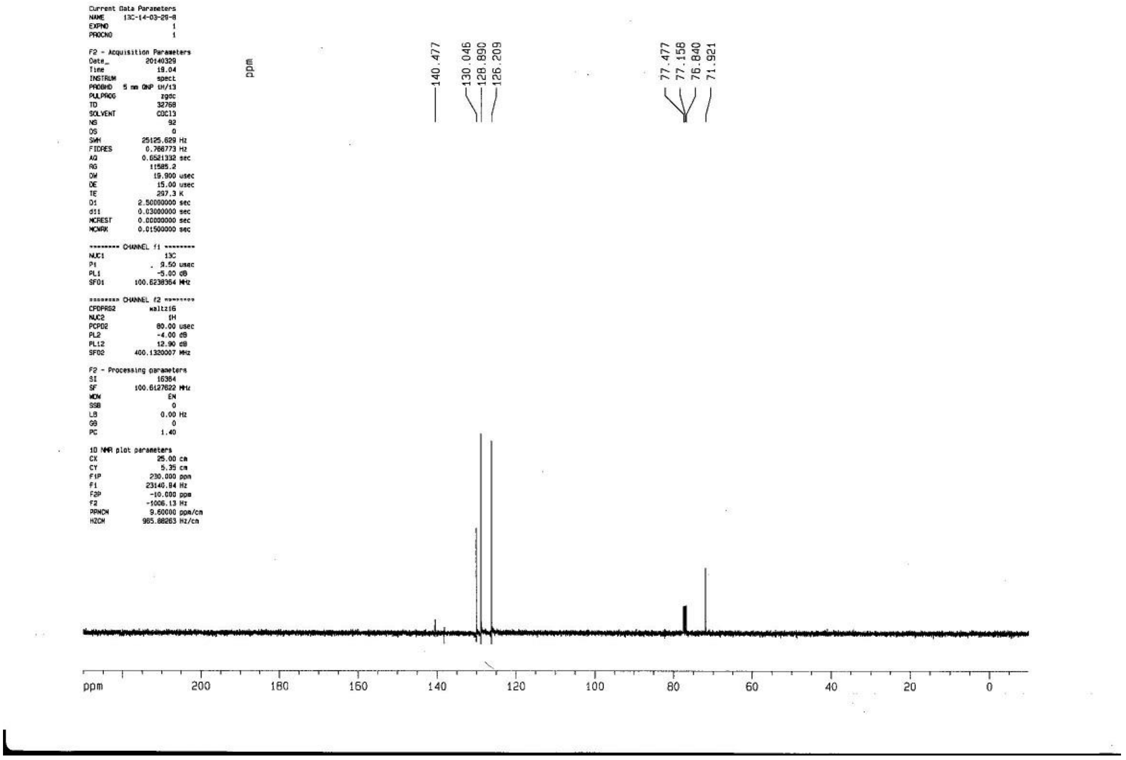
ppm
140.477
130.046
128.890
126.209
77.477
77.158
76.840
71.921
ppm
200
180
160
140
120
100
80
60
40
20
0

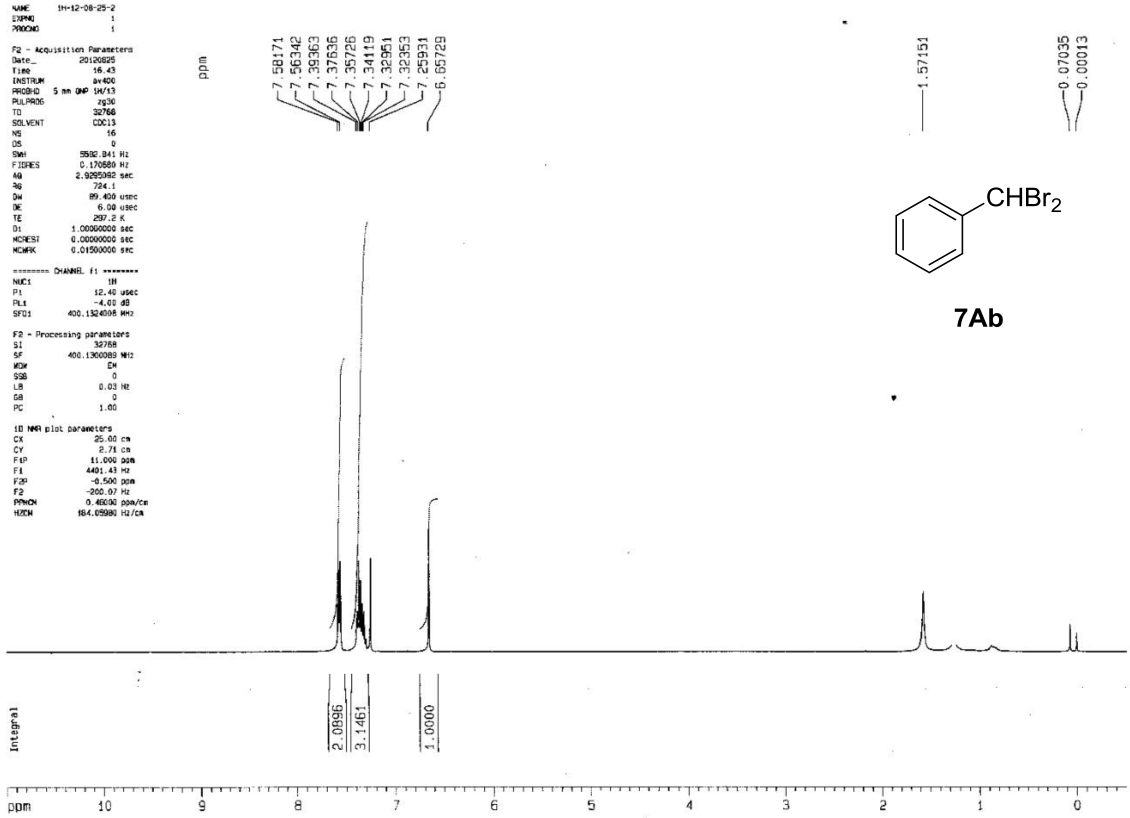
7.58171
7.56342
7.39363
7.37636
7.35726
7.34119
7.32951
7.32353
7.25931
6.65729
1.57151
0.07035
0.00013
CHBr2
7Ab
Integral
2.0896
3.1461
1.0000
ppm

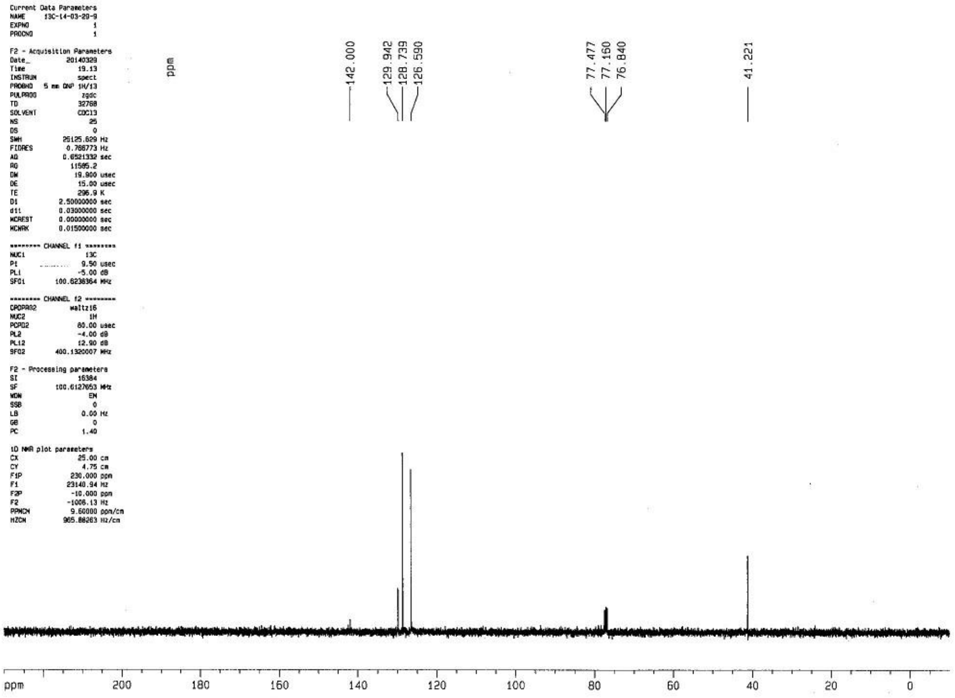
142.000
129.942
128.739
126.590
77.477
77.160
76.840
41.221
ppm

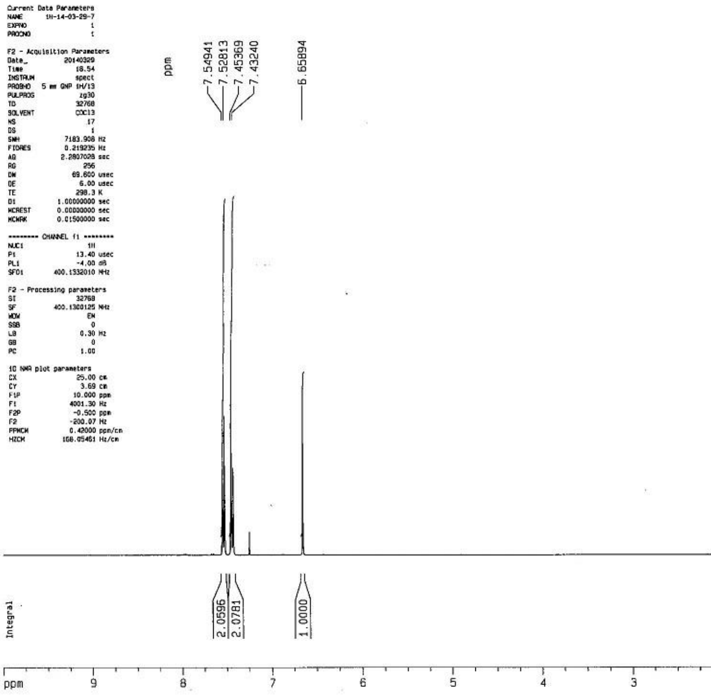

7.54941
7.52813
7.45369
7.43240
6.65894
2.0596
2.0781
1.0000
Integral
ppm

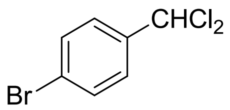

7Ba

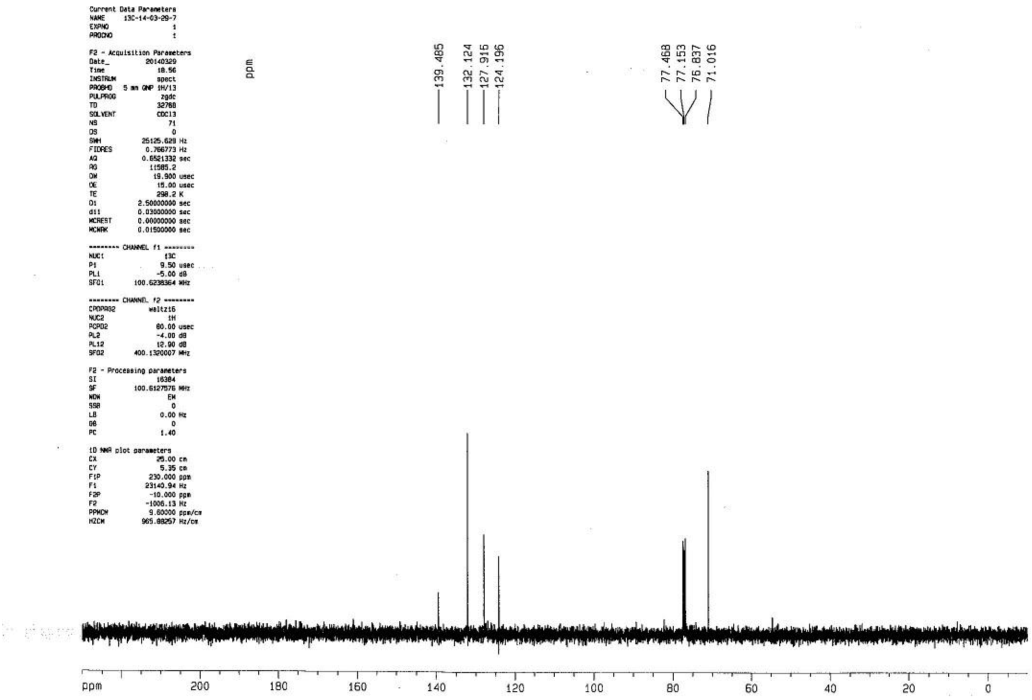

139.485
132.124
127.915
124.196
77.468
77.153
76.837
71.016
ppm

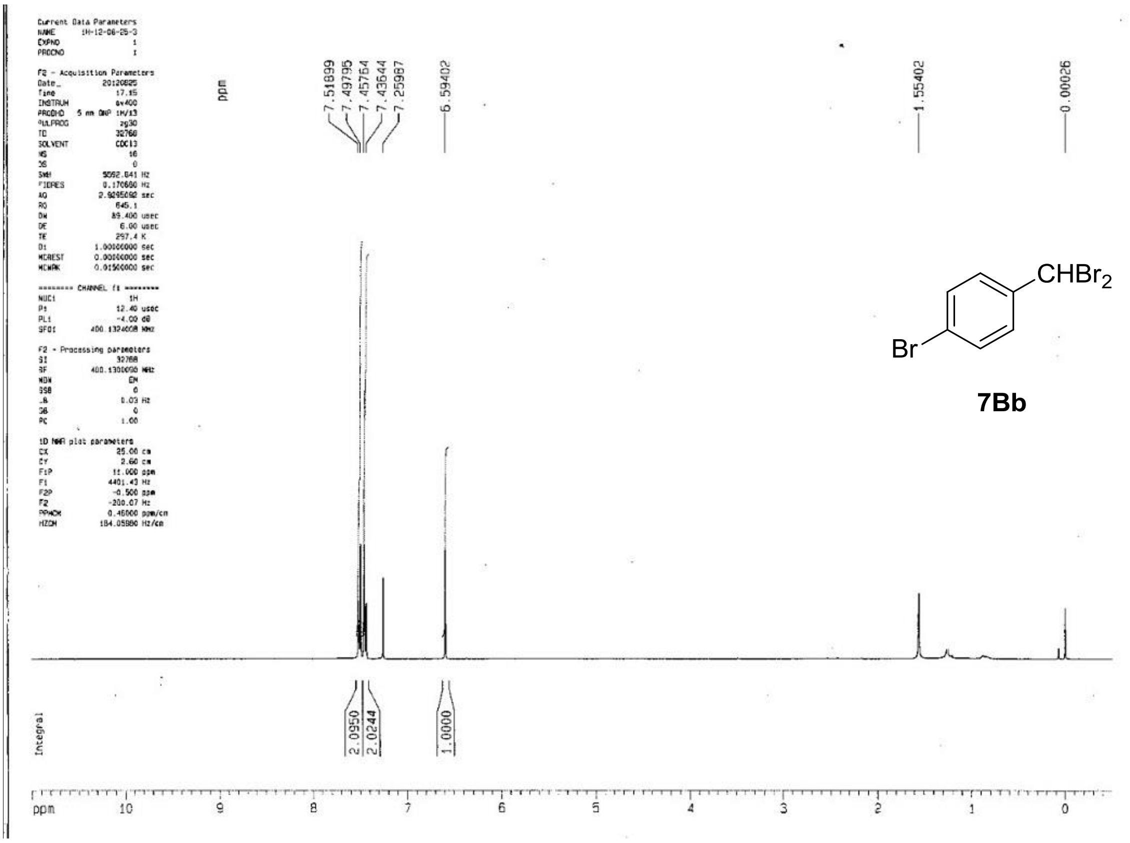
7.51899
7.49795
7.45764
7.43644
7.25987
6.59402
1.55402
0.00026
CHBr$_2$
Br
7Bb
2.0950
2.0244
1.0000
Integral
ppm

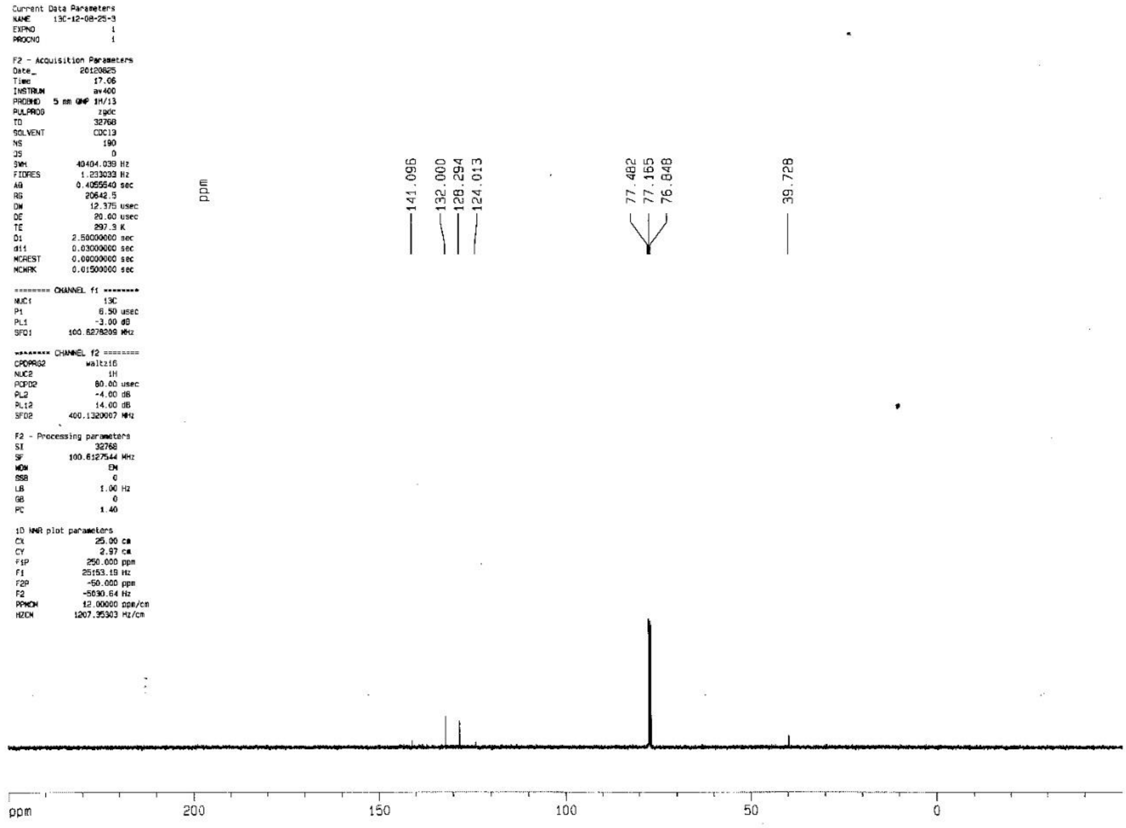
141.096
132.000
128.294
124.013
77.482
77.165
76.848
39.728
ppm

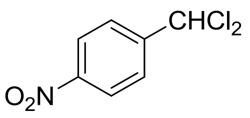

CHCl2
O2N

7Ca

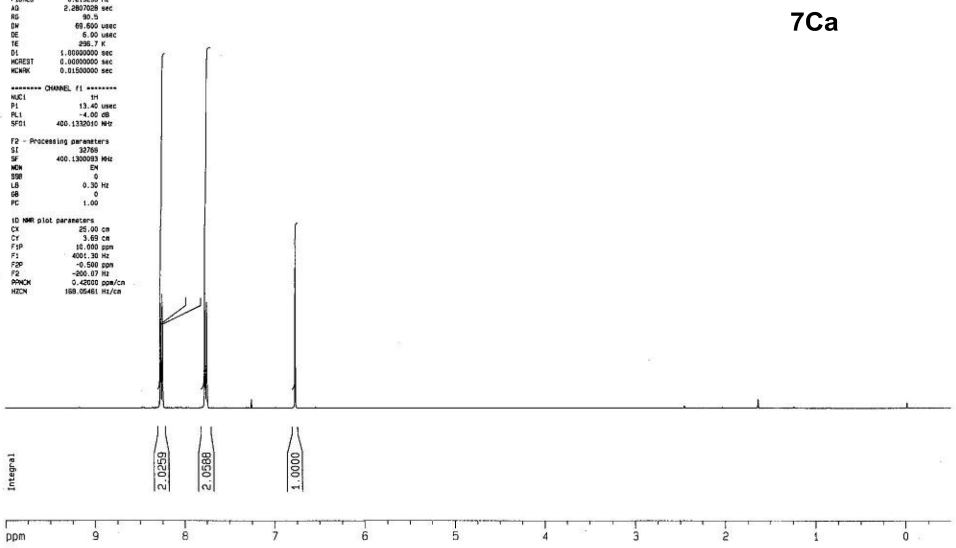

8.27212
8.25037
7.77400
7.75224
6.77288
2.0259
2.0588
1.0000
Integral
ppm

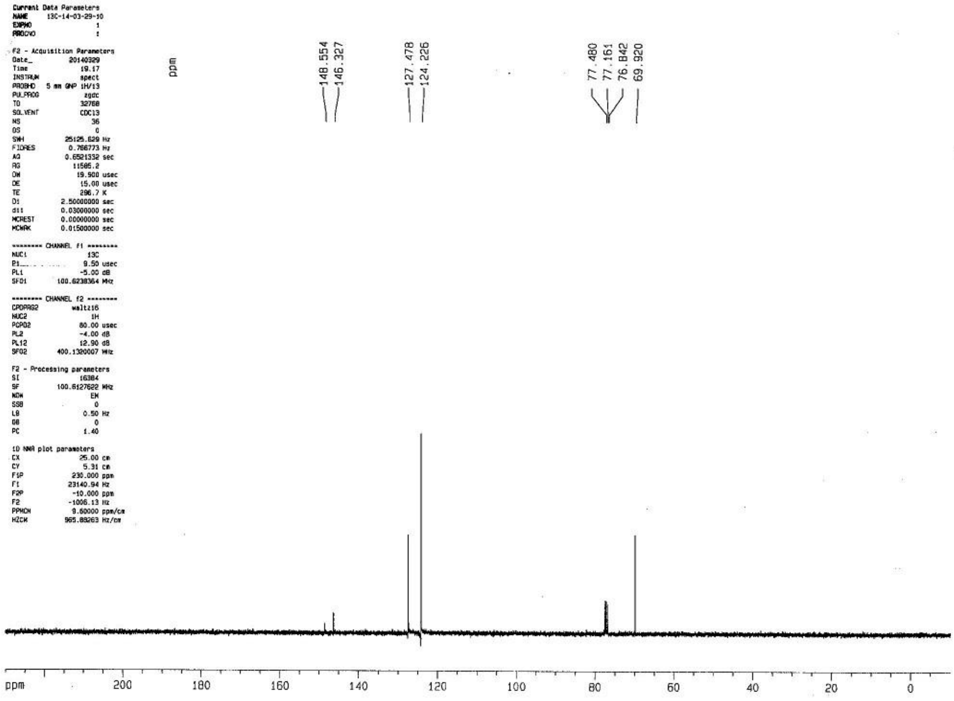

148.554
146.327
127.478
124.226
77.480
77.161
76.842
69.920
ppm

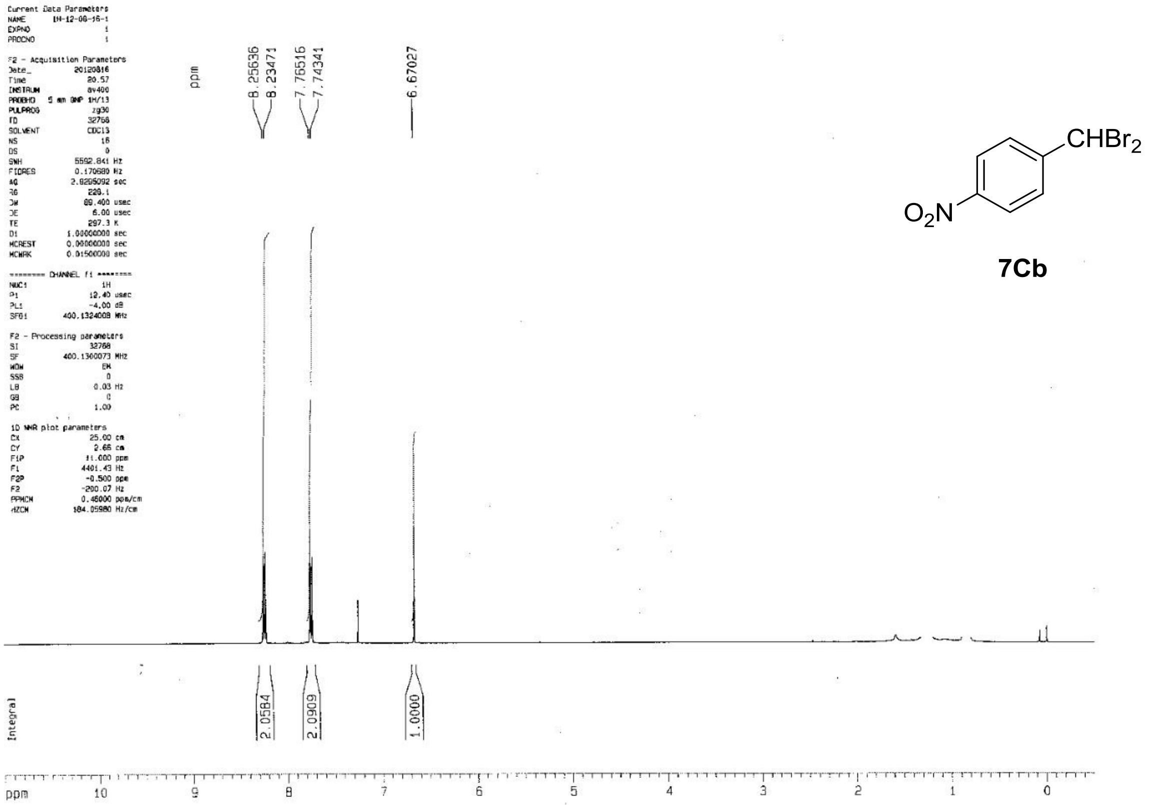
CHBr2
O2N
ppm
8.25636
8.23471
7.76516
7.74341
6.67027
Integral
2.0584
2.0909
1.0000
ppm
10
9
8
7
6
5
4
3
2
1
0

7Cb

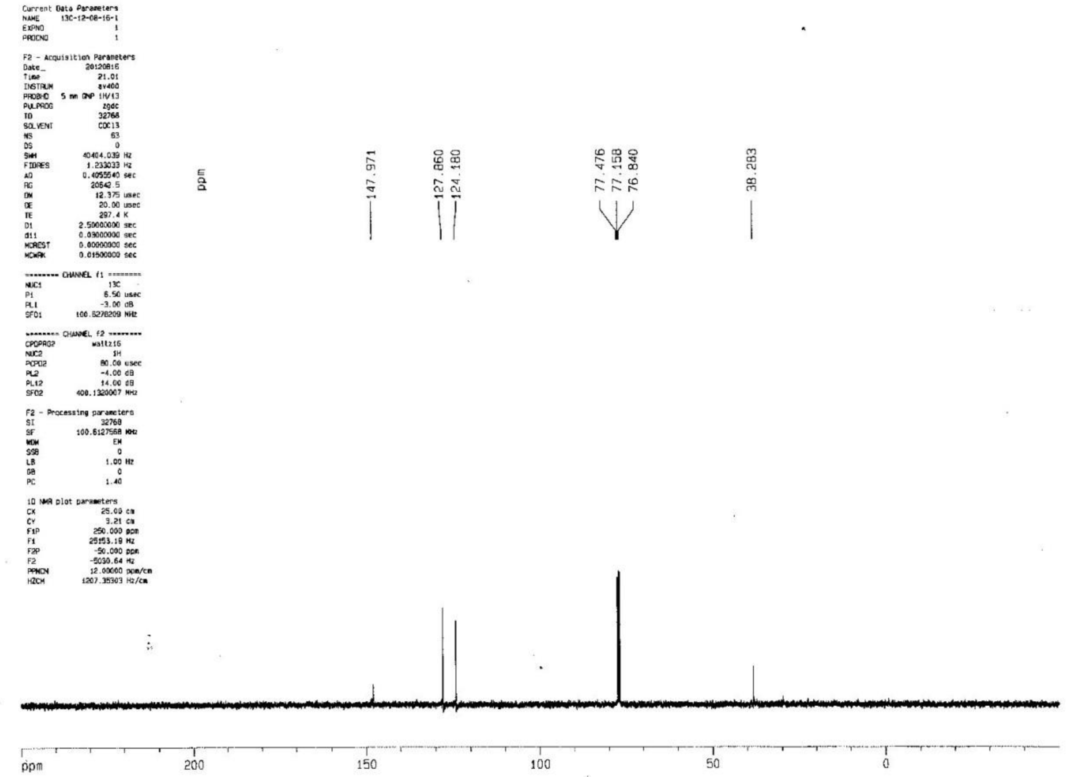
ppm
147.971
127.860
124.180
77.476
77.158
76.840
38.283
ppm
200
150
100
50
0

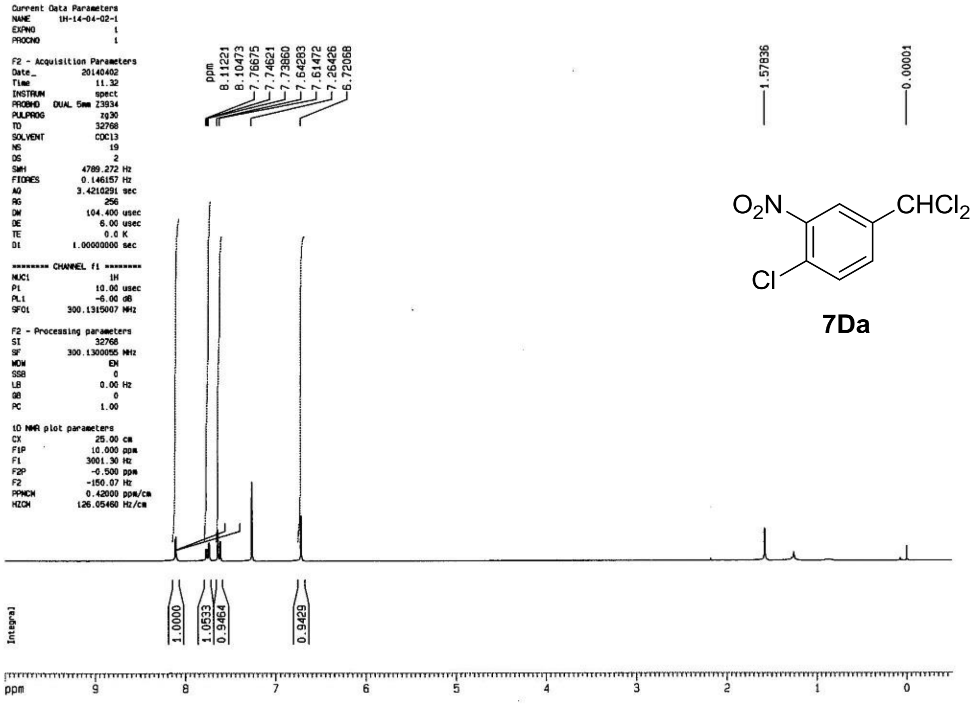
ppm
8.11221
8.10473
7.76675
7.74621
7.73860
7.64283
7.61472
7.26426
6.72068
1.57836
0.00001
O_2N
$CHCl_2$
Cl
7Da
Integral
1.0000
1.0533
0.9464
0.9429
ppm 9 8 7 6 5 4 3 2 1 0

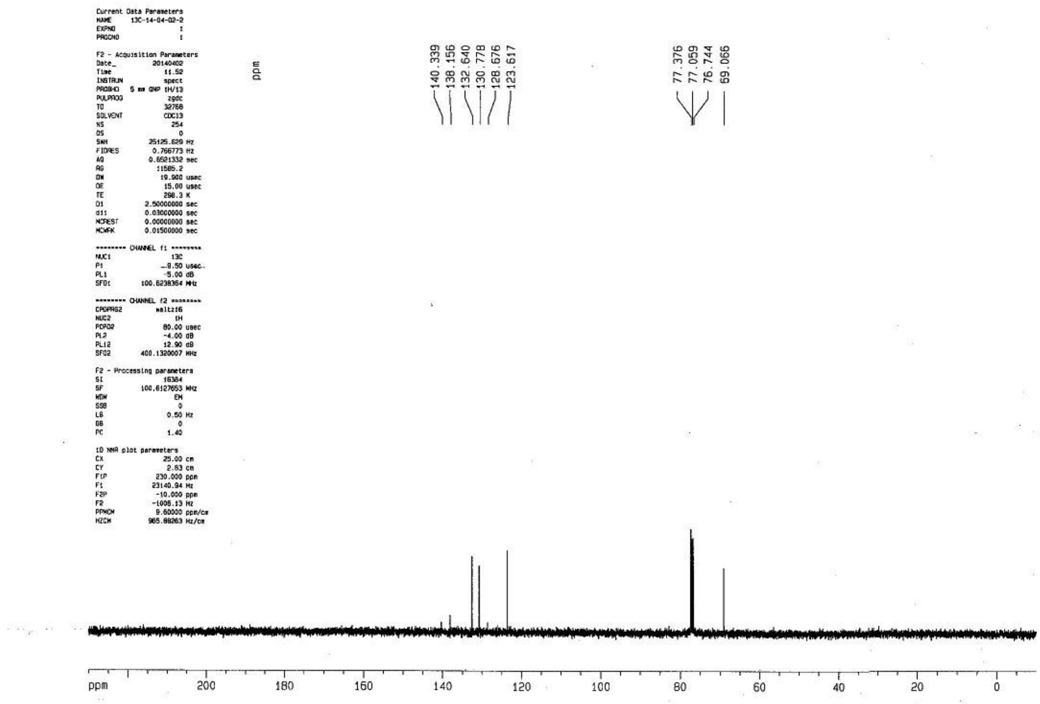
ppm
140.339
138.156
132.640
130.778
128.676
123.617
77.376
77.059
76.744
69.066
ppm 200 180 160 140 120 100 80 60 40 20 0

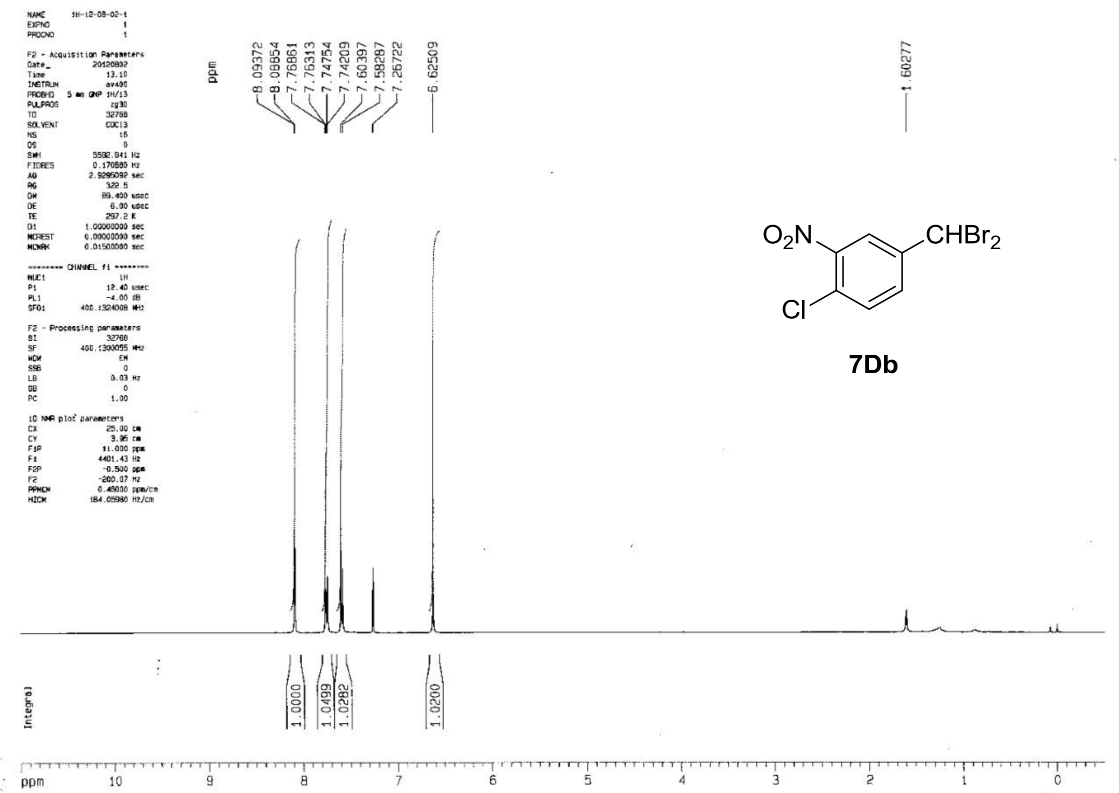
8.09372
8.08854
7.76861
7.76313
7.74754
7.74209
7.60397
7.58287
7.26722
6.62509
1.60277
O2N
CHBr2
Cl
7Db
Integral
1.0000
1.0499
1.0282
1.0200
ppm

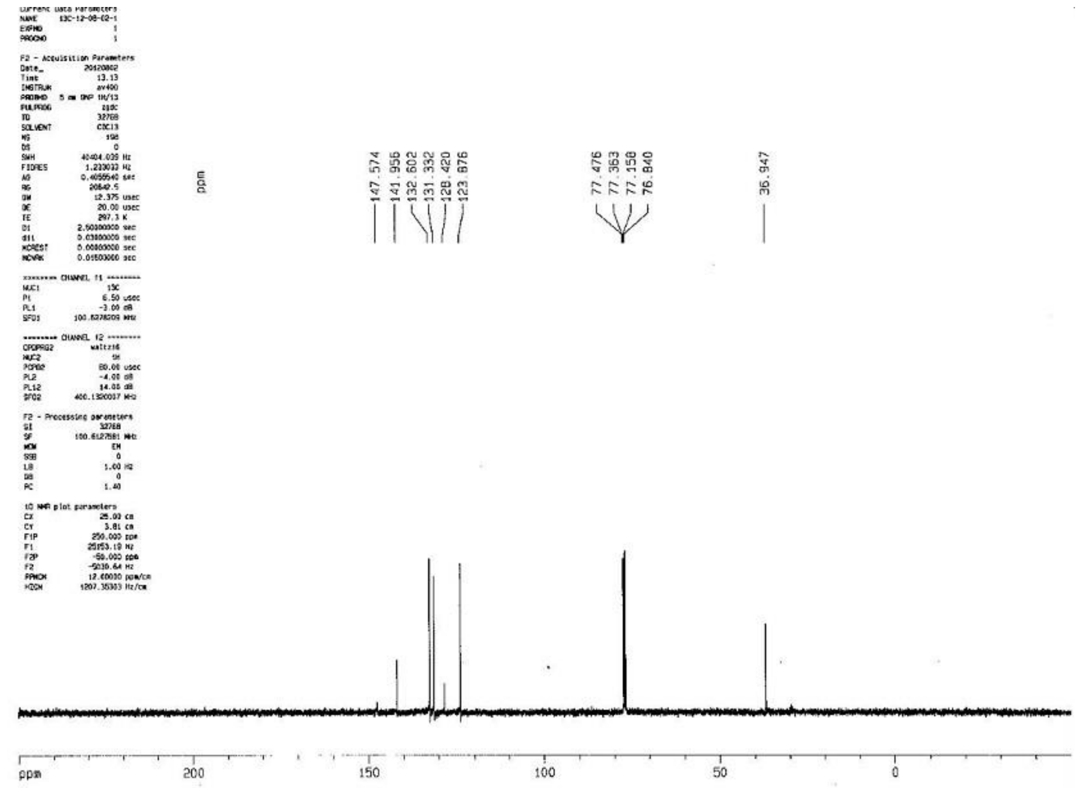
147.574
141.956
132.602
131.332
128.420
123.876
77.476
77.363
77.158
76.840
36.947
ppm

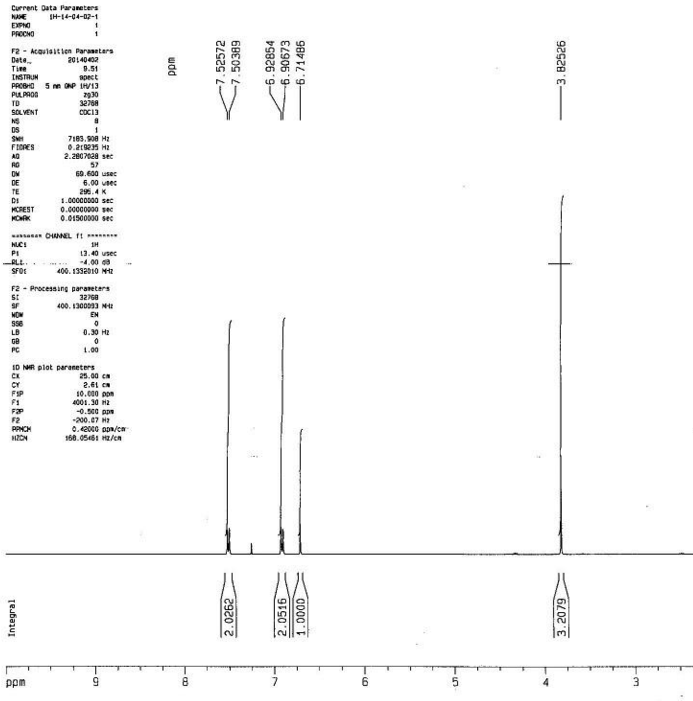
7.52572
7.50389
6.92854
6.90673
6.71486
3.82526
2.0262
2.0516
1.0000
3.2079
Integral
ppm

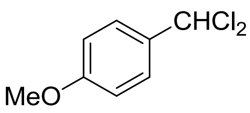
CHCl2
MeO

7Ea

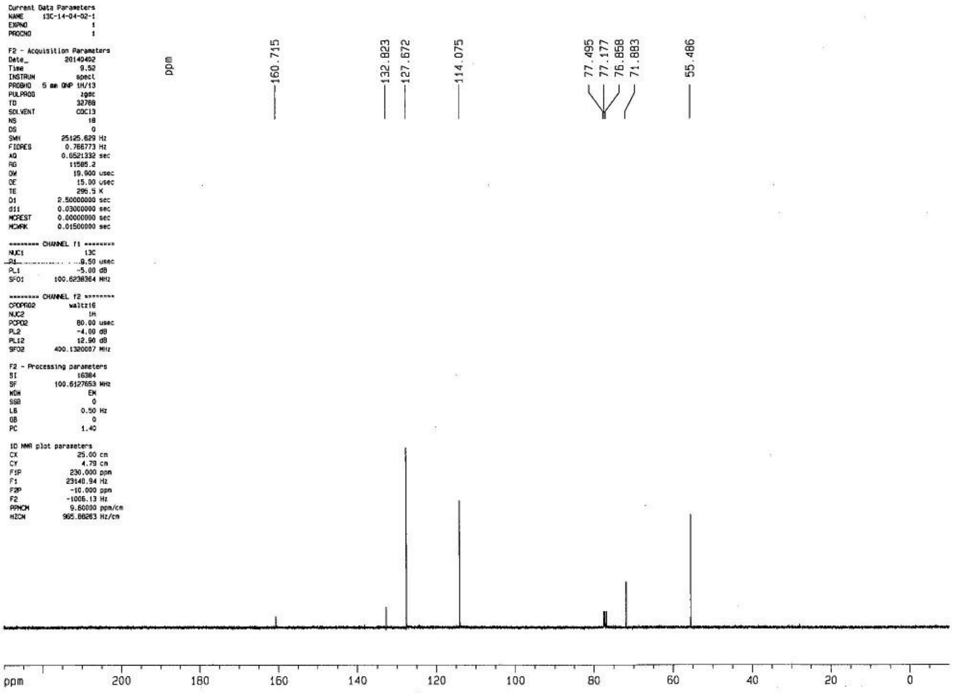
160.715
132.823
127.672
114.075
77.495
77.177
76.858
71.883
55.486
ppm

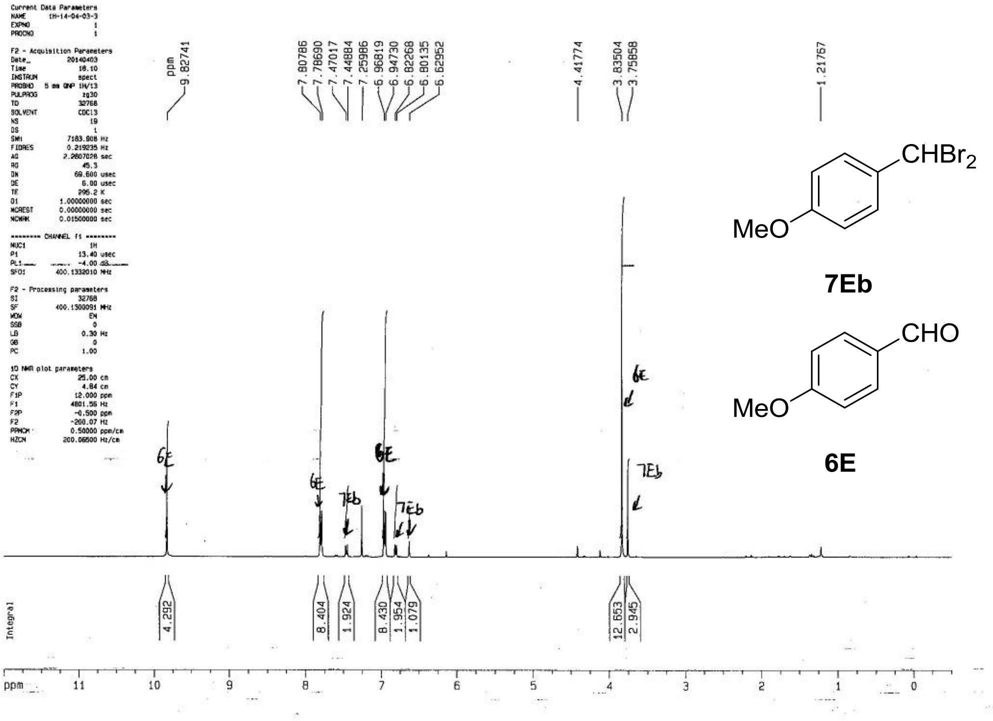
CHBr2
MeO
7Eb
CHO
MeO
6E
9.82741
7.80786
7.78690
7.47017
7.44884
7.25986
6.96819
6.94730
6.82268
6.80135
6.62952
4.41774
3.83504
3.75858
1.21767
4.292
8.404
1.924
8.430
1.954
1.079
12.653
2.945
Integral
ppm

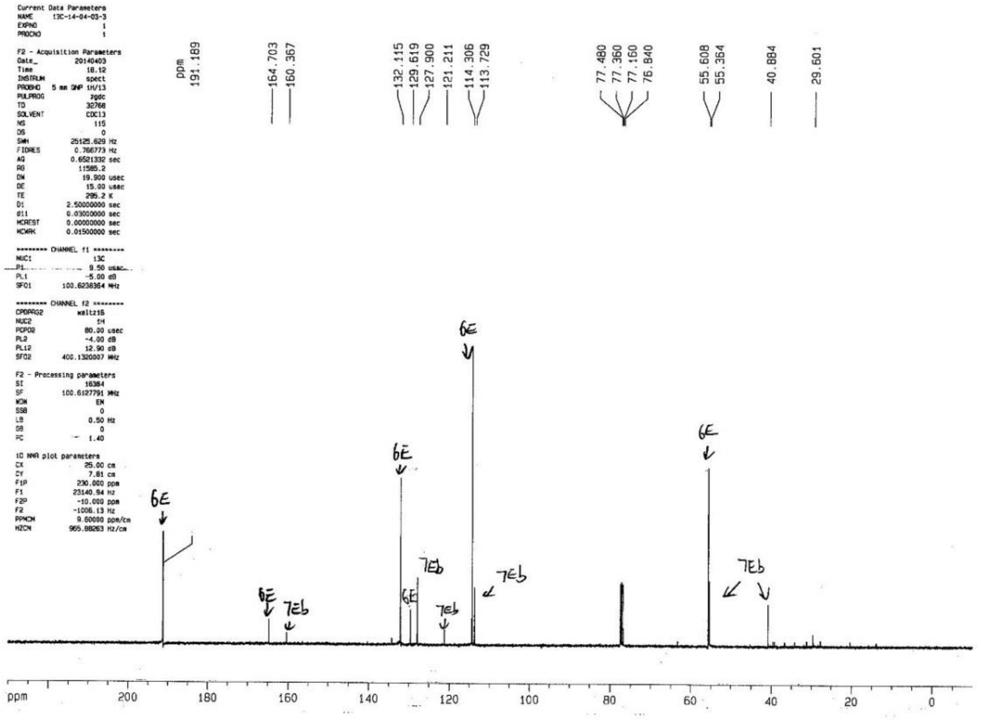
191.189
164.703
160.367
132.115
129.619
127.900
121.211
114.306
113.729
77.480
77.360
77.160
76.840
55.608
55.364
40.884
29.601
ppm

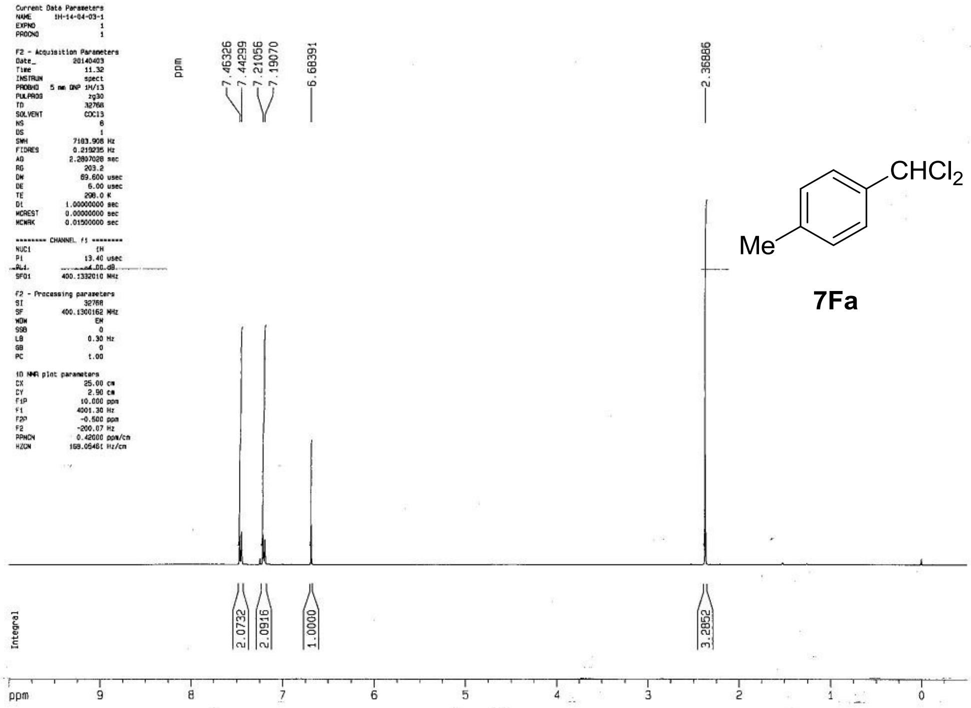
7.46326
7.44299
7.21056
7.19070
6.68391
2.36886
CHCl2
Me
7Fa
2.0732
2.0916
1.0000
3.2852
Integral
ppm

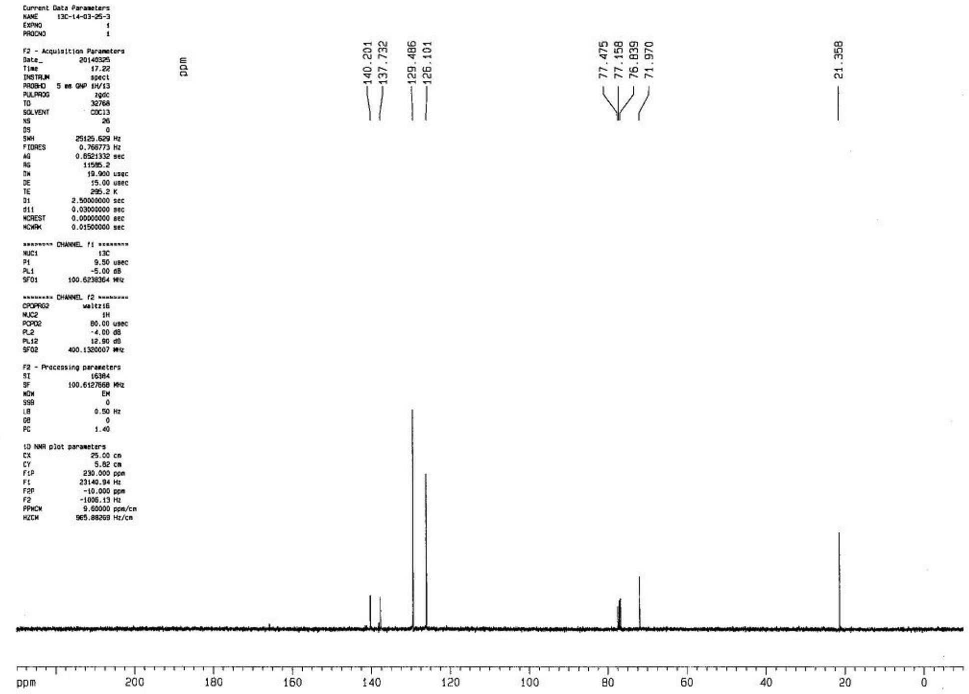
140.201
137.732
129.486
126.101
77.475
77.158
76.839
71.970
21.358
ppm

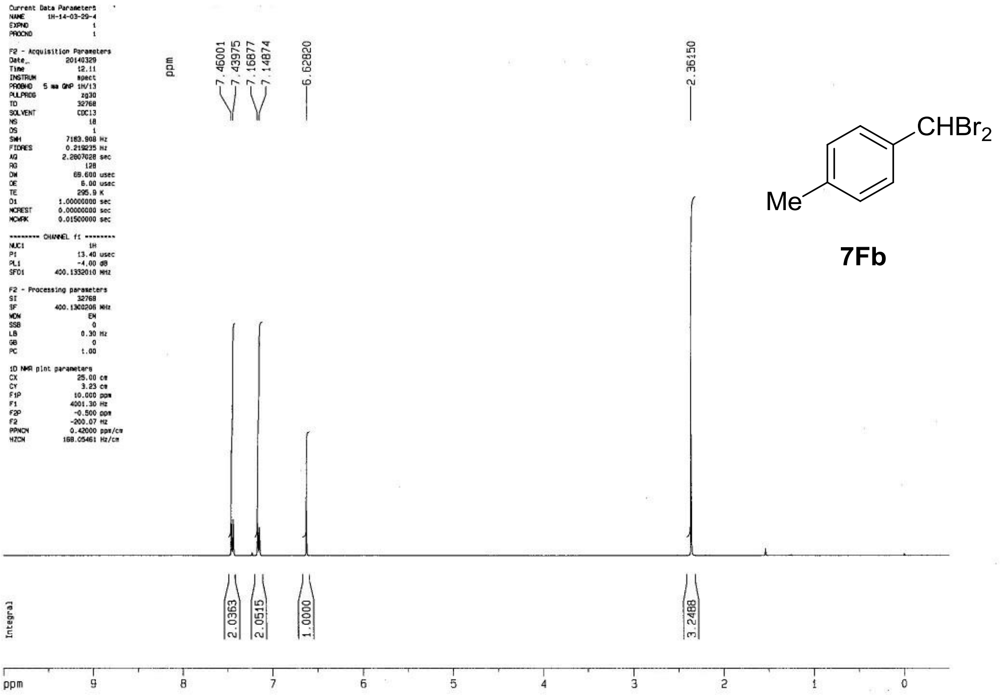

ppm
7.46001
7.43975
7.16877
7.14874
6.62820
2.36150
CHBr2
Me
7Fb
Integral
2.0363
2.0515
1.0000
3.2488
ppm 9 8 7 6 5 4 3 2 1 0

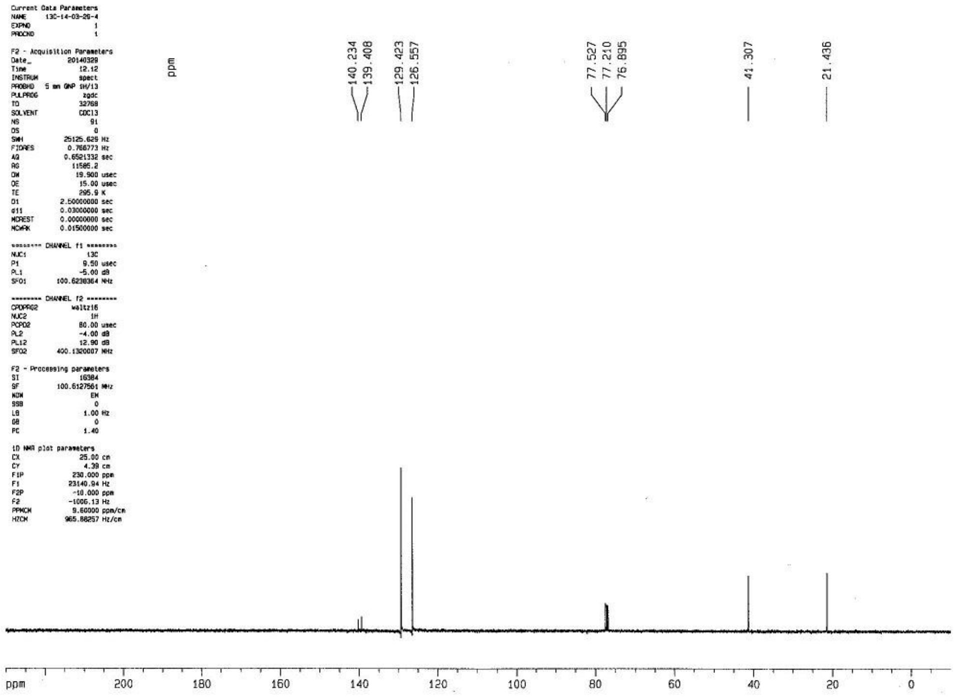

ppm
140.234
139.408
129.423
126.557
77.527
77.210
76.895
41.307
21.436
ppm 200 180 160 140 120 100 80 60 40 20 0

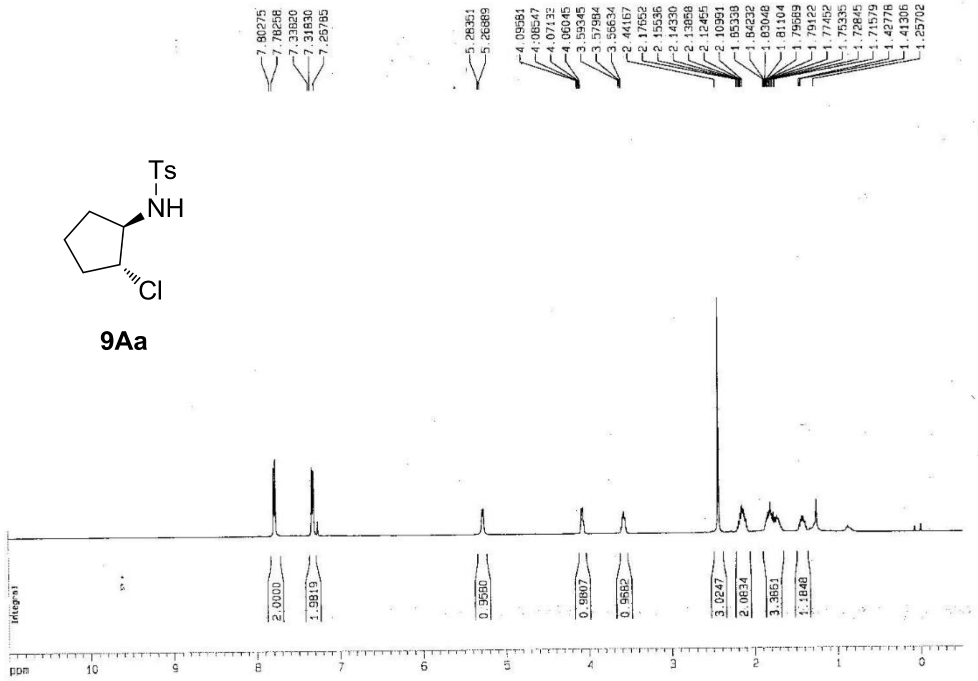

Ts
NH
Cl
7.80275
7.78258
7.33620
7.31830
7.25785
5.28351
5.26889
4.09581
4.08547
4.07132
4.06045
3.59345
3.57984
3.56634
2.44167
2.17652
2.15536
2.14330
2.13858
2.12455
2.10991
1.85338
1.84232
1.83048
1.81104
1.79589
1.79122
1.77452
1.75335
1.72845
1.71579
1.42778
1.41306
1.25702
Integral
2.0000
1.9819
0.9580
0.9807
0.9682
3.0247
2.0834
3.3861
1.1848
ppm
10
9
8
7
6
5
4
3
2
1
0

9Aa

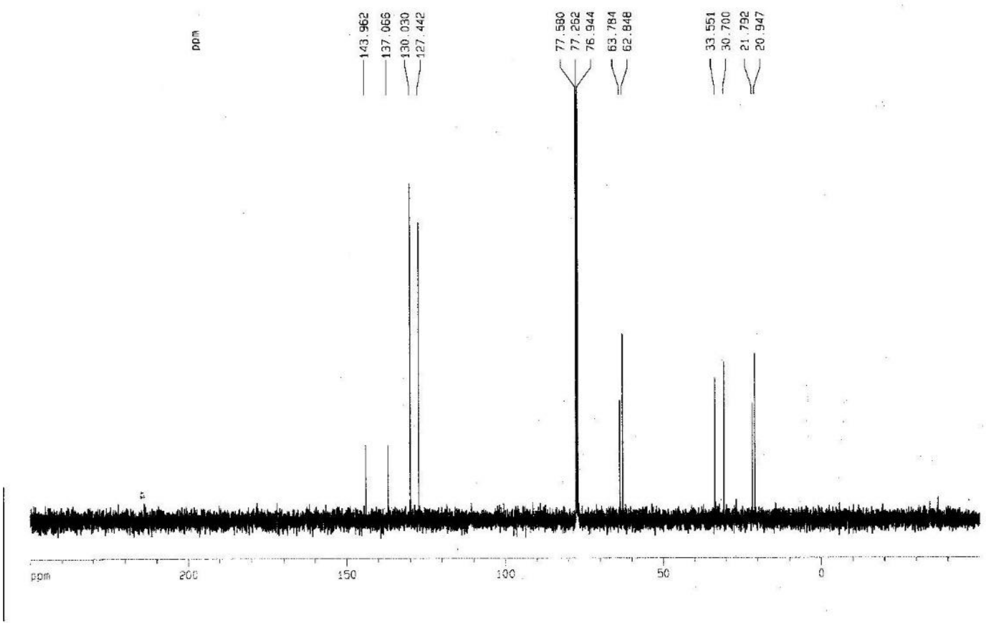

ppm
143.962
137.066
130.030
127.442
77.580
77.262
76.944
63.784
62.848
33.551
30.700
21.792
20.947
ppm
200
150
100
50
0

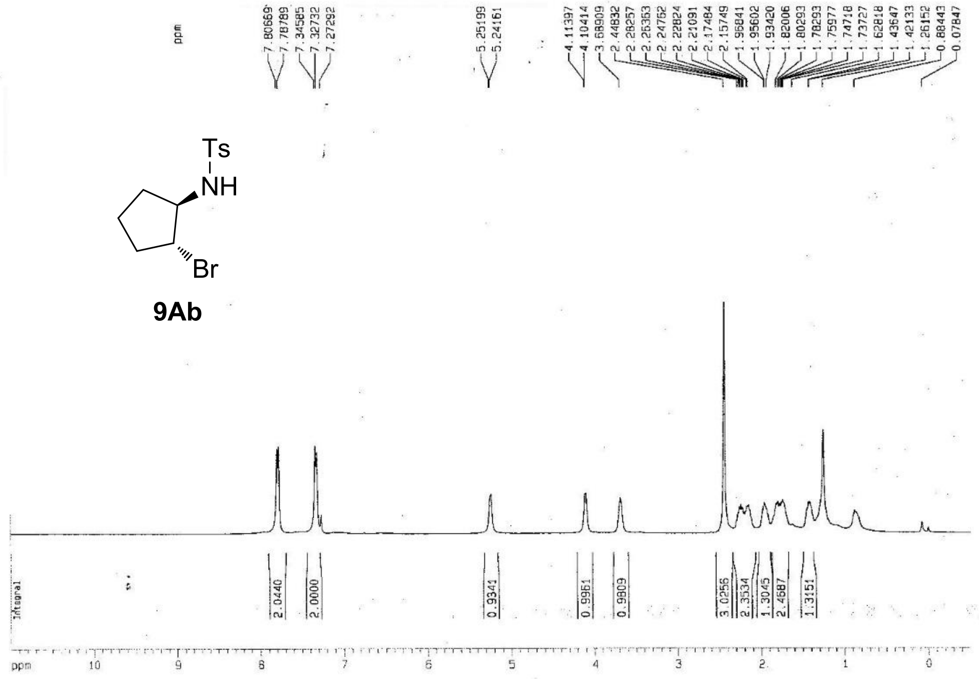
Ts
NH
Br
9Ab
ppm
7.80669
7.78789
7.34585
7.32732
7.27292
5.25199
5.24161
4.11397
4.10414
3.68909
2.44832
2.28257
2.26353
2.24752
2.22824
2.21091
2.17484
2.15749
1.96841
1.95602
1.93420
1.82006
1.80293
1.78293
1.75977
1.74718
1.73727
1.62818
1.43647
1.42133
1.26152
0.88443
0.07847
Integral
2.0440
2.0000
0.9341
0.9961
0.9809
3.0256
2.3534
1.3045
2.4687
1.3151
ppm 10 9 8 7 6 5 4 3 2 1 0

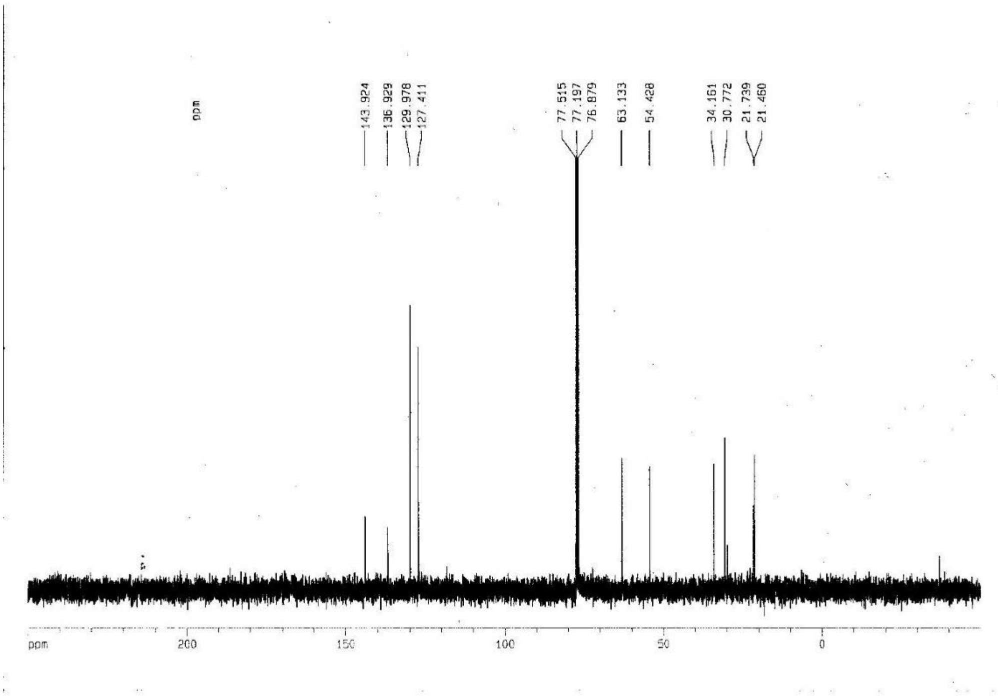
ppm
143.924
136.929
129.978
127.411
77.515
77.197
76.879
63.133
54.428
34.161
30.772
21.739
21.460
ppm 200 150 100 50 0

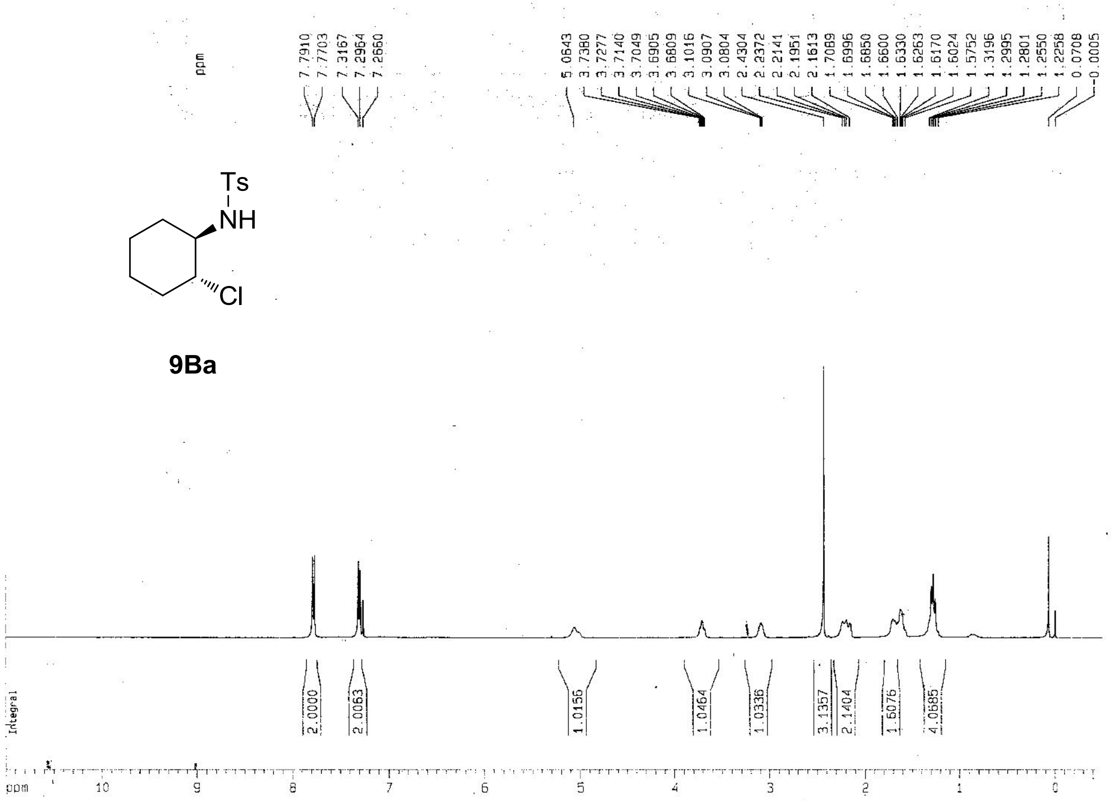
ppm
7.7910
7.7703
7.3167
7.2954
7.2660
5.0643
3.7380
3.7277
3.7140
3.7049
3.6905
3.6809
3.1016
3.0907
3.0804
2.4304
2.2372
2.2141
2.1951
2.1613
1.7089
1.6996
1.6850
1.6600
1.6330
1.6263
1.6170
1.6024
1.5752
1.3196
1.2995
1.2801
1.2550
1.2258
0.0708
-0.0005
Ts
NH
Cl
9Ba
Integral
2.0000
2.0063
1.0156
1.0464
1.0336
3.1357
2.1404
1.6076
4.0685
ppm
10
9
8
7
6
5
4
3
2
1
0

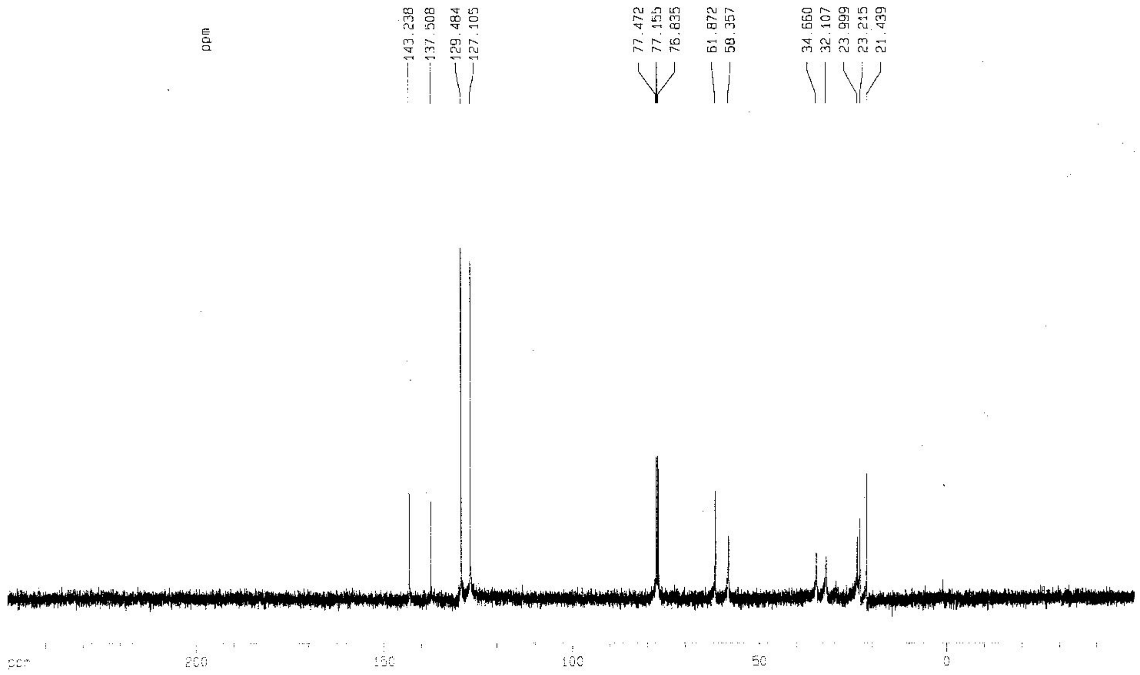
ppm
143.238
137.508
129.484
127.105
77.472
77.155
76.835
61.872
58.357
34.660
32.107
23.999
23.215
21.439
200
150
100
50
0

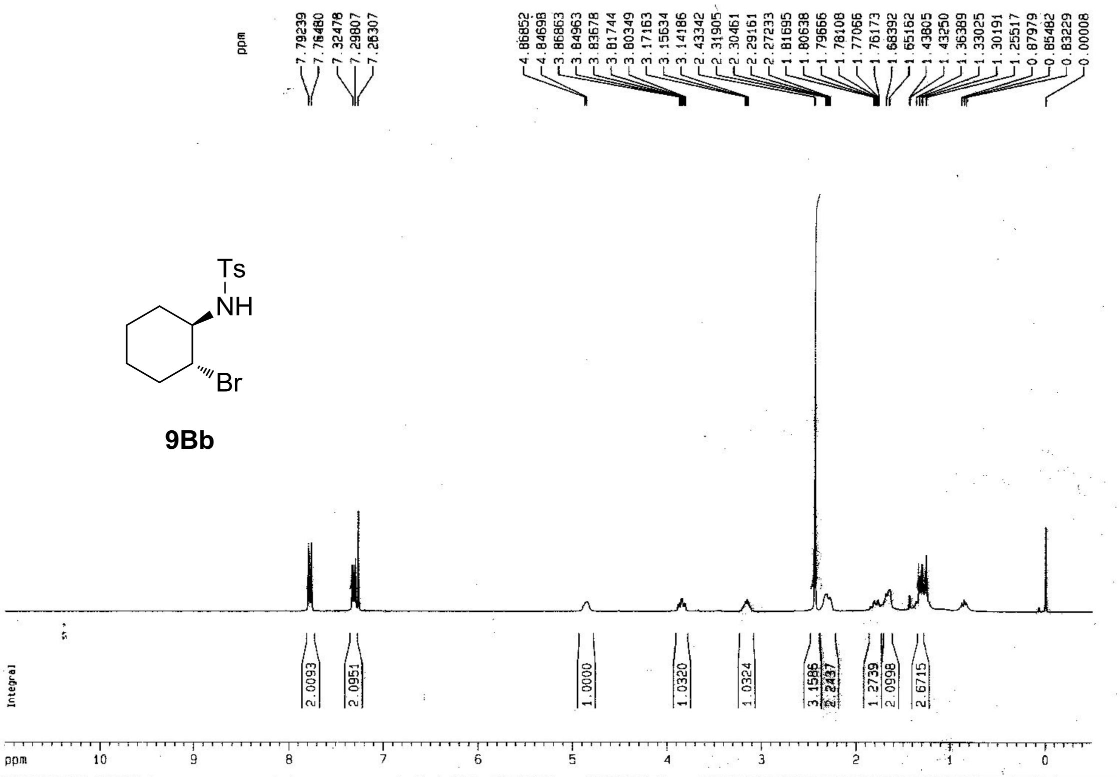
ppm
7.79239
7.76480
7.32478
7.29807
7.26307
4.86852
4.84698
3.86863
3.84963
3.83678
3.81744
3.80349
3.17163
3.15634
3.14186
2.43342
2.31905
2.30461
2.29161
2.27233
1.81695
1.80638
1.79666
1.78108
1.77066
1.76173
1.68392
1.65162
1.43805
1.43250
1.36389
1.33025
1.30191
1.25517
0.87979
0.85482
0.83229
0.00008
Integral
2.0093
2.0951
1.0000
1.0320
1.0324
3.1586
2.2437
1.2739
2.0998
2.6715
ppm
10
9
8
7
6
5
4
3
2
1
0
Ts
NH
Br

9Bb

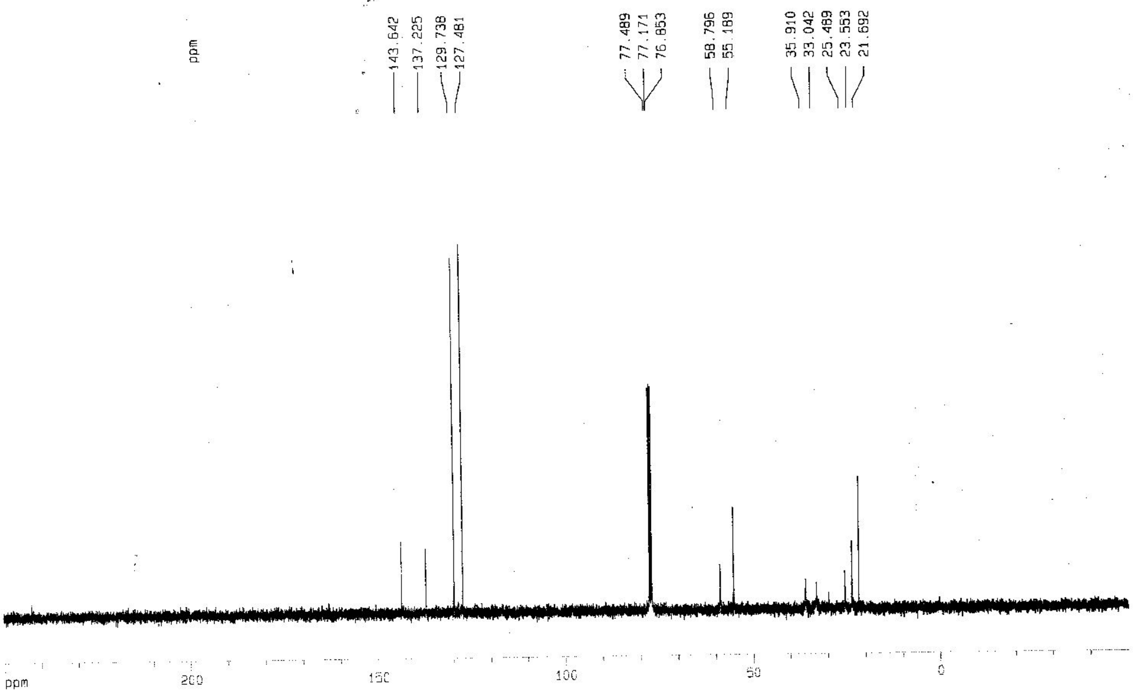
ppm
143.642
137.225
129.738
127.481
77.489
77.171
76.853
58.796
55.189
35.910
33.042
25.489
23.553
21.692
ppm
200
150
100
50
0

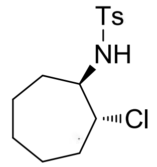
Ts
NH
Cl

9Ca

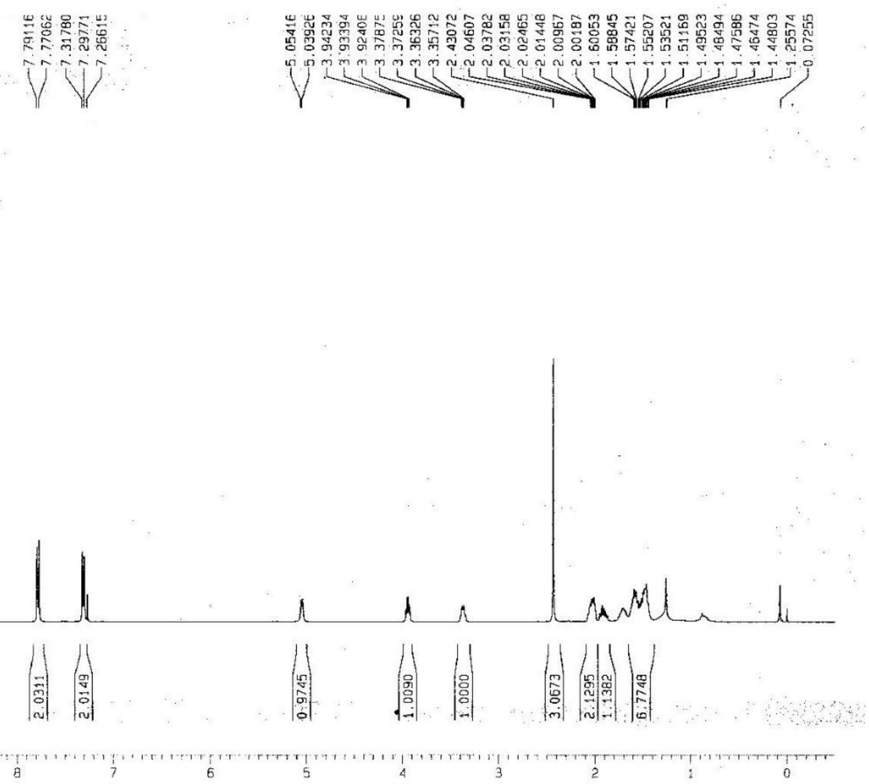
ppm
7.79116
7.77062
7.31780
7.29771
7.26615
5.05416
5.03926
3.94234
3.93394
3.92408
3.37875
3.37259
3.36326
3.35712
2.43072
2.04607
2.03782
2.03158
2.02465
2.01448
2.00967
2.00187
1.60053
1.58845
1.57421
1.55207
1.53521
1.51169
1.49523
1.48494
1.47586
1.46474
1.44803
1.25574
0.07255
Integral
2.0311
2.0149
0.9745
1.0090
1.0000
3.0673
2.1295
1.1382
6.7748
ppm
10
9
8
7
6
5
4
3
2
1
0

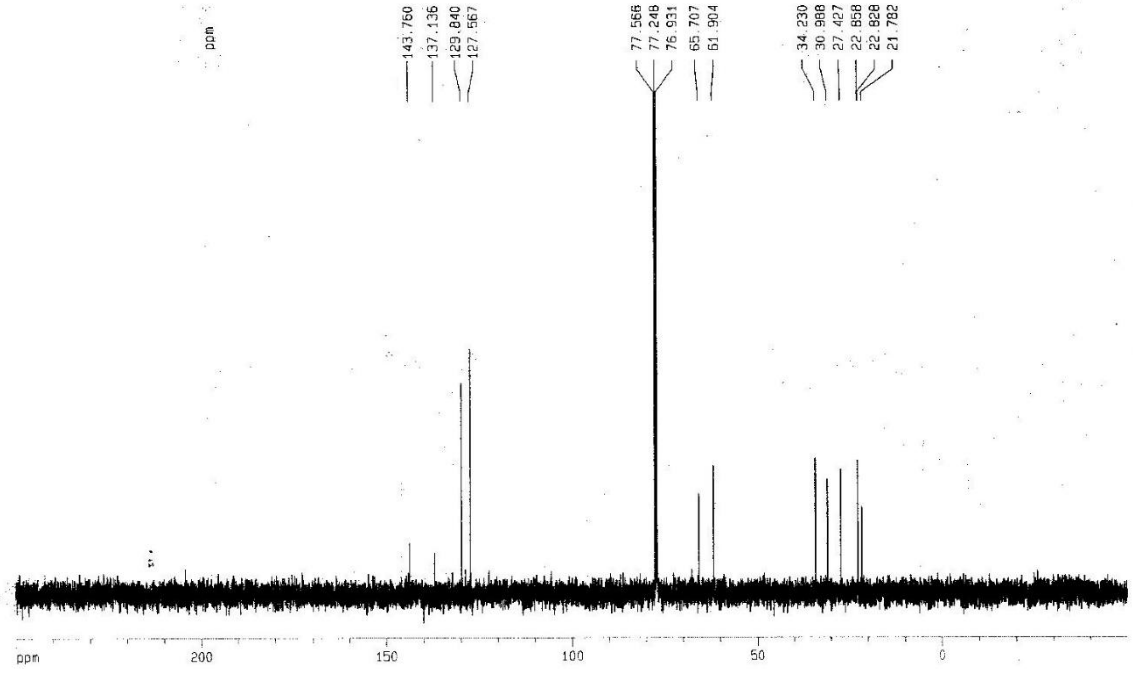
ppm
143.760
137.136
129.840
127.567
77.566
77.248
76.931
65.707
61.904
34.230
30.988
27.427
22.858
22.828
21.782
ppm
200
150
100
50
0

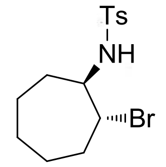
Ts
NH
Br

9Cb

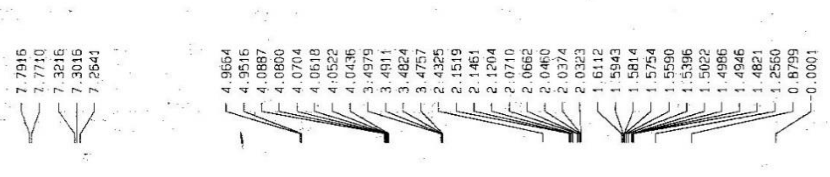
ppm
7.7916
7.7710
7.3216
7.3016
7.2641
4.9664
4.9516
4.0887
4.0800
4.0704
4.0618
4.0522
4.0436
3.4979
3.4911
3.4824
3.4757
2.4325
2.1519
2.1461
2.1204
2.0710
2.0662
2.0460
2.0374
2.0323
1.6112
1.5943
1.5814
1.5754
1.5590
1.5396
1.5022
1.4986
1.4946
1.4821
1.2550
0.8799
-0.0001

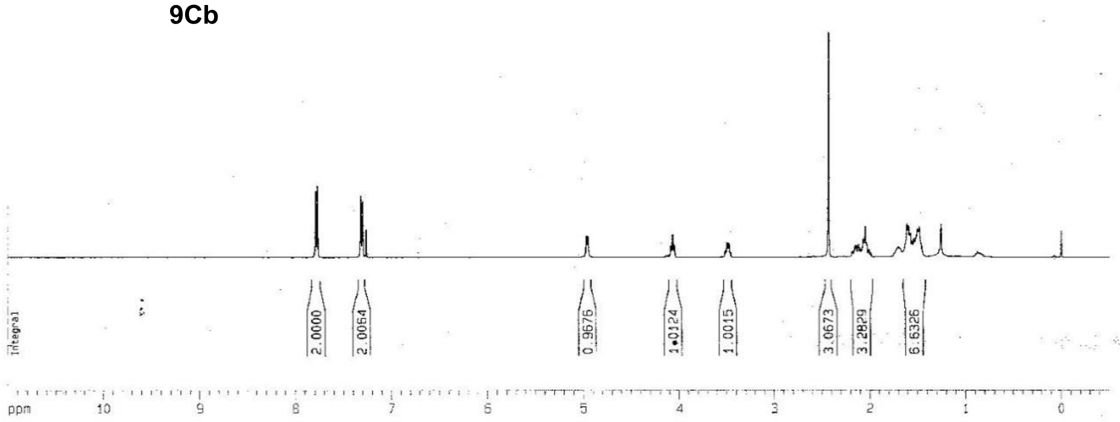
Integral
2.0000
2.0064
0.9676
1.0124
1.0015
3.0673
3.2829
6.6326
ppm
10
9
8
7
6
5
4
3
2
1
0

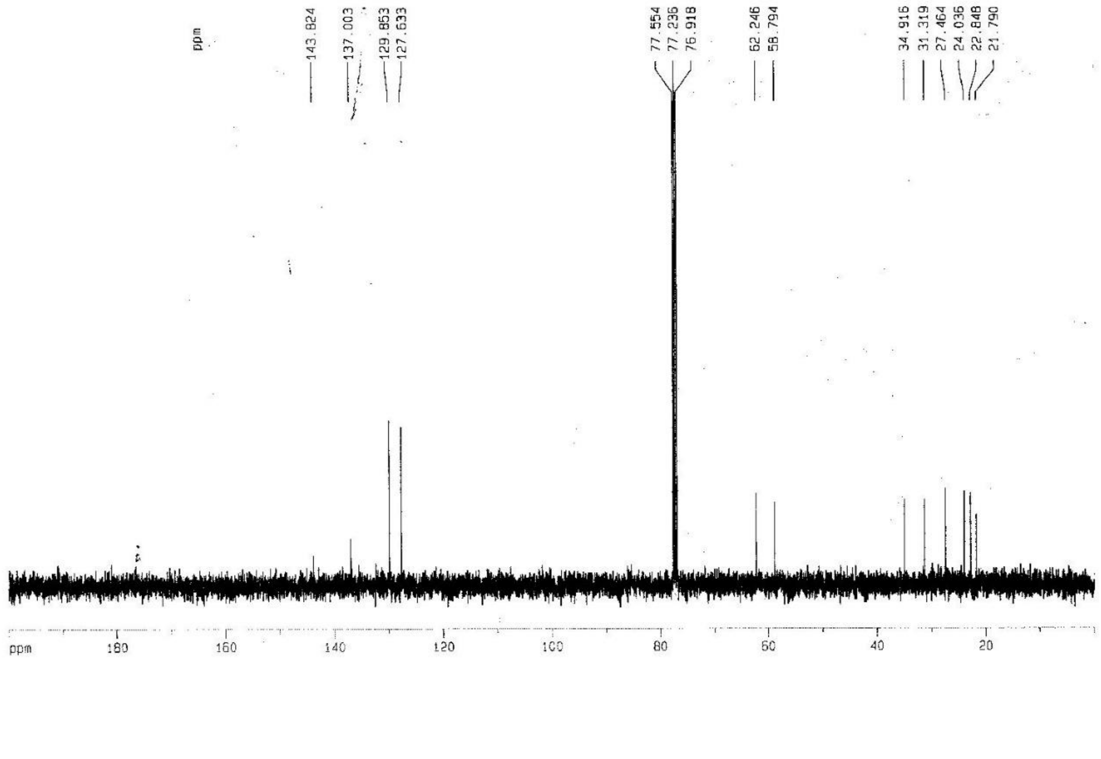
ppm
143.824
137.003
129.853
127.633
77.554
77.236
76.918
62.246
58.794
34.916
31.319
27.464
24.036
22.848
21.790
ppm
180
160
140
120
100
80
60
40
20

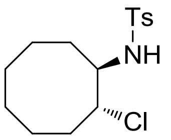
Ts
NH
Cl

9Da

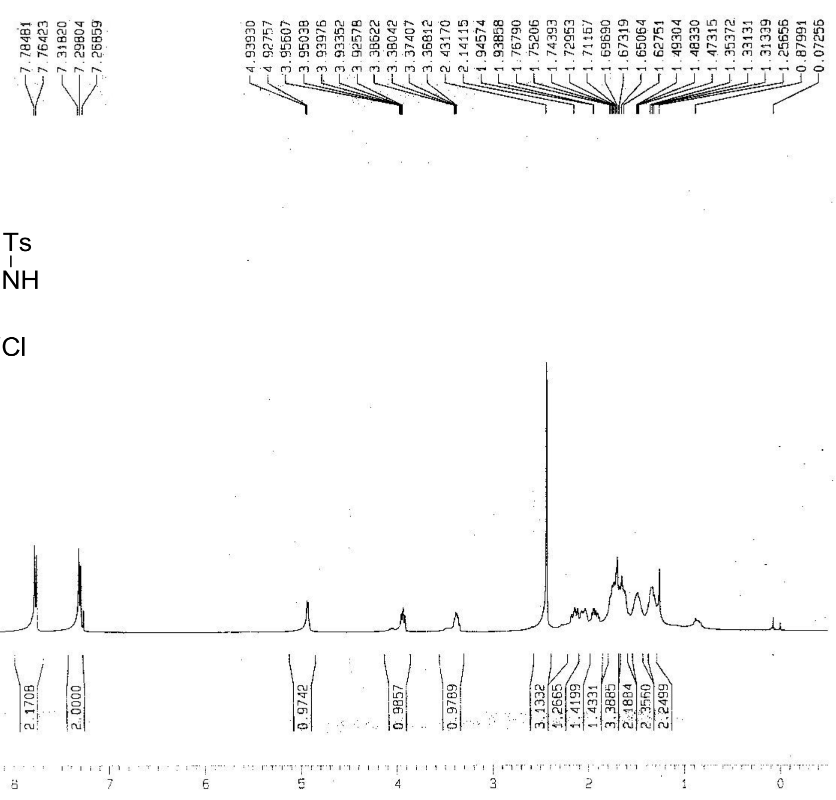
ppm
7.78481
7.76423
7.31820
7.29804
7.26859
4.93930
4.92757
3.95607
3.95038
3.93976
3.93352
3.92578
3.38622
3.38042
3.37407
3.36812
2.43170
2.14115
1.94574
1.93858
1.76790
1.75206
1.74393
1.72953
1.71167
1.69690
1.67319
1.65064
1.62751
1.49304
1.48330
1.47315
1.35372
1.33131
1.31339
1.25656
0.87991
0.07256
Integral
2.1708
2.0000
0.9742
0.9857
0.9789
3.1332
1.2665
1.4199
1.4331
3.3885
2.1884
2.3560
2.2499
ppm 10 9 8 7 6 5 4 3 2 1 0

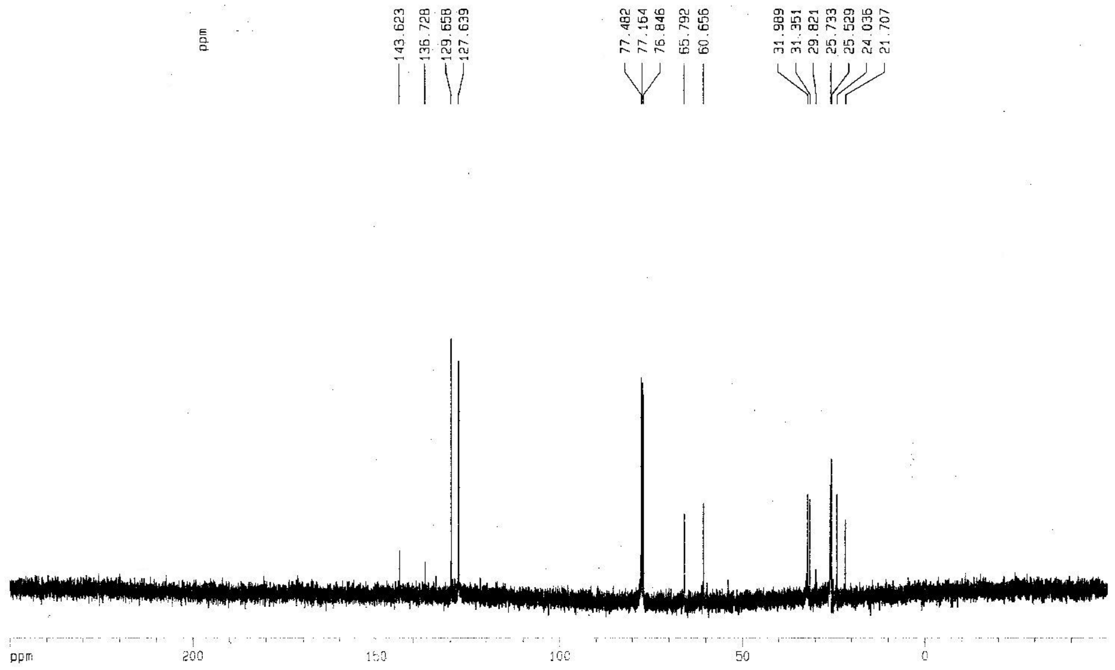
ppm
143.623
136.728
129.658
127.639
77.482
77.164
76.846
65.792
60.656
31.989
31.351
29.821
25.733
25.529
24.036
21.707
ppm 200 150 100 50 0

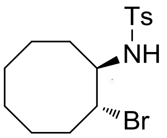

9Db

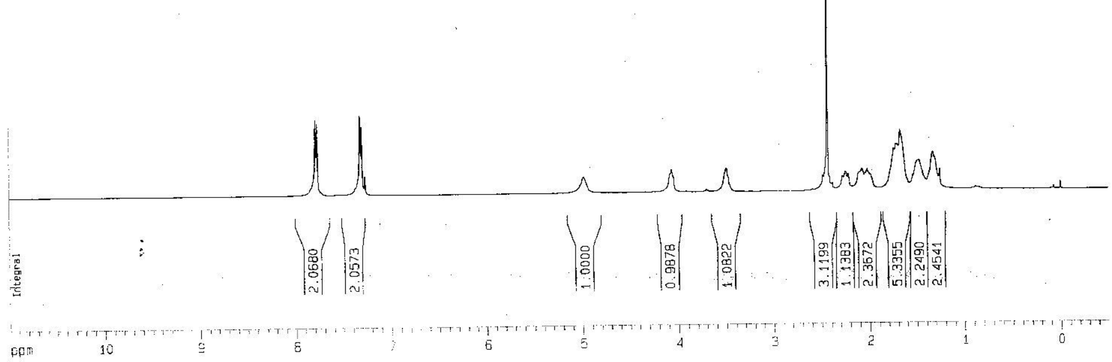
7.79345
7.77387
7.32240
7.30308
7.26898
4.98635
4.08181
4.06408
4.04657
3.49764
2.48281
2.43330
2.38316
2.27725
2.24744
2.21894
2.11210
2.07667
2.04600
2.02556
2.00529
1.98717
1.74161
1.71750
1.70286
1.67489
1.47737
1.33130
1.31083
1.25628
0.00014
2.0680
2.0573
1.0000
0.9878
1.0822
3.1199
1.1383
2.3672
5.3355
2.2490
2.4541
Integral
ppm

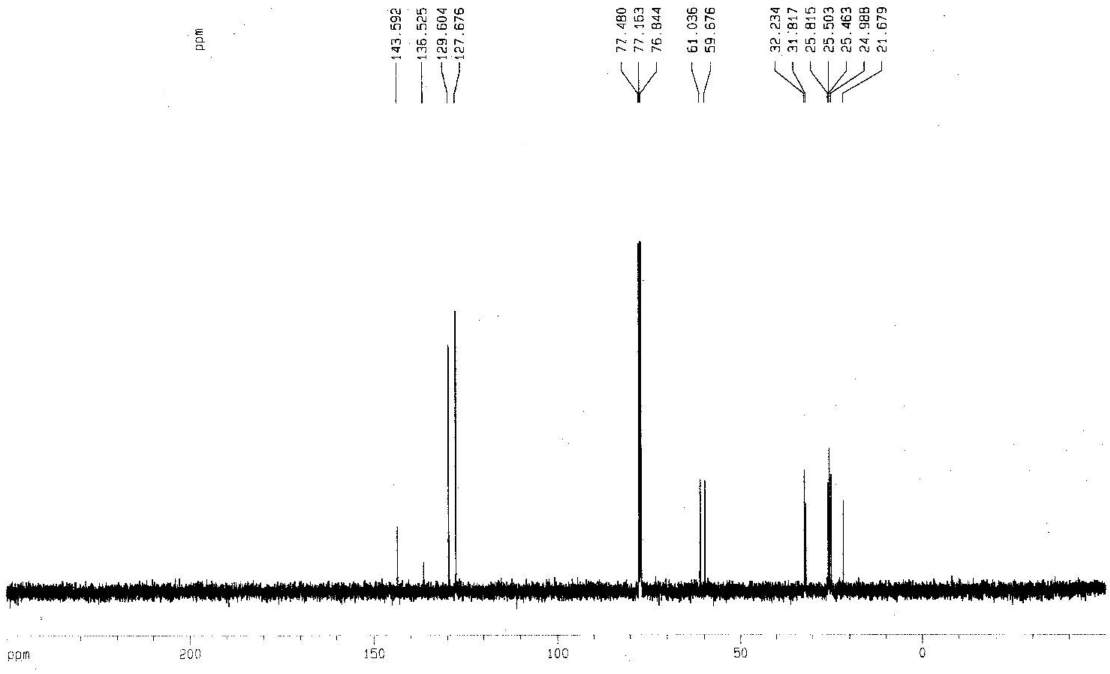
143.592
136.525
129.604
127.676
77.480
77.163
76.844
61.036
59.676
32.234
31.817
25.815
25.503
25.463
24.988
21.679
ppm

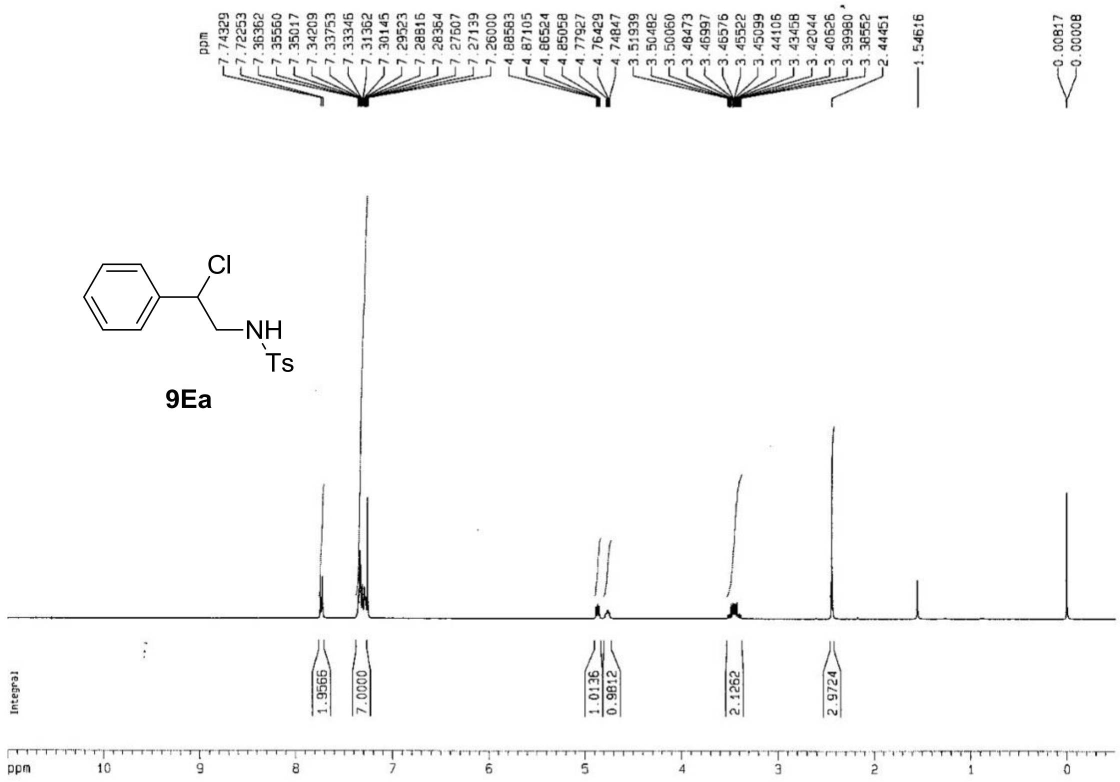
ppm
7.74329
7.72253
7.36362
7.35560
7.35017
7.34209
7.33753
7.33346
7.31362
7.30145
7.29523
7.28816
7.28364
7.27607
7.27139
7.26000
4.88583
4.87105
4.86524
4.85058
4.77927
4.76429
4.74847
3.51939
3.50482
3.50060
3.48473
3.46997
3.46576
3.45522
3.45099
3.44106
3.43458
3.42044
3.40526
3.39980
3.38552
2.44451
1.54616
0.00817
0.00008
Cl
NH
Ts
9Ea
Integral
1.9566
7.0000
1.0136
0.9812
2.1262
2.9724
ppm 10 9 8 7 6 5 4 3 2 1 0

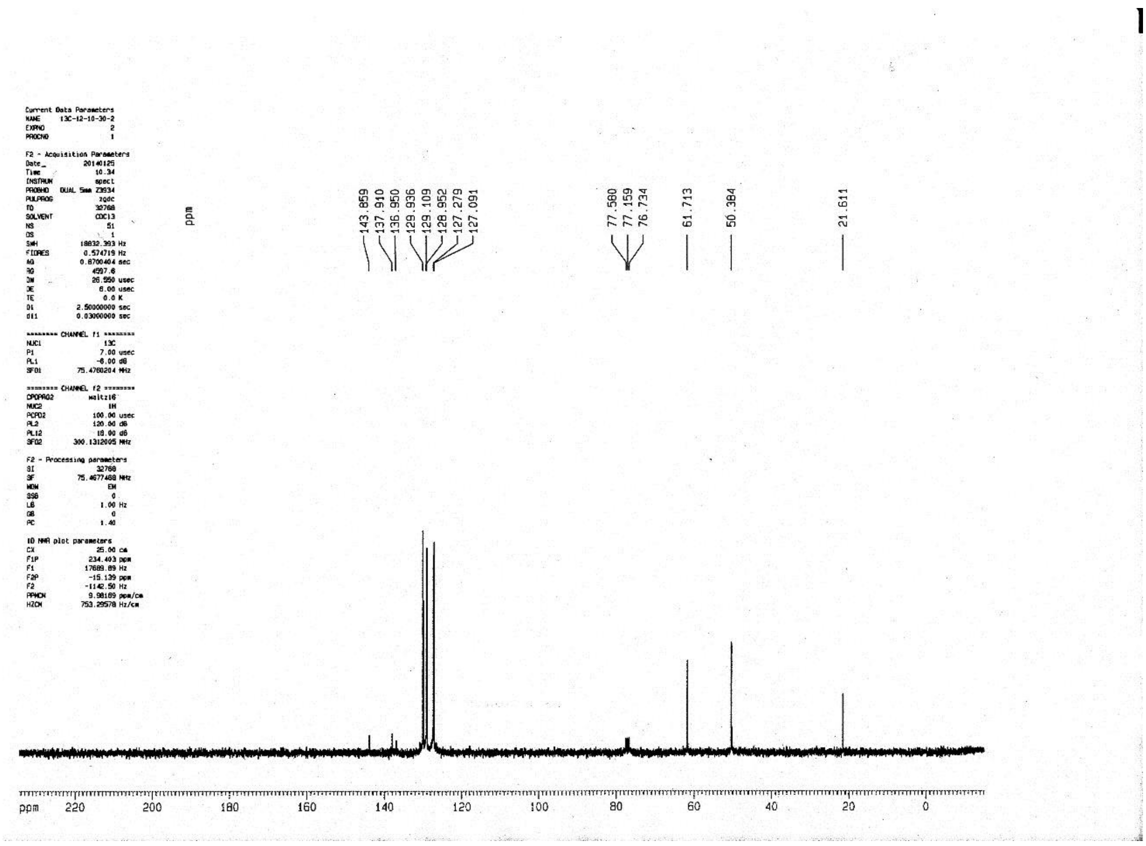
ppm
143.859
137.910
136.950
129.936
129.109
128.952
127.279
127.091
77.580
77.159
76.734
61.713
50.384
21.611
ppm 220 200 180 160 140 120 100 80 60 40 20 0

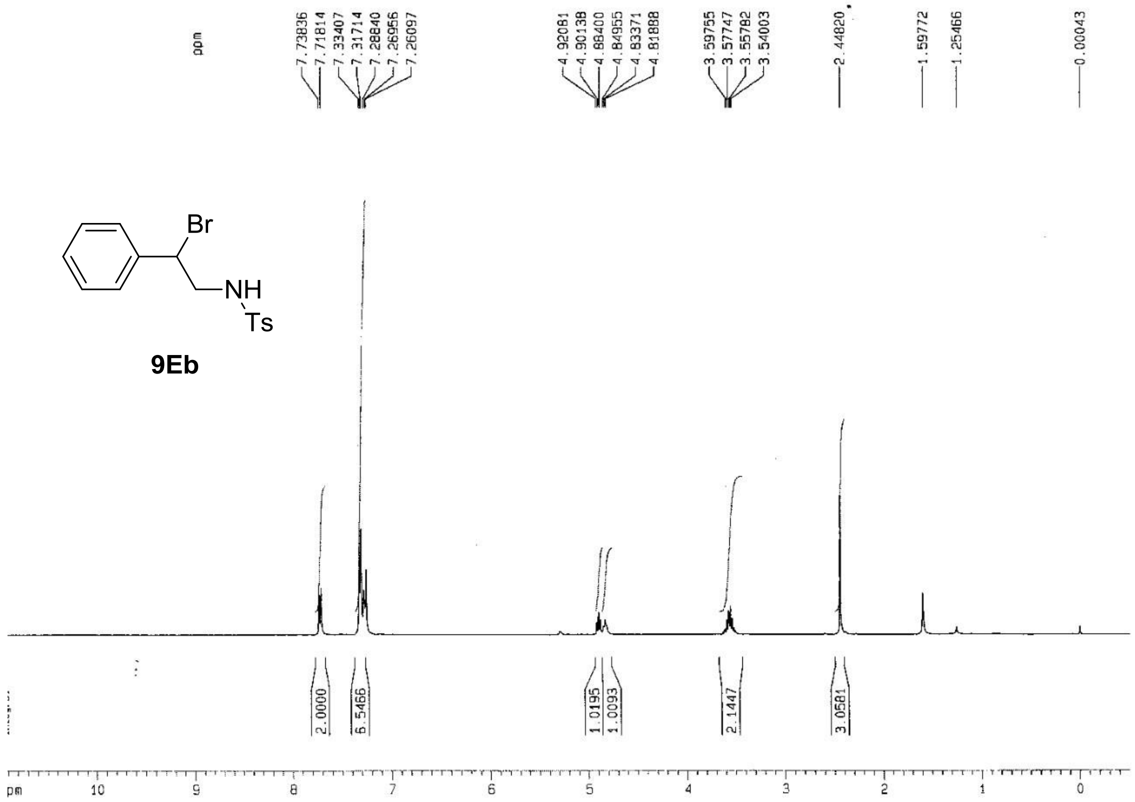
Br
NH
Ts
9Eb
ppm
7.73836
7.71814
7.33407
7.31714
7.28840
7.26956
7.26097
4.92081
4.90138
4.88400
4.84955
4.83371
4.81888
3.59755
3.57747
3.55782
3.54003
2.44820
1.59772
1.25466
0.00043
2.0000
6.5466
1.0195
1.0093
2.1447
3.0581
ppm 10 9 8 7 6 5 4 3 2 1 0

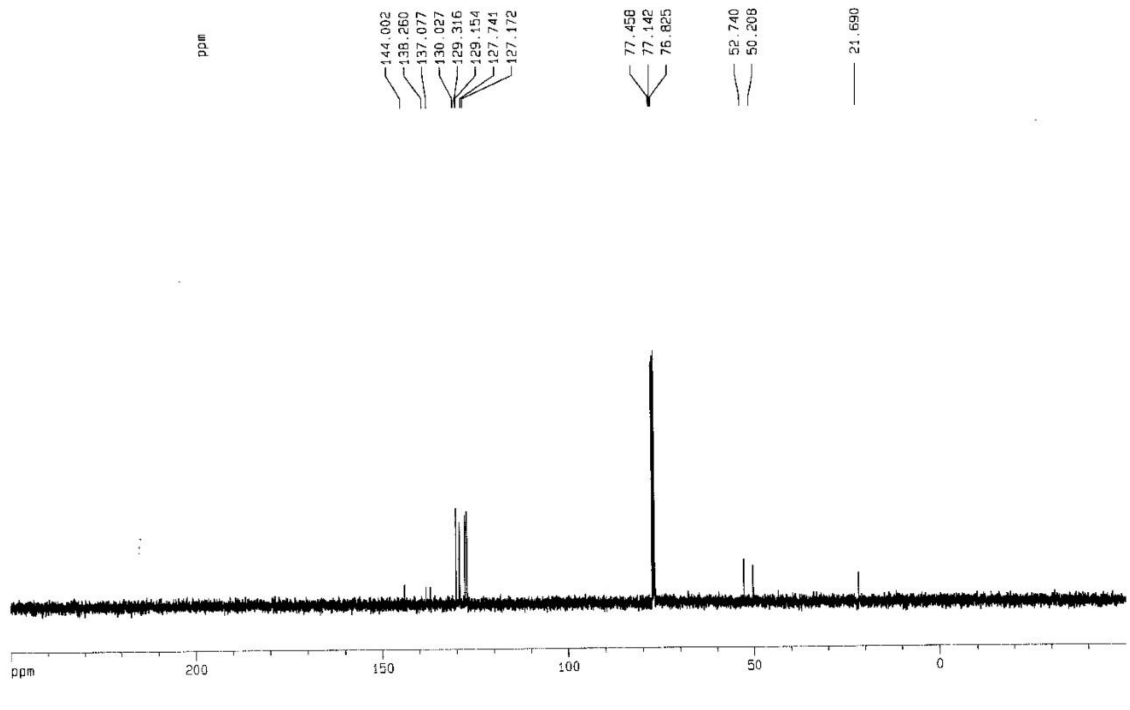
ppm
144.002
138.260
137.077
130.027
129.316
129.154
127.741
127.172
77.458
77.142
76.825
52.740
50.208
21.690
ppm 200 150 100 50 0

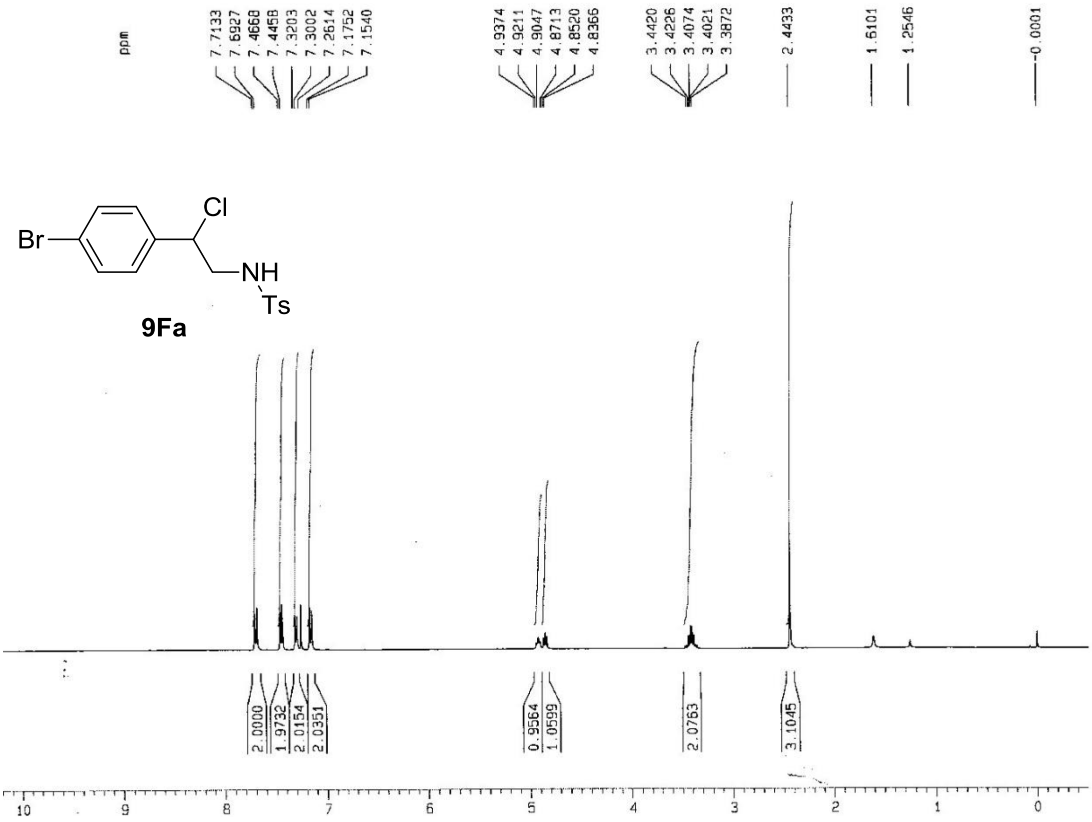

9Fa

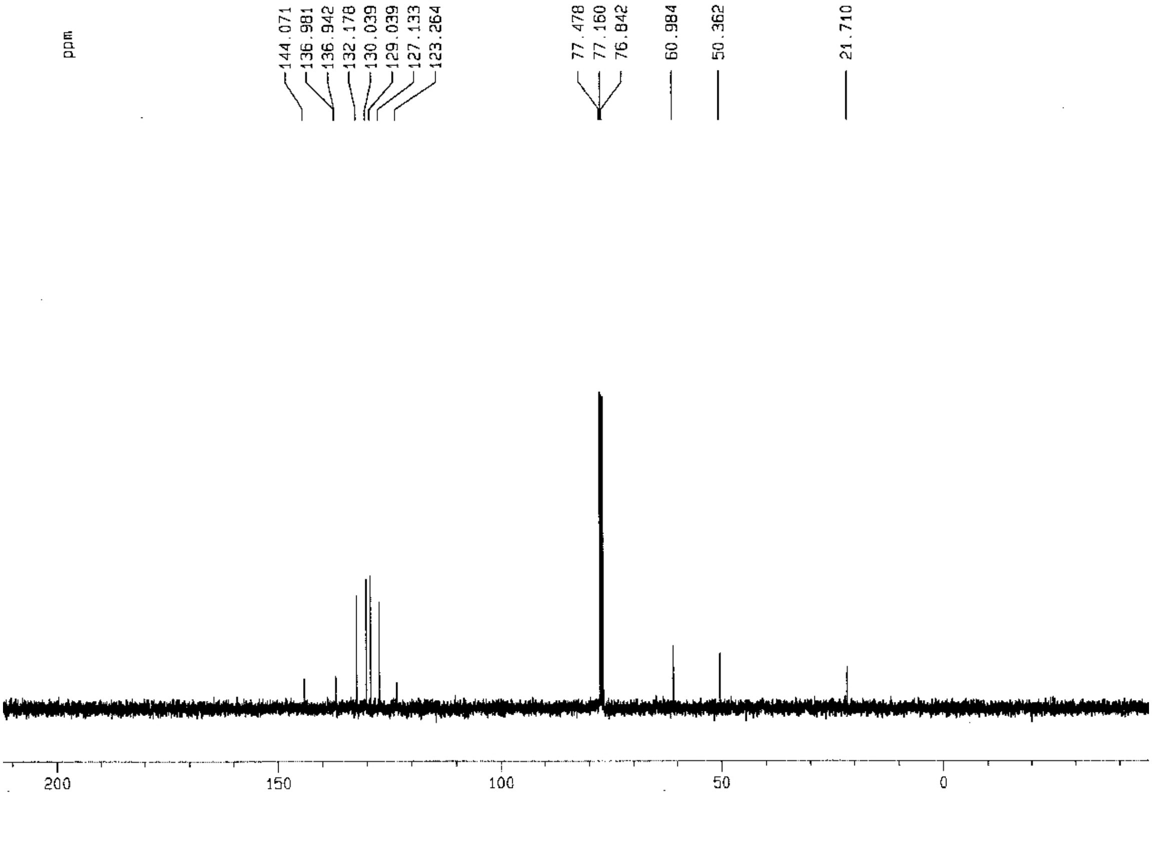

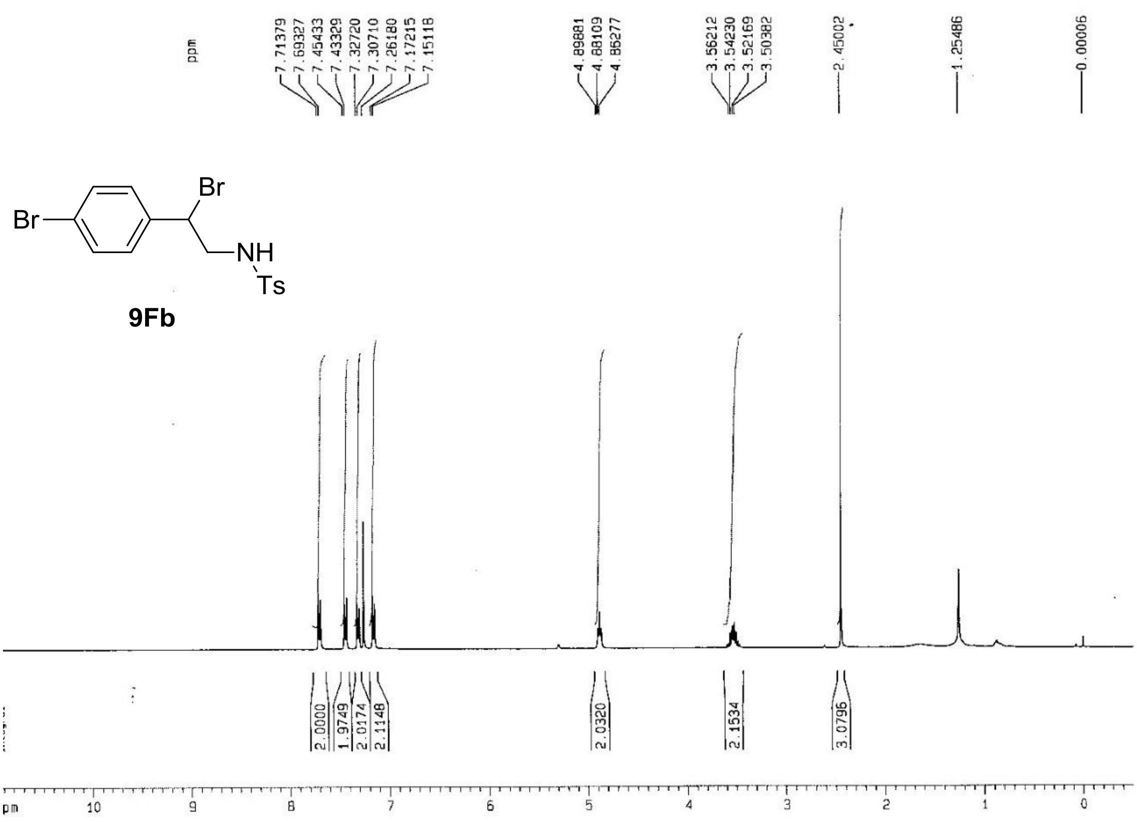
Br
Br
NH
Ts

9Fb

ppm
7.71379
7.69327
7.45433
7.43329
7.32720
7.30710
7.26180
7.17215
7.15118
4.89881
4.88109
4.86277
3.56212
3.54230
3.52169
3.50382
2.45002
1.25486
0.00006
2.0000
1.9749
2.0174
2.1148
2.0320
2.1534
3.0796
ppm
10
9
8
7
6
5
4
3
2
1
0

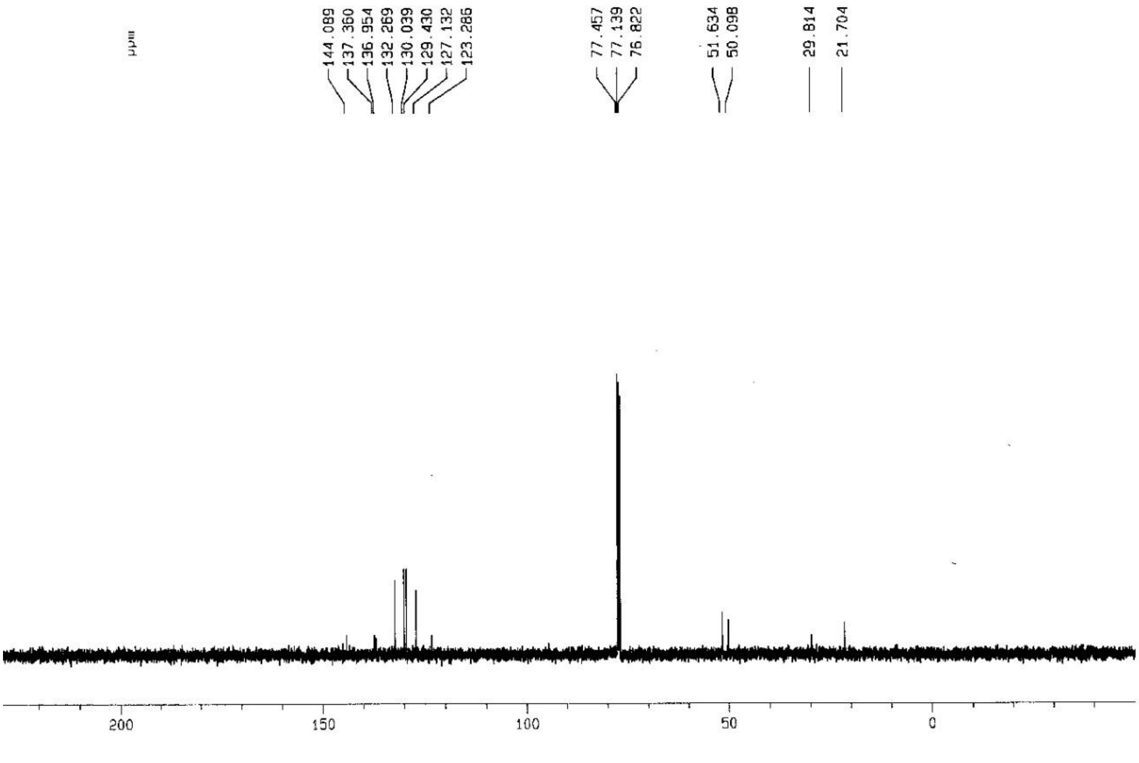
ppm
144.089
137.360
136.954
132.269
130.039
129.430
127.132
123.286
77.457
77.139
76.822
51.634
50.098
29.814
21.704
200
150
100
50
0

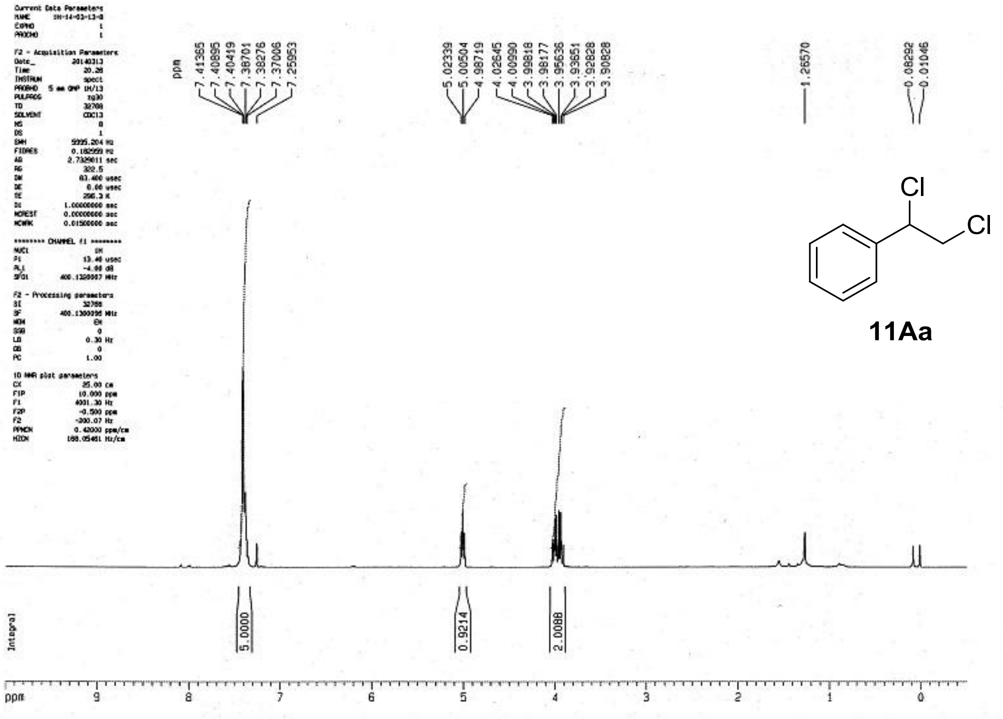
ppm
7.41365
7.40895
7.40419
7.38701
7.38276
7.37006
7.25953
5.02339
5.00504
4.98719
4.02645
4.00990
3.99818
3.98177
3.95636
3.93651
3.92828
3.90828
1.26570
0.08292
0.01046
Cl
Cl
11Aa
Integral
5.0000
0.9214
2.0088
ppm
9
8
7
6
5
4
3
2
1
0

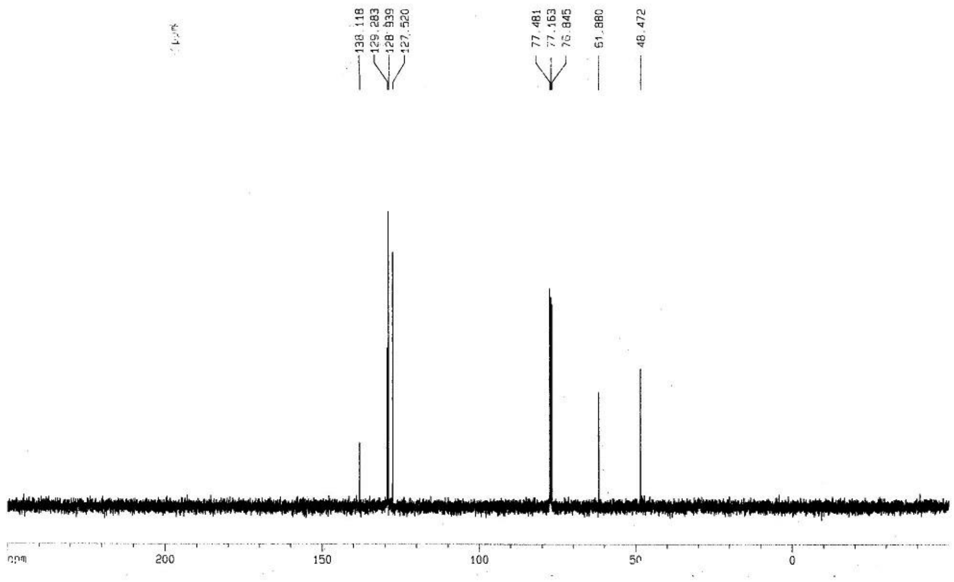
138.118
129.283
128.939
127.520
77.481
77.163
76.845
61.880
48.472
ppm
200
150
100
50
0

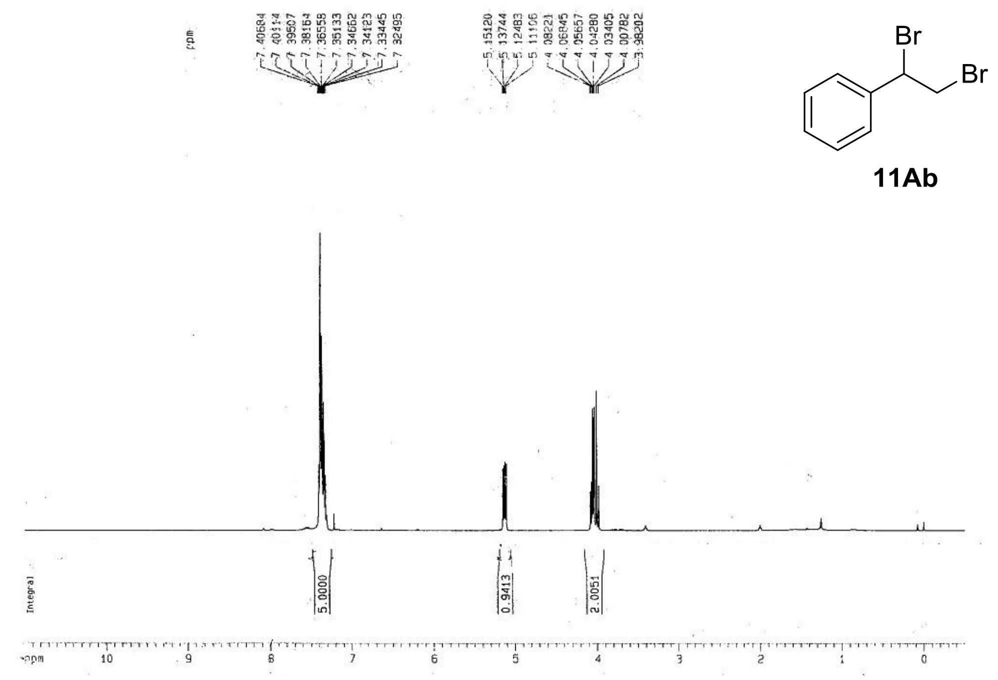
7.40684
7.40314
7.39507
7.38164
7.36558
7.35133
7.34662
7.34123
7.33445
7.32495
5.15120
5.13744
5.12483
5.11106
4.08221
4.06845
4.05657
4.04280
4.03405
4.00782
3.98202
5.0000
0.9413
2.0051
Integral
ppm
Br
Br

11Ab

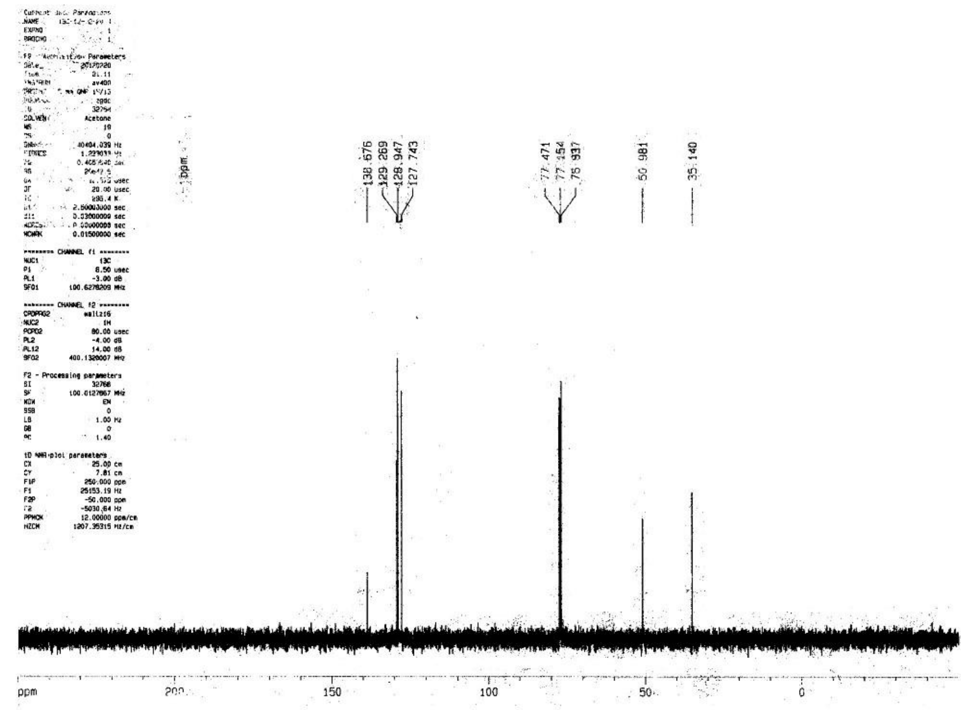
138.676
129.269
128.947
127.743
77.471
77.154
76.937
50.981
35.140
ppm

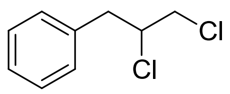
Cl
Cl

11Ba

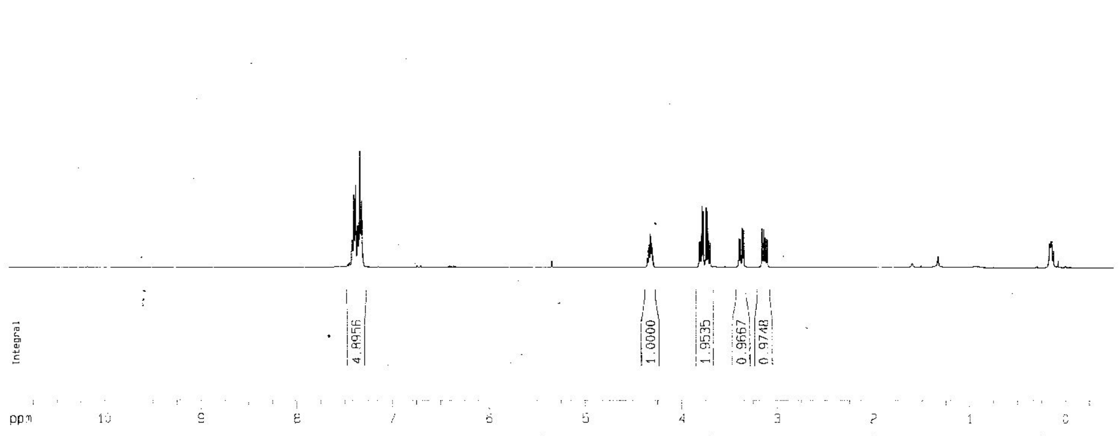
ppm
7.42284
7.41901
7.40249
7.38394
7.36167
7.35847
7.34156
7.33744
7.33056
7.32124
7.30230
5.34221
4.34008
4.33693
4.32358
4.31039
4.29303
3.80870
3.79675
3.78008
3.76814
3.73984
3.72273
3.71121
3.69411
3.39575
3.38150
3.36023
3.34599
3.15463
3.13637
3.11911
3.10086
1.32852
0.16334
0.14901
0.12317
Integral
4.8956
1.0000
1.9535
0.9667
0.9748
ppm

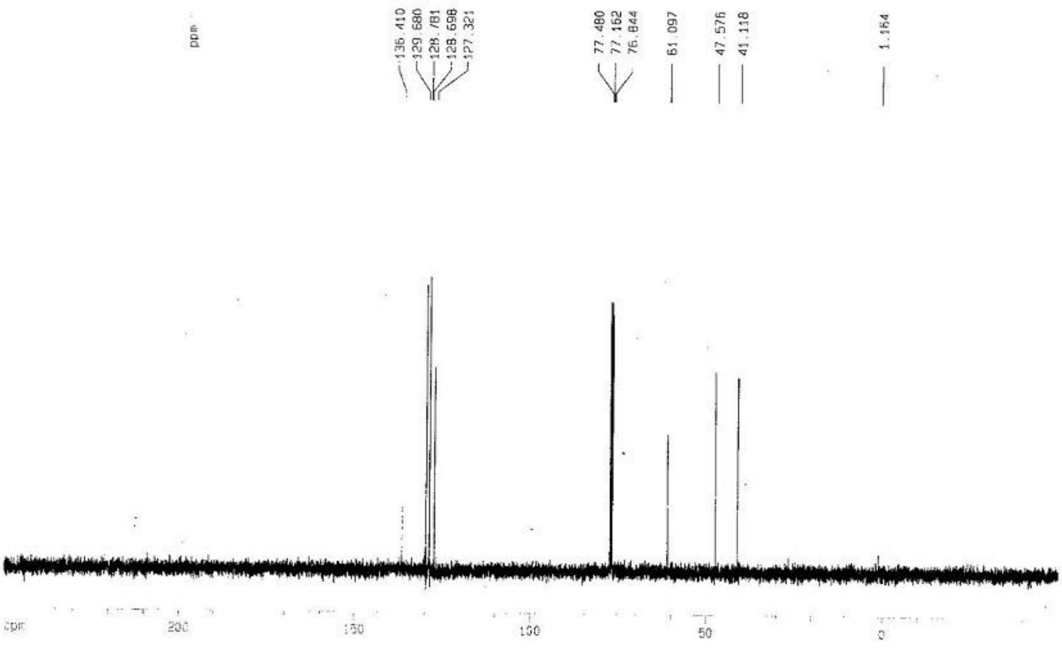
ppm
136.410
129.680
128.781
128.698
127.321
77.480
77.162
76.844
61.097
47.576
41.118
1.164
200
150
100
50
0

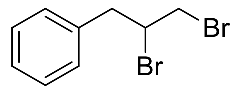
Br
Br

11Bb

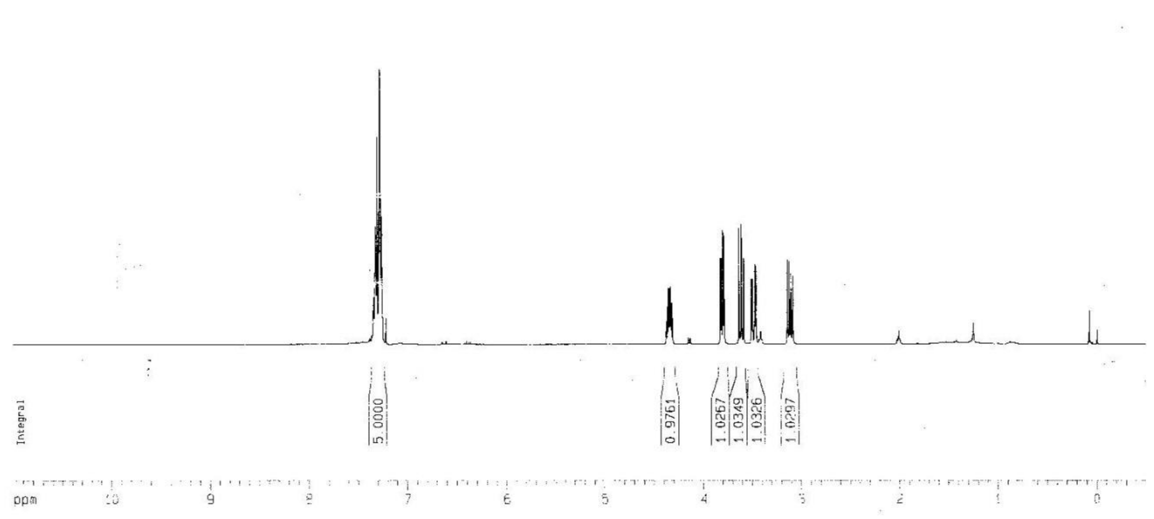
ppm
1.25360
0.07514
0.00002
Integral
5.0000
0.9761
1.0267
1.0349
1.0326
1.0297
ppm
10
9
8
7
6
5
4
3
2
1
0

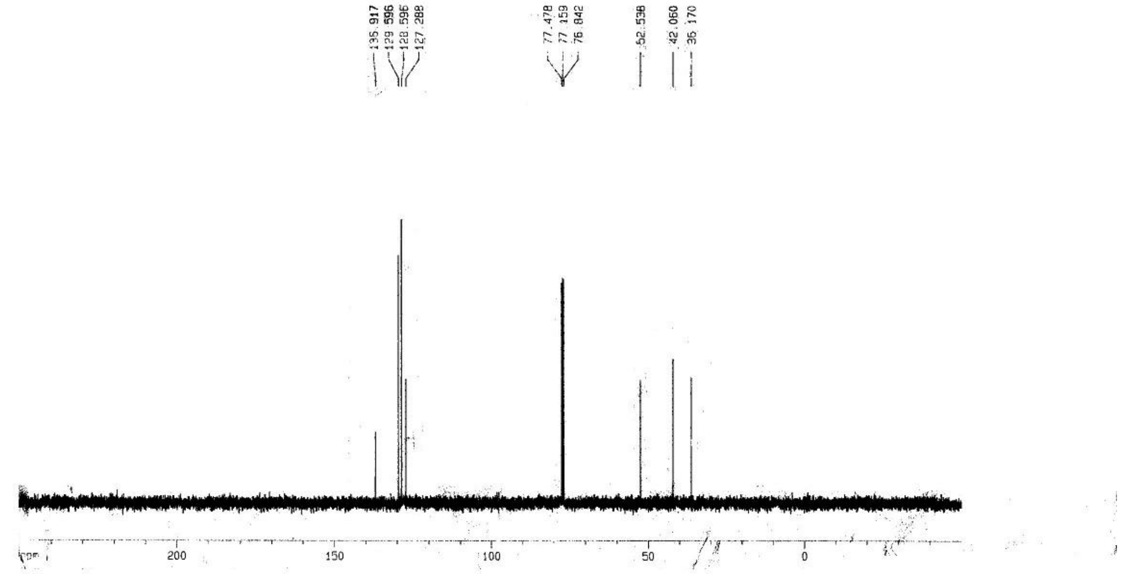
135.917
129.596
128.596
127.288
77.478
77.159
76.842
52.538
42.060
36.170
ppm
200
150
100
50
0

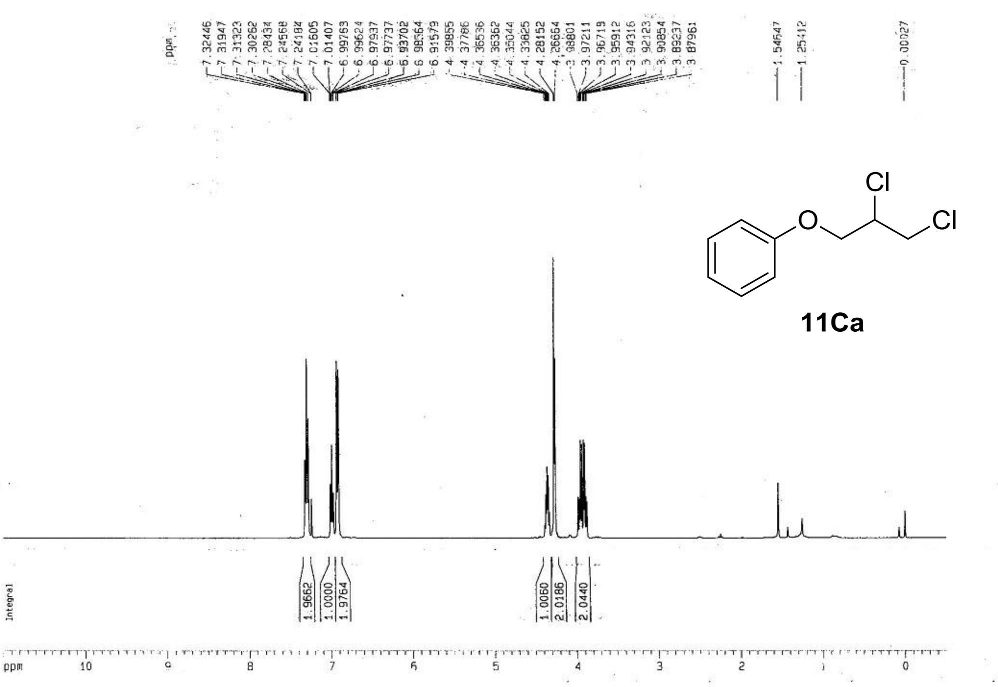

1.54547
1.25412
Integral
1.9662
1.0000
1.9764
1.0060
2.0186
2.0440
ppm
Cl
O
Cl

11Ca

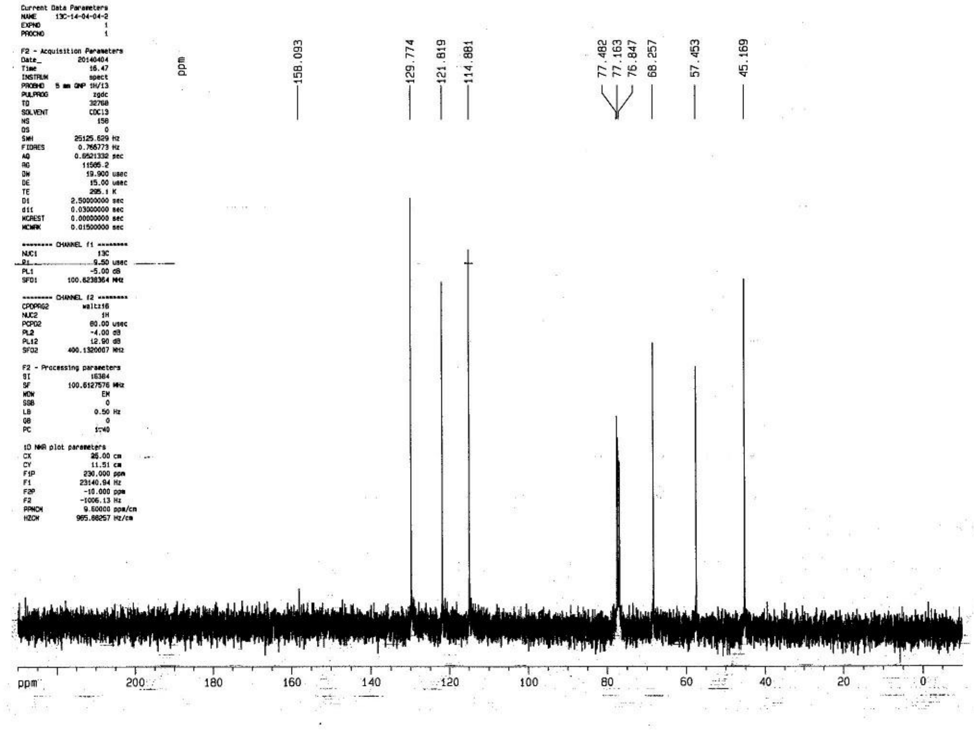

158.093
129.774
121.819
114.881
77.482
77.163
76.847
68.257
57.453
45.169
ppm

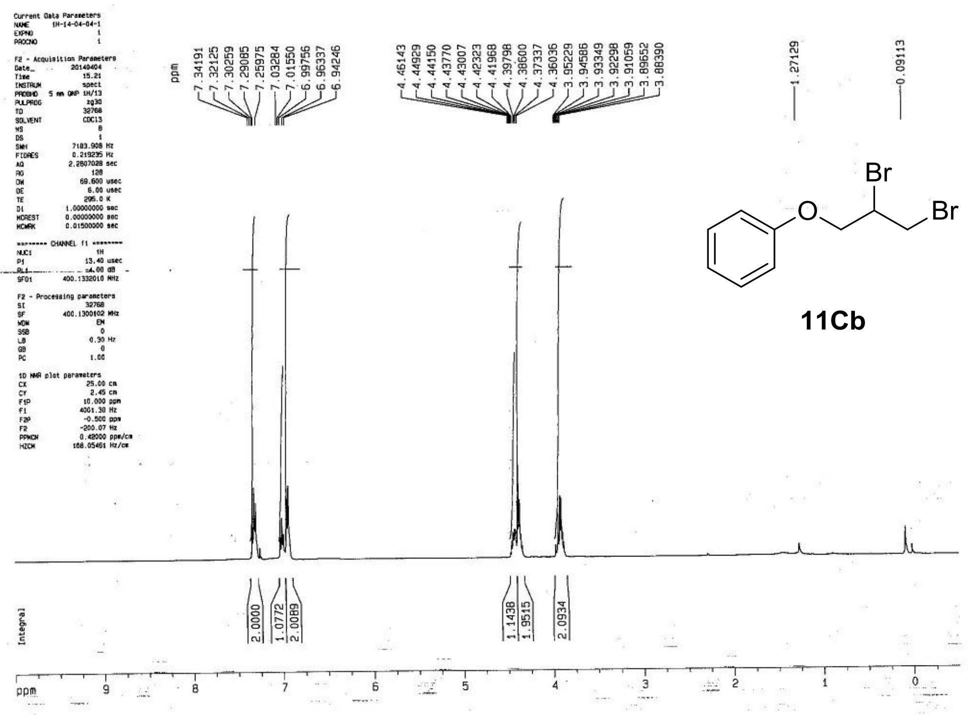
7.34191
7.32125
7.30259
7.29085
7.25975
7.03284
7.01550
6.99756
6.96337
6.94246
4.46143
4.44929
4.44150
4.43770
4.43007
4.42323
4.41968
4.39798
4.38600
4.37337
4.36036
3.95229
3.94586
3.93349
3.92298
3.91059
3.89652
3.88390
1.27129
0.09113
2.0000
1.0772
2.0089
1.1438
1.9515
2.0934
Integral
ppm
Br
O
Br

11Cb

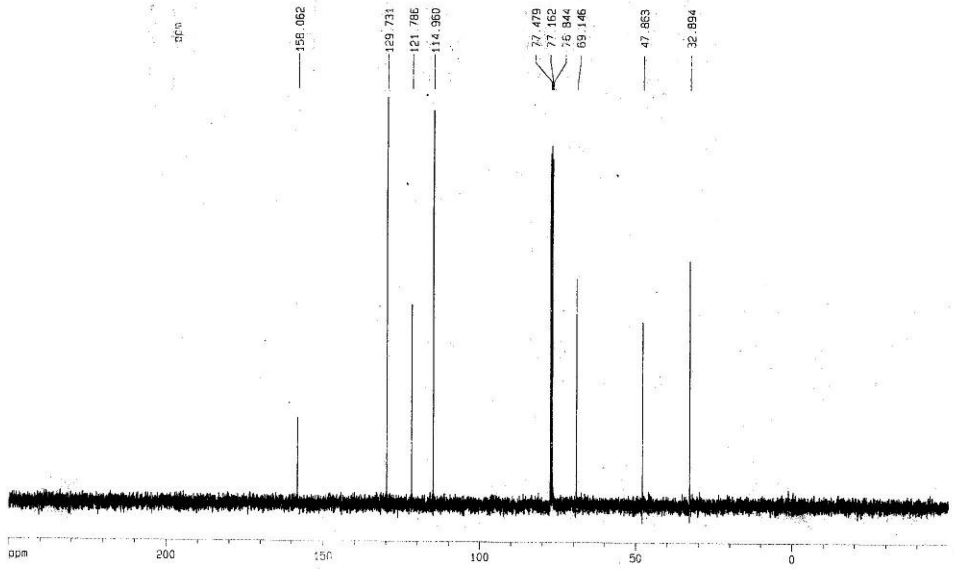
158.062
129.731
121.786
114.960
77.479
77.162
76.844
69.146
47.863
32.894
ppm
200
150
100
50
0

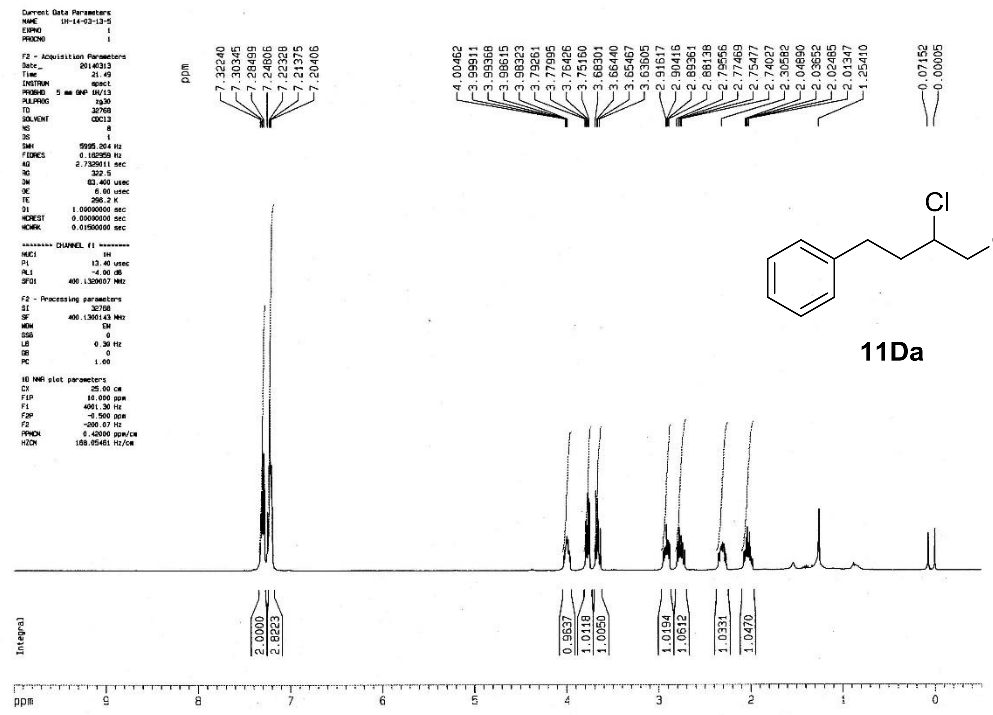

7.32240
7.30345
7.28499
7.24806
7.22328
7.21375
7.20406
4.00462
3.99911
3.99368
3.98615
3.98323
3.79261
3.77995
3.76426
3.75160
3.68301
3.66440
3.65467
3.63605
2.91617
2.90416
2.89361
2.88138
2.79556
2.77469
2.75477
2.74027
2.30582
2.04890
2.03652
2.02485
2.01347
1.25410
0.07152
0.00005
Cl
Cl
2.0000
2.8223
0.9837
1.0118
1.0050
1.0194
1.0612
1.0331
1.0470
Integral
ppm

11Da

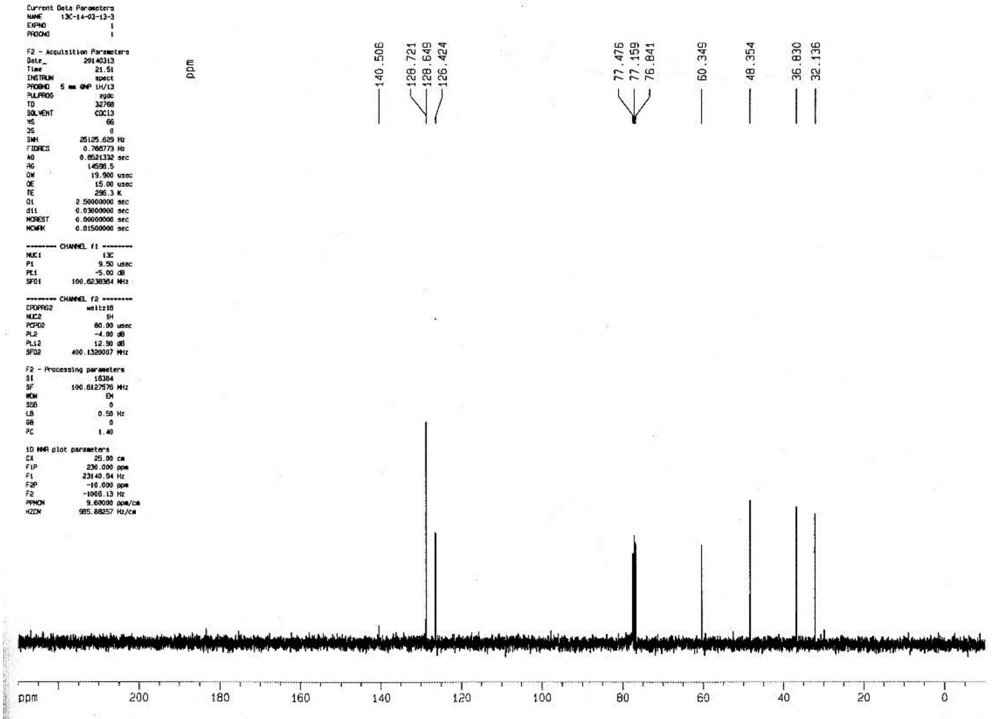

140.506
128.721
128.649
126.424
77.476
77.159
76.841
60.349
48.354
36.830
32.136
ppm
200
180
160
140
120
100
80
60
40
20
0

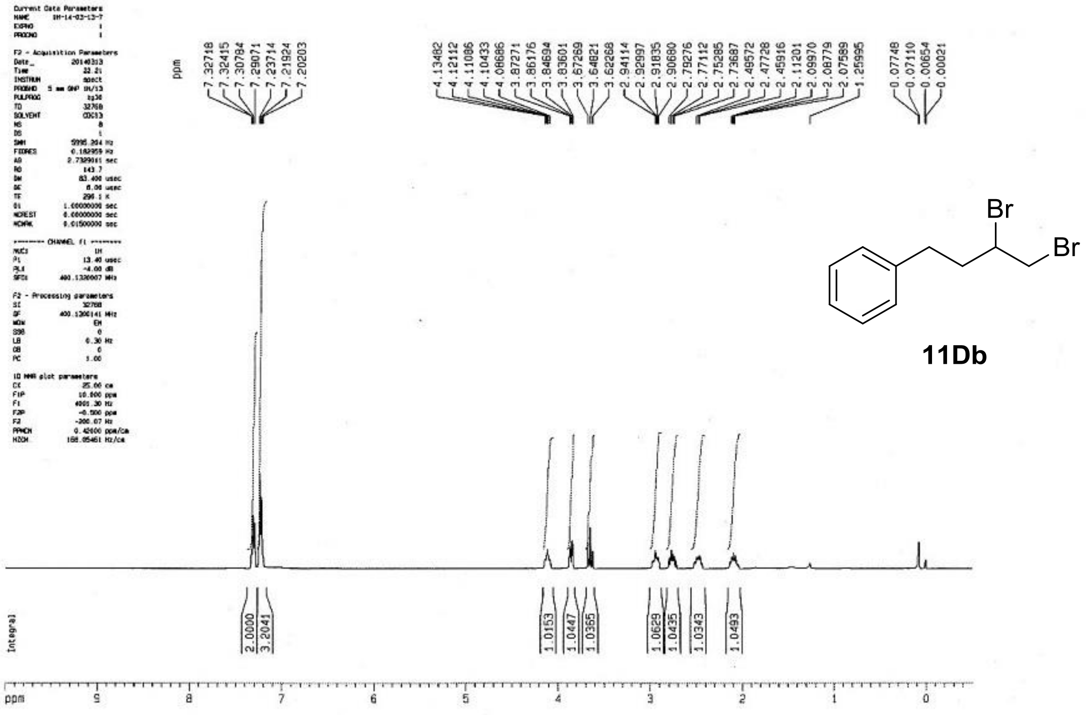
ppm
7.32718
7.32415
7.30784
7.29071
7.23714
7.21924
7.20203
4.13482
4.12112
4.11086
4.10433
4.08686
3.87271
3.86176
3.84694
3.83601
3.67269
3.64821
3.62268
2.94114
2.92997
2.91835
2.90680
2.79276
2.77112
2.75285
2.73687
2.49572
2.47728
2.45916
2.11201
2.09970
2.08779
2.07589
1.25995
0.07748
0.07110
0.00654
0.00021
Integral
2.0000
3.2041
1.0153
1.0447
1.0365
1.0629
1.0435
1.0343
1.0493
Br
Br

11Db

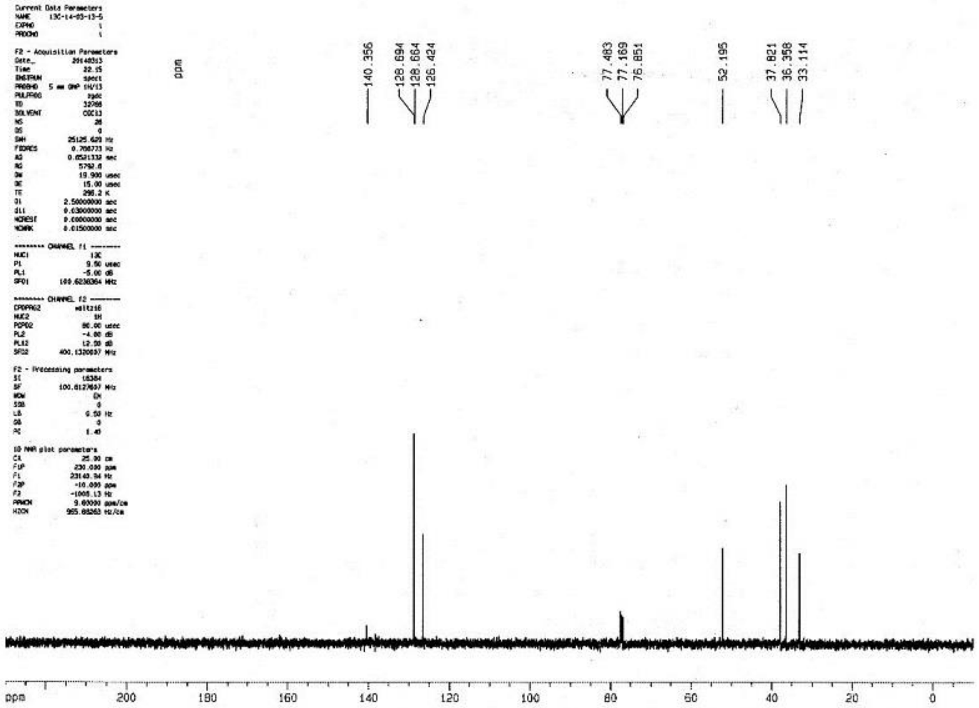
ppm
140.356
128.694
128.664
126.424
77.483
77.169
76.851
52.195
37.821
36.358
33.114

X-ray Structure Details

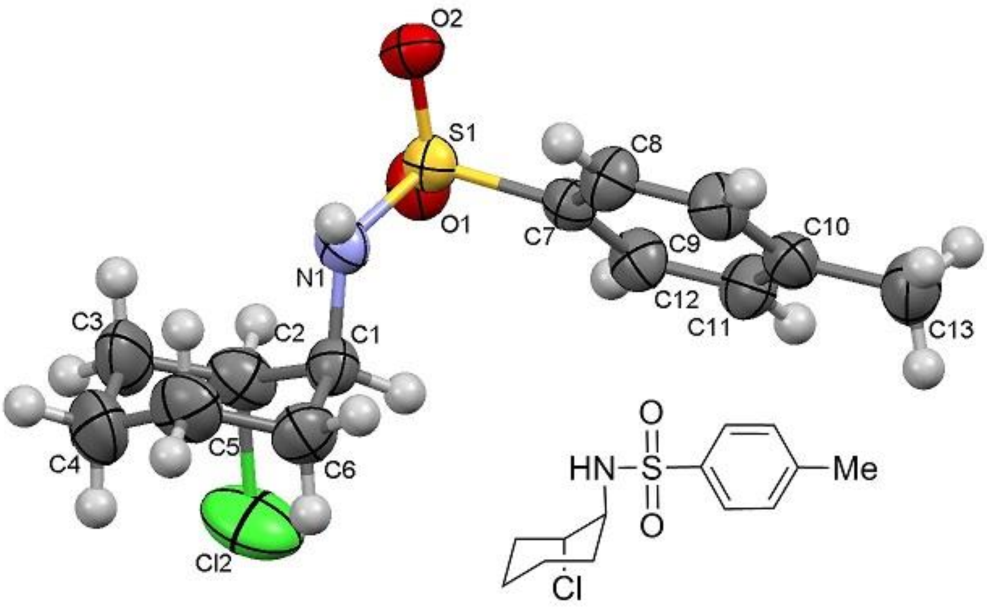

X-ray structure of *N*-(2-chlorocyclohexyl)-4-methylbenzenesulfonamide

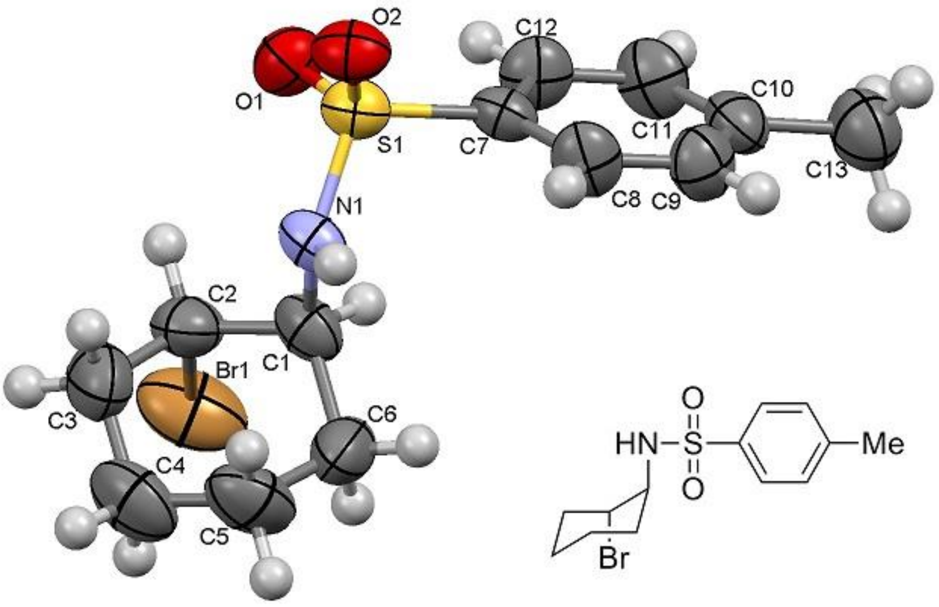

X-ray structure of *N*-(2-bromocyclohexyl)-4-methylbenzenesulfonamide

Refinement

The structures were solved by direct methods, *SHELXS-97* (Sheldrick, 2008). All non-H atoms were refined anisotropically.

All of the C-bound H atoms were observable from difference Fourier map but were all placed at geometrical positions with C—H = 0.93, 0.96, 0.97 and 0.98Å for phenyl, methyl, methylene and methine H-atoms. The N-bound H atom was located from difference Fourier map. All H-atoms were refined using riding model with U_{iso}(H) = 1.2U_{eq}(Carrier).

Highest peak is 0.44 at (0.5145, 0.9409, 0.6173) [0.96Å from Cl1] Deepest hole is -0.48 at (0.2792, 0.0893, 0.3290) [0.70Å from Cl1]

Computing details

Data collection: *APEX* (Bruker AXS Inc, 2007); cell refinement: *SAINT* v7.34A (Bruker AXS Inc, 2007); data reduction: *CrystalStructure* (Rigaku/MSC and Rigaku Corporation, 2006); program(s) used to solve structure: *SHELXS97* (Sheldrick, 2008); program(s) used to refine structure: *SHELXL97* (Sheldrick, 2008); molecular graphics: Mercury (Macrae *et al.*, 2008); software used to prepare material for publication: *SHELXL97* (Sheldrick, 2008).

References

Bruker AXS Inc. (2007). *ApexII*, Madison, Wisconsin, USA.

Macrae, C. F., Bruno, I. J., Chisholm, J. A., Edgington, P. R., McCabe, P., Pidcock, E., Rodriguez-Monge, L., Taylor, R., van de Streek, J. & Wood, P. A. (2008). *Mercury J. Appl. Cryst*, **41**, 466–470.

Rigaku/MSC and Rigaku Corporation. (2006). *CrystalStructure*. Single Crystal Structure Analysis Software. Version 3.8.1. Rigaku/MSC, 9009 New Trails Drive, The Woodlands, TX, USA 77381-5209. Rigaku, 3-9-12 Akishima, Tokyo 196-8666, Japan.

Sheldrick, G. M. (2008). *SADABS*, Göttingen University, Göttingen, Germany.

Sheldrick, G. M. (2008). *SHELX* programs. (*SHELXL97, SHELXS97*.) *Acta Cryst.* **E68**, 112-122.

X-ray report of *N*-(2-chlorocyclohexyl)-4-methylbenzenesulfonamide

The compound *N*-(2-chlorocyclohexyl)-4-methylbenzenesulfonamide crystallized in a centrosymmetric triclinic primitive space group, *P* -1 (#2).There are two complex molecules in the unit cell. The cyclo-hexyl ring is in the chair form with the puckering amplitude (Q) of 0.535(4)Å, θ value of 176.3(4)° and ϕ value of 35(6)°. Both the chloride and the sulphonamide groups are at axial positions. The compound is racemic. The atoms C1 and C2 are of the same configuration.

All the bond parameters are comparable to its bromide analogue. C—Cl is 1.818(3)Å, S=O is 1.4268(18)–1.4365(18)Å; S–N is 1.616(2)Å.

There are only very weak π···π inter-actions between the neighbouring tolyl rings. The complementary pair of inter-molecular N1—H1N···O2 H-bonding inter-actions link the molecules into dimers. There is no residual solvent accessible void volume found in the unit cell.

Experimental

A colourless block crystal of, $C_{13}H_{18}ClNO_2S$, having approximate dimensions of 0.38 mm *x* 0.56 mm *x* 0.56 mm was mounted in glass capillary. All measurements were made on a Bruker *Apex* CCD detector with graphite monochromated Mo—Kα radiation.

Cell constants and an orientation matrix for data collection corresponded to a primitive monoclinic cell with dimensions: a = 6.9260(2)Å, b = 9.4804(3)Å, c = 11.6065(4)Å, V = 714.23(4)$Å^3$ α = 73.524(2)°, β = 77.837(2)° γ = 87.759(2)°.

For *Z* = 2 and F.W. = 287.79, the calculated density is 1.338 g/cm^3. Based on a statistical analysis of intensity distribution, and the successful solution and refinement of the structure, the space group was determined to be: *P* -1 (#2)

The data were collected at a temperature of 23(1)°C to a maximum 2θ value of 25.02°.

Of the 11110 reflections that were collected, 2480 reflections were unique. (R_{int} = 0.0275); equivalent reflections were merged.

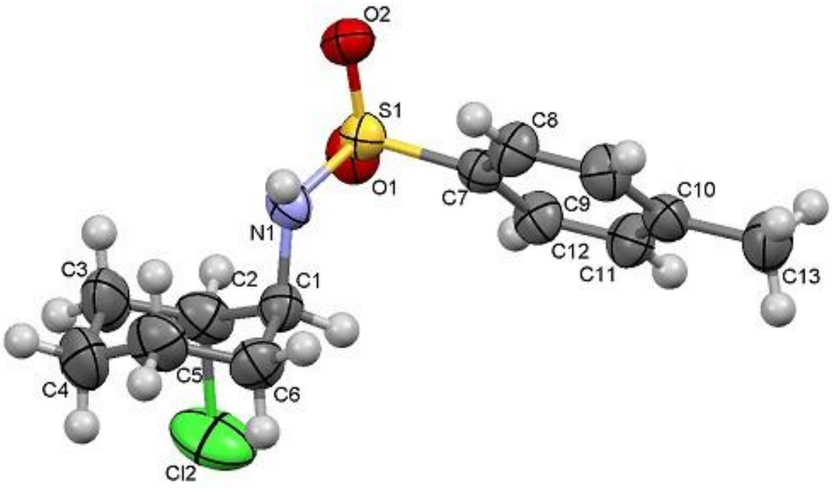

Figure 1 The compound shown at 50% probability thermal ellipsoids with the atom numbering scheme.

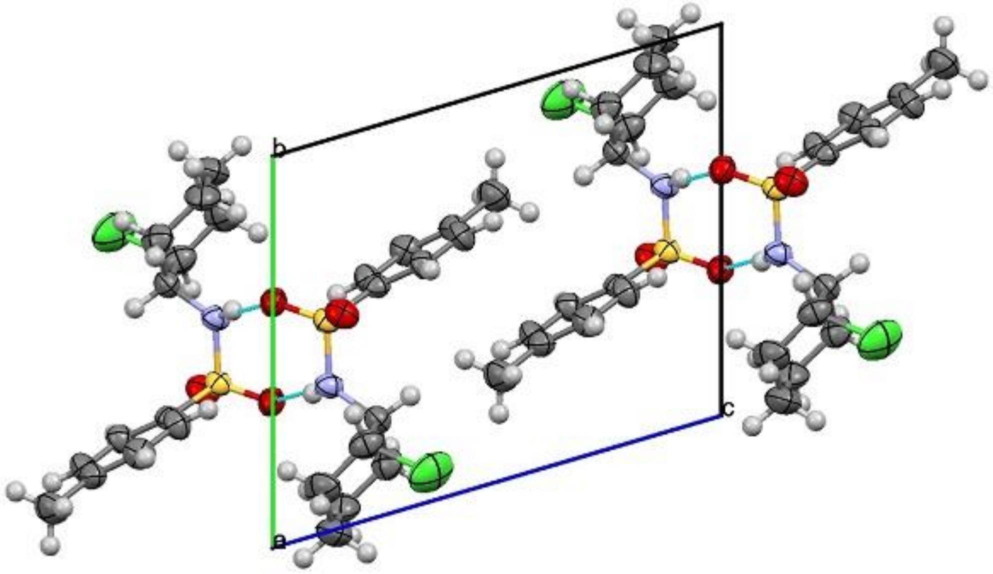

Figure 2 The packing diagram of the unit cell was projected along the *a* axis, shown at 50% probability thermal ellipsoids. The cyan dotted lines represent the complementary pair of intermolecular N1-H1N···O2 H-bonding interactions linking the molecules into dimers.

Crystal data

$C_{13}H_{18}ClNO_2S$	Z = 2
M_r = 287.79	F(000) = 304
Triclinic, $P\bar{1}$	D_x = 1.338 Mg m^{-3}
Hall symbol: -P 1	Mo $K\alpha$ radiation, λ = 0.71073 Å
a = 6.9260(2)Å	Cell parameters from 11110 reflections
b = 9.4804(3)Å	θ = 1.9–25.0°
c = 11.6065(4)Å	μ = 0.41 mm^{-1}
α = 73.524(2)°	T = 296 K
β = 77.837(2)°	Block, colourless
γ = 87.759(2)°	0.56 × 0.56 × 0.38 mm
V = 714.23(4)Å^3	

Data collection

Bruker APEX CCD diffractometer	2480 independent reflections
Radiation source: fine-focus sealed tube	2119 reflections with $I > 2\sigma(I)$
graphite	R_{int} = 0.028
ω & ϕ scans	θ_{max} = 25.0°, θ_{min} = 1.9°
Absorption correction: multi-scan *SADABS* (Sheldrick, 2008)	h = -8→8
T_{min} = 0.804, T_{max} = 0.861	k = -11→11
11110 measured reflections	l = -13→13

Refinement

Refinement on F^2	Primary atom site location: structure-invariant direct methods
Least-squares matrix: full	Secondary atom site location: difference Fourier map
$R[F^2 > 2\sigma(F^2)]$ = 0.045	Hydrogen site location: inferred from neighbouring sites
$wR(F^2)$ = 0.127	H-atom parameters constrained
S = 1.06	$w = 1/[\sigma^2(F_o^2) + (0.0607P)^2 + 0.4124P]$ where $P = (F_o^2 + 2F_c^2)/3$
2480 reflections	$(\Delta/\sigma)_{max}$ = 0.006
164 parameters	$\Delta\rho_{max}$ = 0.44 e Å^{-3}
0 restraints	$\Delta\rho_{min}$ = -0.48 e Å^{-3}

Special details

Geometry. All esds (except the esd in the dihedral angle between two l.s. planes) are estimated using the full covariance matrix. The cell esds are taken into account individually in the estimation of esds in distances, angles and torsion angles; correlations between esds in cell parameters are only used when they are defined by crystal symmetry. An approximate (isotropic) treatment of cell esds is used for estimating esds involving l.s. planes.

Refinement. Refinement of F^2 against ALL reflections. The weighted R-factor wR and goodness of fit S are based on F^2, conventional R-factors R are based on F, with F set to zero for negative F^2. The threshold expression of $F^2 > 2sigma(F^2)$ is used only for calculating R-factors(gt) etc. and is not relevant to the choice of reflections for refinement. R-factors based on F^2 are statistically about twice as large as those based on F, and R-factors based on ALL data will be even larger.

Fractional atomic coordinates and isotropic or equivalent isotropic displacement parameters (Å^2)

	x	*y*	*z*	U_{iso}*/U_{eq}
Cl1	0.63511 (16)	0.92618 (12)	0.64754 (11)	0.1109 (4)
S1	0.28780 (9)	0.46056 (6)	0.87910 (6)	0.0428 (2)
O1	0.4977 (3)	0.4550 (2)	0.84511 (18)	0.0566 (5)
O2	0.1860 (3)	0.37354 (19)	0.99763 (16)	0.0521 (5)
N1	0.2296 (3)	0.6289 (2)	0.87325 (19)	0.0461 (5)
H1N	0.0941	0.6362	0.9106	0.055*
C1	0.3156 (4)	0.7518 (3)	0.7656 (2)	0.0487 (6)
H1	0.3512	0.7157	0.6929	0.058*
C2	0.5015 (4)	0.8056 (3)	0.7917 (3)	0.0621 (7)
H2	0.5832	0.7209	0.8187	0.075*
C3	0.4571 (6)	0.8854 (4)	0.8886 (3)	0.0798 (10)
H3A	0.5790	0.9275	0.8943	0.096*
H3B	0.4052	0.8154	0.9674	0.096*
C4	0.3092 (6)	1.0073 (4)	0.8630 (4)	0.0861 (11)
H4A	0.2784	1.0499	0.9313	0.103*
H4B	0.3672	1.0843	0.7899	0.103*

C5	0.1231 (5)	0.9493 (3)	0.8448 (3)	0.0741 (9)
H5A	0.0345	1.0299	0.8238	0.089*
H5B	0.0578	0.8802	0.9209	0.089*
C6	0.1654 (4)	0.8733 (3)	0.7437 (3)	0.0614 (7)
H6A	0.2151	0.9456	0.6659	0.074*
H6B	0.0432	0.8314	0.7383	0.074*
C7	0.1883 (3)	0.4103 (2)	0.7678 (2)	0.0409 (5)
C8	-0.0132 (4)	0.3866 (3)	0.7871 (2)	0.0513 (6)
H8	-0.0967	0.4013	0.8565	0.062*
C9	-0.0891 (4)	0.3411 (3)	0.7029 (3)	0.0563 (7)
H9	-0.2246	0.3248	0.7166	0.068*
C10	0.0314 (4)	0.3191 (3)	0.5979 (2)	0.0528 (7)
C11	0.2314 (4)	0.3457 (3)	0.5796 (3)	0.0591 (7)
H11	0.3144	0.3327	0.5093	0.071*
C12	0.3122 (4)	0.3913 (3)	0.6627 (2)	0.0512 (6)
H12	0.4475	0.4089	0.6484	0.061*
C13	-0.0555 (5)	0.2675 (4)	0.5079 (3)	0.0749 (9)
H13A	-0.0202	0.3364	0.4280	0.090*
H13B	-0.1967	0.2605	0.5339	0.090*
H13C	-0.0048	0.1727	0.5047	0.090*

Atomic displacement parameters (Å^2)

	U^{11}	U^{22}	U^{33}	U^{12}	U^{13}	U^{23}
Cl1	0.0846 (7)	0.0878 (7)	0.1249 (9)	-0.0193 (5)	0.0215 (6)	-0.0025 (6)
S1	0.0387 (4)	0.0433 (3)	0.0469 (4)	0.0074 (2)	-0.0075 (3)	-0.0155 (3)
O1	0.0388 (10)	0.0639 (12)	0.0721 (13)	0.0100 (8)	-0.0116 (9)	-0.0283 (10)
O2	0.0547 (11)	0.0511 (10)	0.0458 (10)	0.0072 (8)	-0.0090 (8)	-0.0082 (8)
N1	0.0435 (12)	0.0427 (11)	0.0515 (12)	0.0032 (9)	-0.0023 (9)	-0.0182 (9)
C1	0.0539 (15)	0.0502 (14)	0.0452 (14)	0.0029 (12)	-0.0098 (12)	-0.0192 (11)
C2	0.0540 (17)	0.0529 (16)	0.077 (2)	0.0019 (13)	-0.0178 (14)	-0.0117 (14)
C3	0.101 (3)	0.067 (2)	0.089 (2)	-0.0015 (18)	-0.051 (2)	-0.0276 (18)
C4	0.128 (3)	0.0575 (18)	0.086 (2)	0.009 (2)	-0.032 (2)	-0.0351 (18)

C5	0.086 (2)	0.0538 (17)	0.081 (2)	0.0253 (16)	-0.0157 (18)	-0.0216 (16)
C6	0.0635 (18)	0.0592 (17)	0.0641 (18)	0.0080 (14)	-0.0281 (14)	-0.0116 (14)
C7	0.0411 (13)	0.0354 (12)	0.0458 (13)	0.0062 (10)	-0.0072 (10)	-0.0129 (10)
C8	0.0422 (14)	0.0623 (16)	0.0510 (15)	0.0070 (12)	-0.0053 (11)	-0.0222 (13)
C9	0.0444 (15)	0.0625 (17)	0.0648 (17)	0.0019 (12)	-0.0129 (13)	-0.0211 (14)
C10	0.0658 (18)	0.0428 (13)	0.0532 (15)	0.0041 (12)	-0.0183 (13)	-0.0148 (12)
C11	0.0670 (19)	0.0608 (17)	0.0505 (16)	0.0057 (14)	-0.0015 (13)	-0.0254 (13)
C12	0.0441 (15)	0.0547 (15)	0.0543 (16)	0.0035 (12)	-0.0015 (12)	-0.0209 (12)
C13	0.094 (2)	0.070 (2)	0.074 (2)	0.0020 (18)	-0.0320 (19)	-0.0303 (17)

Geometric parameters (Å, º)

Cl1—C2	1.818 (3)	C5—H5A	0.9700
S1—O1	1.4268 (18)	C5—H5B	0.9700
S1—O2	1.4365 (18)	C6—H6A	0.9700
S1—N1	1.616 (2)	C6—H6B	0.9700
S1—C7	1.767 (2)	C7—C8	1.383 (3)
N1—C1	1.480 (3)	C7—C12	1.388 (3)
N1—H1N	0.9561	C8—C9	1.374 (4)
C1—C2	1.520 (4)	C8—H8	0.9300
C1—C6	1.525 (4)	C9—C10	1.389 (4)
C1—H1	0.9800	C9—H9	0.9300
C2—C3	1.501 (5)	C10—C11	1.379 (4)
C2—H2	0.9800	C10—C13	1.506 (4)
C3—C4	1.521 (5)	C11—C12	1.383 (4)
C3—H3A	0.9700	C11—H11	0.9300
C3—H3B	0.9700	C12—H12	0.9300
C4—C5	1.499 (5)	C13—H13A	0.9600
C4—H4A	0.9700	C13—H13B	0.9600
C4—H4B	0.9700	C13—H13C	0.9600
C5—C6	1.516 (4)		
O1—S1—O2	119.78 (11)	C6—C5—H5A	109.4

O1—S1—N1	108.11 (11)	C4—C5—H5B	109.4
O2—S1—N1	105.61 (11)	C6—C5—H5B	109.4
O1—S1—C7	107.50 (11)	H5A—C5—H5B	108.0
O2—S1—C7	107.29 (11)	C5—C6—C1	112.5 (2)
N1—S1—C7	108.09 (11)	C5—C6—H6A	109.1
C1—N1—S1	121.06 (16)	C1—C6—H6A	109.1
C1—N1—H1N	116.9	C5—C6—H6B	109.1
S1—N1—H1N	111.7	C1—C6—H6B	109.1
N1—C1—C2	107.4 (2)	H6A—C6—H6B	107.8
N1—C1—C6	110.0 (2)	C8—C7—C12	120.1 (2)
C2—C1—C6	111.9 (2)	C8—C7—S1	119.70 (19)
N1—C1—H1	109.2	C12—C7—S1	120.18 (19)
C2—C1—H1	109.2	C9—C8—C7	119.5 (2)
C6—C1—H1	109.2	C9—C8—H8	120.2
C3—C2—C1	112.6 (3)	C7—C8—H8	120.2
C3—C2—Cl1	110.5 (2)	C8—C9—C10	121.7 (3)
C1—C2—Cl1	107.0 (2)	C8—C9—H9	119.2
C3—C2—H2	108.9	C10—C9—H9	119.2
C1—C2—H2	108.9	C11—C10—C9	117.8 (2)
Cl1—C2—H2	108.9	C11—C10—C13	121.7 (3)
C2—C3—C4	113.0 (3)	C9—C10—C13	120.6 (3)
C2—C3—H3A	109.0	C10—C11—C12	121.9 (2)
C4—C3—H3A	109.0	C10—C11—H11	119.1
C2—C3—H3B	109.0	C12—C11—H11	119.1
C4—C3—H3B	109.0	C11—C12—C7	119.0 (2)
H3A—C3—H3B	107.8	C11—C12—H12	120.5
C5—C4—C3	110.9 (3)	C7—C12—H12	120.5
C5—C4—H4A	109.5	C10—C13—H13A	109.5
C3—C4—H4A	109.5	C10—C13—H13B	109.5
C5—C4—H4B	109.5	H13A—C13—H13B	109.5
C3—C4—H4B	109.5	C10—C13—H13C	109.5
H4A—C4—H4B	108.0	H13A—C13—H13C	109.5

C4—C5—C6	111.4 (3)	H13B—C13—H13C	109.5
C4—C5—H5A	109.4		

Hydrogen-bond geometry (Å, º)

D—H⋯*A*	*D*—H	H⋯*A*	*D*⋯*A*	*D*—H⋯*A*
N1—H1*N*⋯O2^{i}	0.96	2.00	2.949 (3)	172.9
C2—H2⋯O1	0.98	2.53	3.204 (3)	126
C12—H12⋯O1	0.93	2.54	2.903 (3)	104

Symmetry code: (i) -*x*, -*y*+1, -*z*+2.

X-ray report of *N*-(2-bromocyclohexyl)-4-methylbenzenesulfonamide

The compound *N*-(2-bromocyclohexyl)-4-methylbenzenesulfonamide crystallized in a centrosymmetric monoclinic primitive space group, *P* 2_1/c (# 14). There are a total of four complex molecules in the unit cell. The cyclo-hexyl ring is in the chair form with the puckering amplitude (Q) of 0.529(9)Å, θ value of 176.1(10)° and ϕ value of 59(12)°. Both the bromide and the sulphonamide groups are at axial positions. The compound is racemic. The atoms C1 and C2 are of the same configuration.

All the bond parameters are comparable within normal ranges. C—Br is 1.985(7)Å, S=O is 1.429(5)–1.441(4)Å; S–N is 1.615(5)Å.

There are weak π⋯π inter-actions between the neighbouring tolyl rings. Inter-molecular H-bonding inter-actions present. There is no residual solvent accessible void volume found in the unit cell.

Experimental

A colourless plate crystal of, $C_{13}H_{18}BrNO_2S$, having approximate dimensions of 0.14 mm *x* 0.26 mm *x* 0.38 mm was mounted in glass capillary. All measurements were made on a Bruker *Apex* CCD detector with graphite monochromated Mo—Kα radiation.

Cell constants and an orientation matrix for data collection corresponded to a primitive monoclinic cell with dimensions: a = 10.8559(4)Å, b = 13.6017(5)Å, c = 10.5811(4)Å, V = 1482.46(10)Å^3 β = 108.406(2)°.

For $Z = 4$ and F.W. = 332.25, the calculated density is 1.489 g/cm^3. Based on a statistical analysis of intensity distribution, and the successful solution and refinement of the structure, the space group was determined to be: $P\ 2_1/c$ (#14)

The data were collected at a temperature of 23(1)°C to a maximum 2θ value of 66.1°.

Of the 17462 reflections that were collected, 2616 reflections were unique. (R_{int} = 0.0738); equivalent reflections were merged.

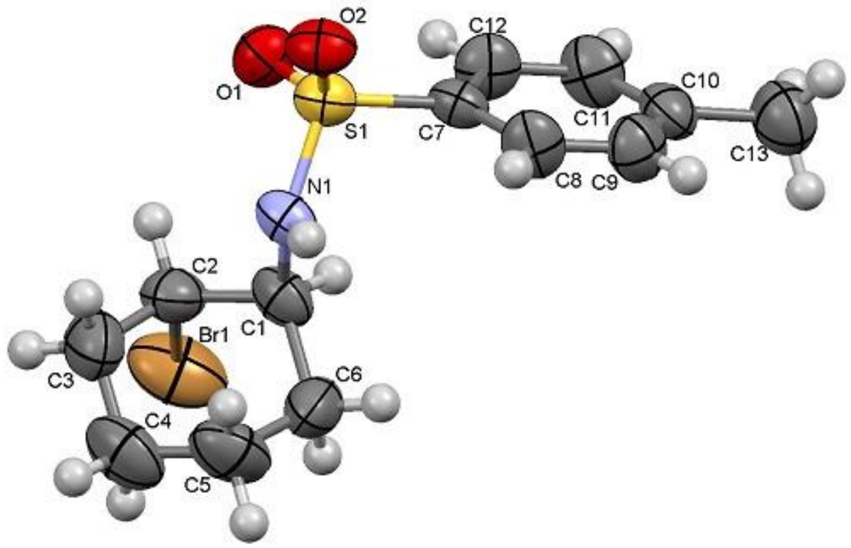

Figure 3 The *ORTEP* plot of the compound was shown at 50% probability thermal ellipsoids with the atom numbering scheme.

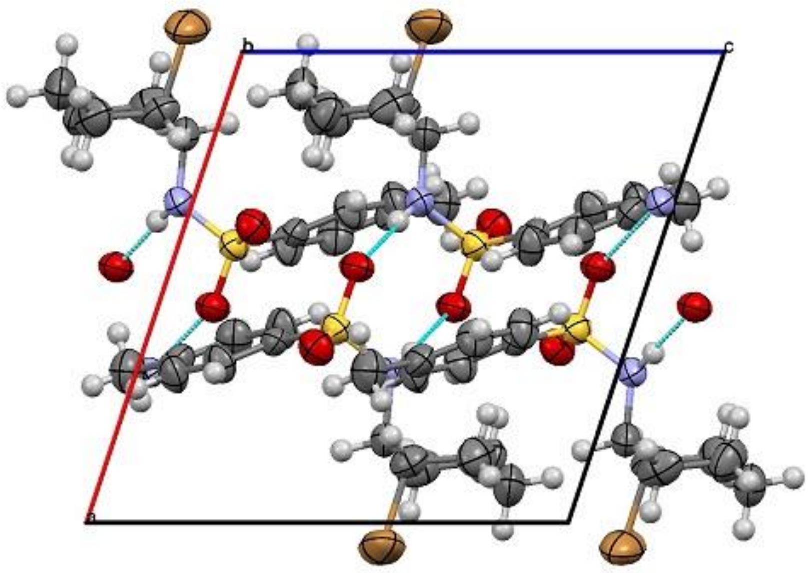

Figure 4 The packing diagram of the unit cell was shown at 50% probability thermal ellipsoids. The cyan dotted line represent the complementary pair of intermolecular N1-H1N···O2 H-bonding interactions linking the molecules into dimers.

Crystal data

$C_{13}H_{18}BrNO_2S$	*F*(000) = 680
M_r = 332.25	D_x = 1.489 Mg m^{-3}
Monoclinic, $P2_1/c$	Mo *K*α radiation, l = 0.71073 Å
Hall symbol: -P 2ybc	Cell parameters from 17462 reflections
a = 10.8559(4)Å	θ = 1.5–25.0°
b = 13.6017(5)Å	μ = 2.91 mm^{-1}
c = 10.5811(4)Å	*T* = 296 K
β = 108.406(2)°	Plate, colourless
V = 1482.46(10)$Å^3$	0.38 × 0.26 × 0.14 mm
Z = 4	

Data collection

Bruker APEX CCD diffractometer	2616 independent reflections
Radiation source: fine-focus sealed tube	1601 reflections with $I > 2\sigma(I)$
graphite	R_{int} = 0.074
ω & ϕ scans	θ_{max} = 25.0°, q_{min} = 2.0°
Absorption correction: multi-scan *SADABS* (Sheldrick, 2008)	$h = -11 \rightarrow 12$
T_{min} = 0.405, T_{max} = 0.686	$k = -16 \rightarrow 16$
17462 measured reflections	$l = -12 \rightarrow 12$

Refinement

Refinement on F^2	Primary atom site location: structure-invariant direct methods
Least-squares matrix: full	Secondary atom site location: difference Fourier map
$R[F^2 > 2\sigma(F^2)]$ = 0.068	Hydrogen site location: inferred from neighbouring sites
$wR(F^2)$ = 0.201	H-atom parameters constrained
S = 1.04	$w = 1/[\sigma^2(F_o^2) + (0.0872P)^2 + 3.5474P]$ where $P = (F_o^2 + 2F_c^2)/3$
2616 reflections	$(\Delta/\sigma)_{max}$ = 0.019
164 parameters	$\Delta\rho_{max}$ = 1.55 e Å^{-3}
0 restraints	$\Delta\rho_{min}$ = -0.81 e Å^{-3}

Special details

Geometry. All esds (except the esd in the dihedral angle between two l.s. planes) are estimated using the full covariance matrix. The cell esds are taken into account individually in the estimation of esds in distances, angles and torsion angles; correlations between esds in cell parameters are only used when they are defined by crystal symmetry. An approximate (isotropic) treatment of cell esds is used for estimating esds involving l.s. planes.

Refinement. Refinement of F^2 against ALL reflections. The weighted R-factor wR and goodness of fit S are based on F^2, conventional R-factors R are based on F, with F set to zero for negative F^2. The threshold expression of F^2 > 2sigma(F^2) is used only for calculating R-factors(gt) etc. and is not relevant to the choice of reflections for refinement. R-factors based on F^2 are statistically about twice as large as those based on F, and R-factors based on ALL data will be even larger.

Fractional atomic coordinates and isotropic or equivalent isotropic displacement parameters (Å^2)

	x	*y*	*z*	U_{iso}*/U_{eq}
Br1	1.05259 (9)	0.31013 (9)	0.63054 (10)	0.1096 (5)
S1	0.58845 (16)	0.39459 (12)	0.38391 (16)	0.0520 (5)
O1	0.6280 (5)	0.2988 (3)	0.3570 (5)	0.0646 (12)
O2	0.4575 (4)	0.4086 (3)	0.3858 (4)	0.0632 (12)
N1	0.6808 (5)	0.4284 (4)	0.5293 (5)	0.0537 (13)
H1N	0.6400	0.4783	0.5605	0.064*
C1	0.8245 (7)	0.4251 (5)	0.5627 (6)	0.0612 (18)
H1	0.8472	0.4297	0.4803	0.073*
C2	0.8705 (7)	0.3288 (5)	0.6279 (7)	0.0683 (19)
H2	0.8172	0.2766	0.5735	0.082*
C3	0.8607 (9)	0.3220 (6)	0.7644 (8)	0.084 (2)
H3A	0.9024	0.2618	0.8056	0.101*
H3B	0.7698	0.3180	0.7584	0.101*
C4	0.9212 (9)	0.4070 (8)	0.8520 (7)	0.094 (3)
H4A	0.9050	0.4007	0.9367	0.113*
H4B	1.0144	0.4059	0.8691	0.113*
C5	0.8689 (9)	0.5011 (7)	0.7901 (8)	0.091 (3)
H5A	0.9133	0.5546	0.8471	0.109*
H5B	0.7774	0.5051	0.7816	0.109*
C6	0.8855 (8)	0.5124 (6)	0.6535 (8)	0.081 (2)
H6A	0.9772	0.5163	0.6630	0.097*
H6B	0.8447	0.5730	0.6129	0.097*
C7	0.6156 (6)	0.4763 (5)	0.2655 (5)	0.0485 (15)
C8	0.5881 (7)	0.5746 (5)	0.2724 (7)	0.0647 (19)
H8	0.5562	0.5977	0.3387	0.078*
C9	0.6087 (7)	0.6380 (5)	0.1799 (7)	0.0677 (19)
H9	0.5903	0.7044	0.1843	0.081*
C10	0.6559 (7)	0.6054 (6)	0.0807 (6)	0.0620 (18)
C11	0.6824 (7)	0.5081 (6)	0.0772 (7)	0.071 (2)
H11	0.7148	0.4853	0.0111	0.085*

C12	0.6632 (7)	0.4419 (5)	0.1674 (7)	0.0630 (18)
H12	0.6819	0.3755	0.1624	0.076*
C13	0.6785 (9)	0.6785 (6)	-0.0179 (8)	0.089 (3)
H13A	0.7094	0.6444	-0.0814	0.106*
H13B	0.7419	0.7260	0.0288	0.106*
H13C	0.5985	0.7113	-0.0635	0.106*

Atomic displacement parameters (Å^2)

	U^{11}	U^{22}	U^{33}	U^{12}	U^{13}	U^{23}
Br1	0.0643 (6)	0.1545 (11)	0.1021 (8)	0.0326 (6)	0.0149 (5)	-0.0239 (6)
S1	0.0471 (9)	0.0586 (10)	0.0518 (9)	0.0023 (7)	0.0177 (7)	-0.0078 (8)
O1	0.069 (3)	0.057 (3)	0.071 (3)	0.001 (2)	0.028 (2)	-0.007 (2)
O2	0.046 (3)	0.079 (3)	0.065 (3)	-0.002 (2)	0.019 (2)	-0.013 (2)
N1	0.046 (3)	0.074 (4)	0.043 (3)	0.008 (3)	0.015 (2)	-0.002 (2)
C1	0.061 (4)	0.081 (5)	0.043 (4)	0.006 (4)	0.018 (3)	0.003 (3)
C2	0.058 (4)	0.072 (5)	0.065 (5)	-0.007 (4)	0.007 (3)	-0.006 (4)
C3	0.083 (6)	0.089 (6)	0.080 (6)	-0.011 (5)	0.024 (4)	0.021 (5)
C4	0.095 (7)	0.136 (9)	0.052 (5)	0.010 (6)	0.025 (4)	0.000 (5)
C5	0.099 (7)	0.101 (7)	0.063 (5)	0.008 (5)	0.010 (5)	-0.023 (5)
C6	0.073 (5)	0.063 (5)	0.091 (6)	-0.013 (4)	0.005 (4)	0.015 (4)
C7	0.042 (3)	0.062 (4)	0.039 (3)	0.002 (3)	0.010 (3)	-0.006 (3)
C8	0.085 (5)	0.059 (5)	0.058 (4)	0.017 (4)	0.033 (4)	-0.008 (3)
C9	0.079 (5)	0.056 (4)	0.069 (5)	0.014 (4)	0.025 (4)	0.003 (4)
C10	0.055 (4)	0.076 (5)	0.048 (4)	0.002 (4)	0.007 (3)	0.008 (4)
C11	0.081 (5)	0.084 (6)	0.060 (4)	0.010 (4)	0.040 (4)	-0.001 (4)
C12	0.073 (5)	0.058 (4)	0.067 (4)	0.012 (3)	0.034 (4)	-0.010 (4)
C13	0.099 (7)	0.097 (6)	0.071 (5)	0.001 (5)	0.029 (5)	0.013 (5)

Geometric parameters (Å, º)

Br1—C2	1.985 (7)	C5—H5A	0.9700

S1—O1	1.429 (5)	C5—H5B	0.9700
S1—O2	1.441 (4)	C6—H6A	0.9700
S1—N1	1.615 (5)	C6—H6B	0.9700
S1—C7	1.768 (6)	C7—C8	1.377 (9)
N1—C1	1.488 (8)	C7—C12	1.380 (8)
N1—H1N	0.9264	C8—C9	1.375 (9)
C1—C2	1.491 (10)	C8—H8	0.9300
C1—C6	1.539 (10)	C9—C10	1.378 (10)
C1—H1	0.9800	C9—H9	0.9300
C2—C3	1.485 (11)	C10—C11	1.357 (10)
C2—H2	0.9800	C10—C13	1.517 (10)
C3—C4	1.497 (12)	C11—C12	1.375 (10)
C3—H3A	0.9700	C11—H11	0.9300
C3—H3B	0.9700	C12—H12	0.9300
C4—C5	1.468 (12)	C13—H13A	0.9600
C4—H4A	0.9700	C13—H13B	0.9600
C4—H4B	0.9700	C13—H13C	0.9600
C5—C6	1.520 (11)		
O1—S1—O2	118.9 (3)	C6—C5—H5A	109.3
O1—S1—N1	108.3 (3)	C4—C5—H5B	109.3
O2—S1—N1	105.5 (3)	C6—C5—H5B	109.3
O1—S1—C7	107.7 (3)	H5A—C5—H5B	108.0
O2—S1—C7	108.2 (3)	C5—C6—C1	111.1 (6)
N1—S1—C7	107.7 (3)	C5—C6—H6A	109.4
C1—N1—S1	120.4 (4)	C1—C6—H6A	109.4
C1—N1—H1N	121.4	C5—C6—H6B	109.4
S1—N1—H1N	109.0	C1—C6—H6B	109.4
N1—C1—C2	108.0 (6)	H6A—C6—H6B	108.0
N1—C1—C6	109.7 (6)	C8—C7—C12	120.6 (6)
C2—C1—C6	111.9 (6)	C8—C7—S1	119.0 (5)
N1—C1—H1	109.1	C12—C7—S1	120.4 (5)

C2—C1—H1	109.1	C9—C8—C7	119.0 (6)
C6—C1—H1	109.1	C9—C8—H8	120.5
C3—C2—C1	112.9 (6)	C7—C8—H8	120.5
C3—C2—Br1	110.8 (5)	C8—C9—C10	121.6 (7)
C1—C2—Br1	107.8 (5)	C8—C9—H9	119.2
C3—C2—H2	108.4	C10—C9—H9	119.2
C1—C2—H2	108.4	C11—C10—C9	117.9 (7)
Br1—C2—H2	108.4	C11—C10—C13	122.6 (7)
C2—C3—C4	113.7 (7)	C9—C10—C13	119.5 (7)
C2—C3—H3A	108.8	C10—C11—C12	122.6 (6)
C4—C3—H3A	108.8	C10—C11—H11	118.7
C2—C3—H3B	108.8	C12—C11—H11	118.7
C4—C3—H3B	108.8	C11—C12—C7	118.4 (6)
H3A—C3—H3B	107.7	C11—C12—H12	120.8
C5—C4—C3	111.4 (7)	C7—C12—H12	120.8
C5—C4—H4A	109.3	C10—C13—H13A	109.5
C3—C4—H4A	109.3	C10—C13—H13B	109.5
C5—C4—H4B	109.3	H13A—C13—H13B	109.5
C3—C4—H4B	109.3	C10—C13—H13C	109.5
H4A—C4—H4B	108.0	H13A—C13—H13C	109.5
C4—C5—C6	111.5 (7)	H13B—C13—H13C	109.5
C4—C5—H5A	109.3		

Hydrogen-bond geometry (Å, º)

D—H···*A*	*D*—H	H···*A*	*D*···*A*	*D*—H···*A*
N1—H1*N*···O2^{i}	0.93	2.05	2.971 (7)	175.5
C2—H2···O1	0.98	2.57	3.248(9)	127
C12—H12···O1	0.93	2.54	2.906(8)	104

Symmetry code: (i) -*x*+1, -*y*+1, -*z*+1.
